# Chlokamycins B–D: Chlorohydrin-Containing Polycyclic Tetramate Macrolactams with Cytotoxic Activity from the Marine Sponge-Derived *Streptomyces xiamenensis* 1310KO-148

**DOI:** 10.3390/md24030117

**Published:** 2026-03-21

**Authors:** Min Ah Lee, Jong Soon Kang, Joo-Hee Kwon, Jeong-Wook Yang, Hwa-Sun Lee, Chang-Su Heo, Hee Jae Shin

**Affiliations:** 1 Marine Natural Products Chemistry Laboratory, Korea Institute of Ocean Science and Technology, 385 Haeyang-ro, Yeongdo-gu, Busan 49111, Republic of Korea; minah@kiost.ac.kr (M.A.L.); hwasunlee@kiost.ac.kr (H.-S.L.); science30@kiost.ac.kr (C.-S.H.); 2 Department of Chemistry, Pukyong National University, 45 Yongso-ro, Nam-Gu, Busan 48513, Republic of Korea; 3 Laboratory Animal Resource Center, Korea Research Institute of Bioscience and Biotechnology, 30 Yeongudanji-ro, Cheongwon-gu, Cheongju 28116, Republic of Korea; kanjon@kribb.re.kr (J.S.K.); juhee@kribb.re.kr (J.-H.K.); z7v8@kribb.re.kr (J.-W.Y.); 4 Department of Marine Biotechnology, University of Science and Technology (UST), 217 Gajeong-ro, Yuseong-gu, Daejeon 34113, Republic of Korea

**Keywords:** *Streptomyces xiamenensis*, marine, polycyclic tetramate macrolactam, chlorohydrin, chlokamycin, cytotoxicity

## Abstract

Chemical investigation of the marine sponge-derived *Streptomyces xiamenensis* 1310KO-148 afforded six polycyclic tetramate macrolactams (PTMs), including three known compounds (**1**–**3**) and three previously undescribed chlorohydrin-containing analogues, chlokamycins B–D (**4**–**6**). Their planar structures were elucidated by extensive analysis of 1D and 2D NMR spectra and HR-ESIMS data, while the relative configurations were assigned using NOESY correlations. The absolute configurations were further confirmed by electronic circular dichroism (ECD) calculations. Compounds **3**–**6** exhibited significant cytotoxic activity against 14 human cancer cell lines (GI_50_ = 2.68–24.92 μM) and antibacterial activity against *Staphylococcus aureus* (MIC = 16.00–32.00 μg/mL) and *Micrococcus luteus* (MIC = 4.00–32.00 μg/mL) among six tested bacterial strains.

## 1. Introduction

Tetramic acid-derived natural products constitute a prominent class of microbial secondary metabolites that are widely produced by bacteria and fungi, particularly members of the actinomycetes. These compounds are characterized by a 2,4-pyrrolidinedione scaffold and have attracted considerable interest because of their pronounced structural diversity and broad spectrum of biological activities, including antibacterial, antifungal, antiviral, and cytotoxic properties [1,2]. Structural diversification within this family commonly arises from various biosynthetic tailoring processes, generating multiple subclasses that exhibit distinct molecular frameworks and biological functions. Among these, polycyclic tetramate macrolactams (PTMs) constitute a structurally unique group of tetramic acid metabolites.

PTMs represent a distinct group of natural products characterized by a rigid three-dimensional framework comprising a large macrolactam ring, a tetramate core, and fused polycyclic rings. Ikarugamycin (**1**), the earliest identified compound in this class, was first isolated from *Streptomyces phaeochromogenes* in 1972 [3] and has been extensively studied for its strong antimicrobial and anticancer properties [4,5,6,7,8]. Subsequent studies have revealed that the structural and biological diversity of PTMs is expanded by diverse substituents and extensive modification processes, which collectively contribute to their pharmacological potential [8,9,10,11].

According to previous studies, capsimycin C from *S. xiamenensis* 318 bearing hydroxyl groups at the C-7 and C-8 positions (Figure S1) exhibited no cytotoxic activity at 10 μM against eight pancreatic cancer cell lines: HPAC, Patu8988, BxPC-3, PANC-1, AsPC-1, Capan-2, CFPAC-1, and MiaPaca-2 [12]. Similarly, hydroxyikarugamycin B from *Streptomyces* sp. SCSIO 40060 showed no cytotoxicity at 100 μM against SF268, NCI-H460, and HepG2 cell lines [13]. In particular, halogenated PTMs are extremely rare in nature. To date, only three chloride-containing PTMs have been reported, all of which were isolated from *Streptomyces* species: capsimycin D (**3**) [12] and chlokamycin [14], both possessing a 5/6/5 ring system, and pactamide F [8] with a 5/5/6 ring system (Figure S1). Cytotoxic activity has been reported for chlokamycin (IC_50_ = 24.7–33.5 μM against Jurkat and HCT116 cell lines) and pactamide F (IC_50_ = 2.65–2.85 μM against SF-268, MCF-7, NCI-H460, and Hep-G2 cell lines). The absolute configuration of chlokamycin remains unresolved, and pactamide F was produced from *S. pactum* SCSIO 02999 by promoter engineering and heterologous expression.

**Figure 1:**
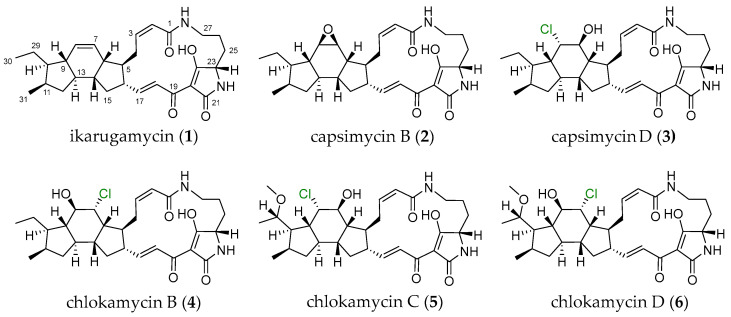
Structures of PTMs (**1**–**6**) produced by *S. xiamenensis* 1310KO-148.

Given the structural diversity, limited occurrence, and incomplete biological evaluation of chloride-containing PTMs, further studies are required to clarify their chemical characteristics and pharmacological potential. Herein, we describe the isolation and identification of the chlorohydrin-containing 5/6/5 ring system of PTMs (**3**–**6**) from *S. xiamenensis* 1310KO-148 (Figure 1). In addition, compounds **3**–**6** were evaluated for their cytotoxicity and antibacterial activity, and a structure–activity relationship (SAR) was examined based on the positions of the chlorine and methoxy substitutions.

## 2. Results

### 2.1. Structure Elucidation of New Compounds

Extraction of the culture broth of the marine-derived actinomycete *S. xiamenensis* 1310KO-148 with ethyl acetate (EtOAc) and chemical investigation of the extract afforded three previously undescribed chlorohydrin-containing PTMs (**4**–**6**) along with three known analogues (**1**–**3**). Compounds **1**–**3** were identified as ikarugamycin (**1**), capsimycin B (**2**), and capsimycin D (**3**), respectively. Their structures were confirmed by comparing their ^1^H and ^13^C NMR, 2D NMR, and MS data with previously reported data (Figures S2–S20 and Tables S1–S3) [12,15].

**Figure 2:**
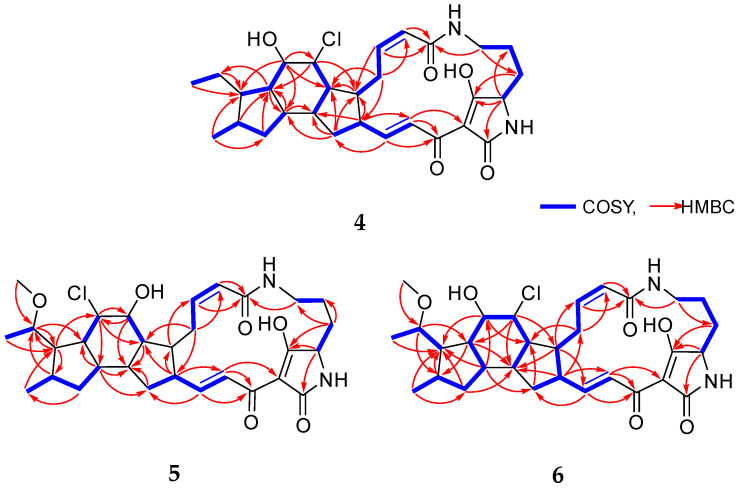
Key ^1^H–^1^H COSY and HMBC correlations of **4**–**6**.

Chlokamycin B (**4**) was obtained as a yellow amorphous powder. The molecular formula of **4** was deduced as C_29_H_39_N_2_O_5_Cl based on the HR-ESIMS peaks at *m*/*z* 531.2626 [M+H]^+^ (calcd for C_29_H_40_N_2_O_5_Cl, 531.2626) and 553.2446 [M+Na]^+^ (calcd for C_29_H_39_N_2_O_5_NaCl, 553.2445), indicating 11 degrees of unsaturation. Owing to the solubility issue of **4**, its 2D NMR spectra for structural analysis were acquired in 90% CDCl_3_/CD_3_OD (Table 1). The ^1^H NMR spectrum of **4** exhibited two types methyl protons (*δ*_H_ 0.81, d, *J* = 6.5 Hz; *δ*_H_ 0.87, t, *J* = 7.3, Hz), four olefinic protons (*δ*_H_ 5.79, d, *J* = 11.4 Hz; *δ*_H_ 6.00, t, *J* = 11.4 Hz; *δ*_H_ 6.76, dd, *J* = 15.4, 10.1 Hz; *δ*_H_ 7.07, d, *J* = 15.4 Hz), three heteroatom-connected methine protons (*δ*_H_ 3.81, dd, *J* = 4.8, 3.0 Hz; *δ*_H_ 3.91, dd, *J* = 7.6, 4.1 Hz; *δ*_H_ 4.10, t, *J* = 4.1 Hz), eight sp^3^ methine protons (*δ*_H_ 1.02, td, *J* = 11.4, 7.6 Hz; *δ*_H_ 1.52, m; *δ*_H_ 1.77, td, *J* = 11.4, 7.4 Hz; *δ*_H_ 1.83, m; *δ*_H_ 2.16, m; *δ*_H_ 2.21, m; *δ*_H_ 2.43, m; *δ*_H_ 2.52, m), and seven sp^3^ methylene protons (*δ*_H_ 0.61, 2.11, m; *δ*_H_ 1.01, 1.50, m; *δ*_H_ 1.26, 1.93, m; *δ*_H_ 1.32, 1.71, m; *δ*_H_ 1.73, 1.99, m; *δ*_H_ 2.50, 3.35, m; *δ*_H_ 2.54, 3.58, m) (Table 1 and Figure S20). From the ^13^C NMR and HSQC spectra, two methyl carbons (*δ*_C_ 13.2, C-30; *δ*_C_ 17.9, C-31), four olefinic carbons (*δ*_C_ 122.8, C-18; *δ*_C_ 123.8, C-2; *δ*_C_ 141.8, C-3; *δ*_C_ 151.8, C-17), three heteroatom-connected methine carbons (*δ*_C_ 61.6, C-23; *δ*_C_ 68.5, C-7; *δ*_C_ 76.3, C-8), eight sp^3^ methine carbons (*δ*_C_ 32.4, C-11; *δ*_C_ 43.1, C-13; *δ*_C_ 43.7, C-6; *δ*_C_ 45.3, C-5; *δ*_C_ 46.1, C-14; *δ*_C_ 49.6, C-16; *δ*_C_ 50.2, C-10; *δ*_C_ 52.6, C-9), seven sp^3^ methylene carbons (*δ*_C_ 21.2, C-26; *δ*_C_ 21.7, C-29; *δ*_C_ 27.0, C-4; *δ*_C_ 27.4, C-25; *δ*_C_ 36.6, C-15; *δ*_C_ 39.0, C-27; *δ*_C_ 39.1, C-12), and five nonprotonated sp^2^ carbons (*δ*_C_ 101.0, C-20; *δ*_C_ 167.2, C-1; *δ*_C_ 173.6, C-19; *δ*_C_ 175.6, C-21; *δ*_C_ 197.3, C-24) were identified (Table 1). The ^1^H and ^13^C NMR spectra of **4** closely resembled those of capsimycin D (**3**), which belongs to the 5/6/5 type of chloride-containing PTMs (Table 1, Table 2 and Table S3). A comprehensive comparison of the NMR data for **4** and **3** indicates that **4** differs from **3** in the positions of a chlorine atom (*δ*_H_ 4.10, t, *J* = 4.1 Hz; *δ*_C_ 68.5, C-7) and a hydroxy group (*δ*_H_ 3.91, dd, *J* = 7.6, 4.1 Hz; *δ*_C_ 76.3, C-8). This conclusion was further supported by the ^1^H–^1^H COSY correlations of H-6 (*δ*_H_ 2.52)/H-7 (*δ*_H_ 4.10), H-6 (*δ*_H_ 2.52)/H-14 (*δ*_H_ 1.83), H-8 (*δ*_H_ 3.91)/H-9 (*δ*_H_ 1.02), and H-9 (*δ*_H_ 1.02)/H-10 (*δ*_H_ 1.52). In addition, key HMBC correlations from H-7 (*δ*_H_ 4.10) to C-5 (*δ*_C_ 45.3)/C-9 (*δ*_C_ 52.6) and from H-8 (*δ*_H_ 3.91) to C-6 (*δ*_C_ 43.7)/C-7 (*δ*_C_ 68.5)/C-10 (*δ*_C_ 50.2)/C-13 (*δ*_C_ 43.1) further confirmed the structural assignment (Figure 2). Thus, the planar structure of **4** was identified as a new positional isomer of **3** (Figure 1).

**Figure 3:**
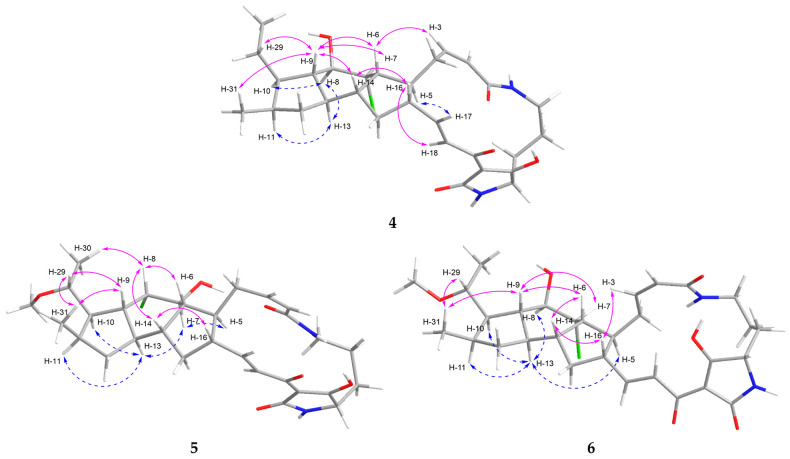
NOESY correlations of **4**–**6**.

The coupling constant between the olefinic protons H-2 (*δ*_H_ 5.79, *J* = 11.4 Hz) and H-3 (*δ*_H_ 6.00, *J* = 11.4 Hz) was indicative of a *Z*-configured double bond, whereas that between H-17 (*δ*_H_ 6.76, *J* = 15.4, 10.1 Hz) and H-18 (*δ*_H_ 7.07, *J* = 15.4 Hz) supported an *E*-configuration (Table 1). The relative configuration of the 5/6/5 tricyclic ring system in **4** was determined based on the NOESY spectrum. The NOE correlations of H-8 (*δ*_H_ 3.91) with H-10 (*δ*_H_ 1.52)/H-13 (*δ*_H_ 1.77), H-13 (*δ*_H_ 1.77) with H-8 (*δ*_H_ 3.91)/H-11 (*δ*_H_ 2.16), and H-5 (*δ*_H_ 2.21) with H-17 (*δ*_H_ 6.76) established that H-5, H-8, H-10, H-11, and H-13 were located on the same side of the 5/6/5 tricyclic unit (blue dashed curves for **4**, Figure 3). In contrast, the correlations of H-9 (*δ*_H_ 1.02) with H-6 (*δ*_H_ 2.52)/H-7 (*δ*_H_ 4.10)/H-14 (*δ*_H_ 1.83)/H-29 (*δ*_H_ 1.32, 1.71)/H-31 (*δ*_H_ 0.81), H-6 (*δ*_H_ 2.52) with H-3 (*δ*_H_ 6.00)/H-9 (*δ*_H_ 1.02), and H-16 (*δ*_H_ 2.43) with H-14 (*δ*_H_ 1.83)/H-18 (*δ*_H_ 7.07) indicated that H-6, H-7, H-9, H-14, and H-16 were positioned on the opposite side (pink curves for **4**, Figure 3). These spatial arrangements were further corroborated by coupling constant analysis. The large coupling constant between H-8 (dd, *J* = 7.6, 4.1 Hz) and H-9 (td, *J* = 11.4, 7.6 Hz), between H-9 (dd, *J* = 11.4, 7.6 Hz) and H-13 (td, *J* = 11.4, 7.4 Hz) was consistent with trans-diaxial orientation across the C-8/C-9 and C-9/C-13 bonds. Moreover, a key NOESY correlation of H-9 with H-7, along with the small coupling constant between H-7 (t, *J* = 4.1 Hz) and H-8 (dd, *J* = 7.6, 4.1 Hz), suggested a trans (axial–equatorial) orientation across the C-7/C-8 bond. These findings collectively implied *trans*–*cis*–*trans* A/B–B/C–C/D ring fusions [12,16]. Thus, the relative configuration of **4** was elucidated as 5S*,6R*,7R*,8R*,9S*,10R*,11R*,13R*,14R*,16S* for the 5/6/5 tricyclic unit of **4**.

**Figure 4:**
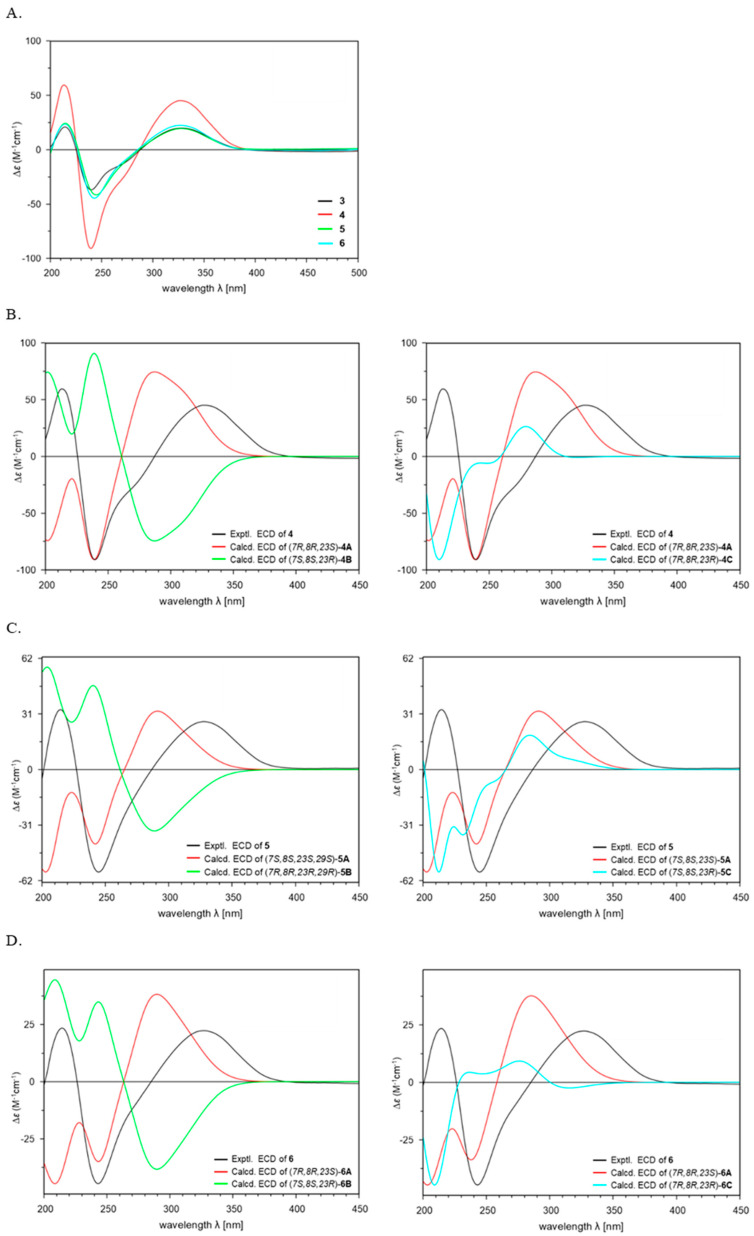
Comparison of the experimental ECD spectra of **3**–**6** (**A**). Experimental and Calculated ECD spectra of **4**–**6** (**B**–**D**).

To determine the absolute configuration of **4**, additional ECD calculations were conducted for three possible configurations of (5*S*,6*R*,7*R*,8*R*,9*S*,10*R*,11*R*,13*R*,14*R*,16*S*,23*S*)-**4A**, (5*R*,6*S*,7*S*,8*S*,9*R*,10*S*,11*S*,13*S*,14*S*,16*R*,23*R*)-**4B**, and (5*S*,6*R*,7*R*,8*R*,9*S*,10*R*,11*R*,13*R*,14*R*,16*S*,23*R*)-**4C** using the TDDFT at the B3LYP/6-311G+(d,p) level of method (Figure 4B). The results of the 5/6/5 tricyclic ring system in **4** demonstrated good agreement between the experimental ECD spectrum and the calculated spectrum of isomer **4A** with the (5*S*,6*R*,7*R*,8*R*,9*S*,10*R*,11*R*,13*R*,14*R*,16*S*,23*S*-**4**) configuration (Figure 4B). Furthermore, the absolute configuration at C-23 was assigned as 23*S* based on comparison with the calculated ECD spectra of conformers **4A** and **4C** (Figure 4B). In previously reported PTMs, the ornithine unit was derived from L-ornithine, as evidenced by the *S*-configuration at C-23 [9,15,17]. This fact supported the conclusion that the ornithine moiety in **4** also originates from L-ornithine. Thus, the structure of **4** was elucidated as a new isomer of **3**, and **4** was named chlokamycin B, a previously undescribed chlorohydrin-containing PTMs metabolite.

Chlokamycin C (**5**) was isolated as a yellow amorphous powder. The molecular formula of **5** was identified as C_30_H_40_N_2_O_6_Cl by the HR-ESIMS peaks at *m*/*z* 561.2733 [M+H]^+^ (calcd for C_30_H_42_N_2_O_6_Cl, 561.2731) and 583.2551 [M+Na]^+^ (calcd for C_30_H_41_N_2_O_6_NaCl, 583.2551), accounting for 11 degrees of unsaturation. Comparison of the NMR data for **5** with those of capsimycin D (**3**) demonstrated that their structures were highly similar. The key difference was the presence of a methoxy group (*δ*_H_ 3.30, s; *δ*_C_ 56.1; C-32) attached to C-29 (*δ*_C_ 78.7) in 5 (Table 1 and Table 2). The methylene carbon signal at C-29 (*δ*_C_ 22.3) in **3** was replaced by an oxygenated carbon (*δ*_C_ 78.7) in **5**. The location of the methoxy substituent was assigned to the C-29 position based on the HMBC correlation from H-32 (*δ*_H_ 3.30) to C-29 (*δ*_C_ 78.7), and the COSY correlation between H-29 (*δ*_H_ 3.43, m) and H-30 (*δ*_H_ 1.21, d, *J* = 6.2 Hz) (Figure 2).

The relatively small vicinal coupling constant (*J*_2,3_ = 11.5 Hz) across the C-2/C-3 double bond supported the assignment of a *Z*-configuration, whereas the large vicinal coupling constant (*J*_17,18_ = 15.4 Hz) across the C-17/C-18 double bond indicated an *E*-configuration (Table 1). The NOE correlations of H-5 (*δ*_H_ 2.09) with H-7 (*δ*_H_ 4.11) and H-13 (*δ*_H_ 1.65) with H-7 (*δ*_H_ 4.11)/H-10 (*δ*_H_ 2.08)/H-11 (*δ*_H_ 2.24) indicated that H-5, H-7, H-10, H-11, and H-13 were orientated in the same direction, as shown by the blue dashed curves (Figure 3). Conversely, the cross-peaks of H-8 (*δ*_H_ 4.32) with H-6 (*δ*_H_ 2.13)/H-14 (*δ*_H_ 2.07)/H-30 (*δ*_H_ 1.21), H-9 (*δ*_H_ 2.10) with H-29 (*δ*_H_ 3.43)/H-31 (*δ*_H_ 1.04), H-16 (*δ*_H_ 2.38) with H-14 (*δ*_H_ 2.07), and H-29 (*δ*_H_ 3.43) with H-9 (*δ*_H_ 2.10)/H-31 (*δ*_H_ 1.04) supported that H-6, H-8, H-9, H-14, H-16, and H-29 were located on the other side, as represented by the pink curves for **5** in Figure 3. Thus, these results suggested that **5** possesses a relative configuration of 5S*,6R*,7S*,8S*,9R*,10R*,11R*,13R*,14R*,16S*,29S*.

To determine the absolute configuration, theoretical calculations of the ECD spectra of **5** were performed. The calculated ECD spectra of **5** were generated for three possible stereoisomers (5*S,*6*R,*7*S,*8*S,*9*R,*10*R,*11*R,*13*R,*14*R,*16*S,*23*S,*29*S-***5A**; 5*R,*6*S,*7*R,*8*R,*9*S,*10*S,*11*S,*13*S,*14*S,*16*R,*23*R,*29*R*-**5B**; 5*S,*6*R,*7*S,*8*S,*9*R,*10*R,*11*R,*13*R,*14*R,*16*S,*23*R,*29*S*-**5C**) (Figure 4C). The absolute configuration of 5 (5*S,*6*R,*7*S,*8*S,*9*R,*10*R,*11*R,*13*R,*14*R,*16*S,*23*S,*29*S***)** was established by comparison of the calculated and experimental ECD spectra (Figure 4C). Accordingly, the structure of **5** was determined as 29-methoxy capsimycin D, and **5** was named chlokamycin C.

Chlokamycin D (**6**) was obtained as a yellow amorphous powder. Its molecular formula was established as C_30_H_41_N_2_O_6_Cl based on the HR-ESIMS peaks at *m*/*z* 561.2730 [M+H]^+^ (calcd for C_30_H_42_N_2_O_6_Cl, 561.2731) and 583.2550 [M+Na]^+^ (calcd for C_30_H_41_N_2_O_6_NaCl, 583.2551), corresponding to 11 degrees of unsaturation. The ^1^H and ^13^C NMR data of 6 closely resembled those of 5, with notable differences observed at the positions corresponding to a chlorine atom (*δ*_H_ 4.23, t, *J* = 5.0 Hz; *δ*_C_ 68.6, C-7) and a hydroxy group (*δ*_H_ 3.90, dd, *J* = 8.0, 5.0 Hz; *δ*_C_ 77.3, C-8) (Table 1 and Table 2). This assignment was further supported by HMBC correlations from H-7 (*δ*_H_ 4.23) to C-5 (*δ*_C_ 46.8)/C-9 (*δ*_C_ 51.8)/C-14 (*δ*_C_ 46.7), from H-8 (*δ*_H_ 3.99) to C-6 (*δ*_C_ 45.8)/C-10 (*δ*_C_ 52.2), and from H-13 (*δ*_H_ 1.87) to C-8 (*δ*_C_ 77.3)/C-10 (*δ*_C_ 52.2) (Figure 2). In addition, the presence of an extra set of signals corresponding to a methoxy group (*δ*_H_ 3.36, s; *δ*_C_ 56.3, C-32) in **6**, compared to those in **4**, indicated that **6** is a 29-methoxylated derivative of **4** (Table 1 and Table 2).

The configurations of the C-2 (*Z*, ∆^2,3^) and C-17 (*E*, ∆^17,18^) double bonds in **6** were determined from the coupling constants *J*_2,3_ = 11.3 Hz and *J*_17,18_ = 15.4 Hz, respectively (Table 1). The NOE correlations showed that **6** has the same relative configuration as **4**, featuring *trans*–*cis*–*trans* ring fusions (Figure 3). The relative configuration at C-29 was deduced as 29S* based on the NOE correlations of H-29 with H-31 and H-31 with H-9.

Theoretical ECD calculations were conducted on three possible stereoisomers of **6** (5*S*,6*R*,7*R*,8*R*,9*R*,10*R*,11*R*,13*R*,14*R*,16*S*,23*S*,29*S*-**6A**, 5*R*,6*S*,7*S*,8*S*,9*S*,10*S*,11*S*,13*S*,14*S*,16*R*,23*R*,29*R*-**6B**, and 5*S*,6*R*,7*R*,8*R*,9*R*,10*R*,11*R*,13*R*,14*R*,16*S*,23*R*,29*S*-**6C**) to assign the absolute configuration (Figure 4D). As shown in Figure 4D, the experimental ECD curve of **6** closely matched the calculated spectrum for the 5*S*,6*R*,7*R*,8*R*,9*R*,10*R*,11*R*,13*R*,14*R*,16*S*,23*S*,29*S*-**6A** isomer. Overall, these results indicated that **6** is a 29-methoxylated derivative of chlokamycin C (**5**), and **6** was designated chlokamycin D.

**Figure 5:**
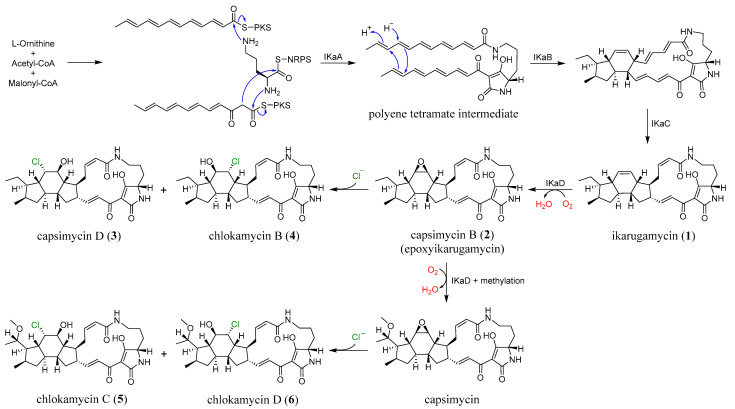
The plausible biosynthetic pathway of **1**–**6**.

The biosynthetic pathway of PTMs has been broadly described as involving the assembly of three distinct fragments: one derived from L-ornithine and two C_12_ chains originating from six acetate units, consistent with what is now recognized as a hybrid polyketide synthase-nonribosomal peptide synthetase (PKS-NRPS) system [9,11,17,18,19]. As illustrated in Figure 5, compounds **1**–**6** are proposed to originate from a common PTM biosynthetic pathway in *S. xiamenensis* 1310KO-148. The characteristic 5/6/5 tricyclic core of ikarugamycin (**1**), the first identified PTM, is plausibly biosynthesized through IkaA-mediated assembly of two PKS-derived polyketide chains via consecutive condensation reactions. The resulting intermediates are subsequently processed by the oxidoreductase IkaB, followed by a cyclization step involving a Diels–Alder reaction, in which the alcohol dehydrogenase IkaC is thought to participate indirectly. Furthermore, IkaD introduces two modifications to ikarugamycin (**1**): epoxidation of the C-7/C-8 double bond to yield capsimycin B (**2**), and hydroxylation followed by methylation at the C-29 position, potentially leading to the formation of capsimycin [12,13,20]. The epoxide moiety present in the intermediate **2** may serve as a key precursor for the formation of the chlorohydrin functionality observed in compounds **3**–**6**. In this scenario, nucleophilic attack by chloride on the oxirane ring would result in regioselective epoxide opening and subsequent formation of the corresponding chlorohydrin derivatives. Considering the conformational rigidity of the PTM framework, such nucleophilic attack is likely to occur from the axial direction of the epoxide. This stereochemical course is consistent with the Fürst–Plattner rule, which predicts preferential trans-diaxial ring opening of cyclic epoxides by nucleophiles [21]. Although the specific enzyme responsible for this transformation has not yet been identified in the genome of *S*. *xiamenensis* 1310KO-148, similar enzymatic processes leading to epoxide-to-halohydrin conversion have been suggested in the biosynthetic pathways of several halogenated natural products [22,23,24,25]. From the 70 L culture extract of strain 1310KO-148, compounds **3** (42.4 mg), **4** (8.0 mg), **5** (13.2 mg), and **6** (7.4 mg) were isolated. These results suggest that chlorohydrin formation at the C-7/C-8 double bond of the PTMs produced by this strain occurs regioselectively, with a preference for chlorination at C-8.

### 2.2. Bioactivity Evaluation of Compounds

The biological activities of **3**–**6** were summarized in Table 3 and Table 4. Cytotoxicity was evaluated against six solid and eight blood cancer cell lines (Table 3 and Tables S16 and S17). Compounds **3**–**6** showed significant cytotoxicity across all 14 cancer cell lines. In the SAR analysis, **3** (GI_50_ = 2.68–17.82 μM) and **4** (GI_50_ = 3.78–19.58 μM), which lack a methoxy group at C-29, demonstrated greater cytotoxicity than **5** (GI_50_ = 4.95–13.90 μM) and **6**. Furthermore, compounds **3** (GI_50_ = 2.68–17.82 μM) and **5** (GI_50_ = 4.95–13.90 μM), bearing a chlorine atom at the C-8 position, exerted superior anticancer activity compared to **4** (GI_50_ = 3.78–19.58 μM) and **6** (GI_50_ = 10.36–24.92 μM), which are chlorinated at C-7. These findings suggest that the presence and regioselective positioning of the chlorine substituent at C-7 and C-8 significantly influence the cytotoxic activity of the PTMs, indicating a possible SAR associated with the halogenation pattern.

The antibacterial activities of **3**–**6** were tested against six bacterial strains, comprising three Gram-positive and three Gram-negative species (Table 4). Compounds **3** and **4** exhibited antibacterial activity against *Staphylococcus aureus*, with minimum inhibitory concentrations (MICs) of 32.0 and 16.0 μg/mL, respectively. Compounds **3**–**6** exhibited notable activity against *Micrococcus luteus*, with MIC values ranging from 4.0 to 32.0 μg/mL. In the antibacterial assay against *M. luteus*, **3** and **4**, which lack a methoxy group at C-29, exhibited significant activity (MIC = 4.00 μg/mL). Additionally, **4** and **6**, bearing a chlorine atom at C-7, showed relatively enhanced antibacterial activity against both *S. aureus* (MIC = 16.00 μg/mL) and *M. luteus* (MIC = 16.00 μg/mL) compared to **3** (MIC = 32.00 μg/mL) and **5** (MIC = 32.00 μg/mL), which are chlorinated at C-8.

## 3. Materials and Methods

### 3.1. General Experimental Procedures and Reagents

Optical rotations were measured with a Rudolph analytical Autopol III S2 polarimeter (Rudolph Research Analytical, Hackettstown, NJ, USA). UV spectra were recorded with a Shimadzu UV-1650PC spectrophotometer (Shimadzu Corporation, Kyoto, Japan). IR spectra were obtained on an OPUS FT/IR-ALPHA II spectrophotometer (Bruker OPTIK GmbH & Co. KG, Ettlingen, Germany). LR-ESIMS data were obtained with an ISQ EM mass spectrometer. HR-ESIMS data were obtained with a Waters SYNPT G2 Q-TOF mass spectrometer (Waters Corporation, Milford, MA, USA) at Korea Basic Science Institute (KBSI) in Cheongju, Republic of Korea, with samples dissolved in MeOH prior to analysis. NMR spectra were acquired with a Bruker AVANCE III 600 spectrometer (Bruker Biospin GmbH, Rheinstetten, Germany) with a 3 mm probe operating at 600 MHz (^1^H) and 150 MHz (^13^C). Chemical shifts were expressed in ppm with reference to the solvent peaks (*δ*_H_ 3.31 and *δ*_C_ 49.15 ppm for CD_3_OD; *δ*_H_ 7.26 and *δ*_C_ 77.25 ppm for 90% CDCl_3_/CD_3_OD). ECD spectra were recorded with a JASCO J-1500 circular dichroism spectrometer (JASCO Corporation, Tokyo, Japan) at the Gyeongnam Bio and Anti-aging Core Facility Center for Changwon National University in Changwon, Republic of Korea. HPLC was performed using a BLS-Class pump (Teledyne SSI, Inc., State College, PA 16803, USA) with a Shodex RI-201H refractive index detector (Shoko Scientific Co. Ltd., Yokohama, Japan). Columns for HPLC were YMC-Triart C_18_ (250 mm × 10 mm, 5 μm), YMC-Triart C_8_ (250 mm × 10 mm, 5 μm), and YMC-CHIRAL PREP CD PM (250 mm × 4.6 mm, S-10 μm). RP silica gel (YMC-Gel ODS-A, 12 nm, S-75 μm) was used for open-column chromatography. Organic solvents were purchased as HPLC-grade, and ultrapure water was obtained from a Milipore Mili-Q Direct 8 system. The reagents used in the bioassay were purchased from Sigma-Aldrich (St. Louis, MO, USA), and Tokyo Chemical Industry (Tokyo, Japan).

### 3.2. Strain and Fermentation

The marine-derived strain 1310KO-148 was isolated from a sponge sample collected at Kosrae State, Federated States of Micronesia, in October 2013 (5°20′14.78″ N 162°56′41.32″ E). The strain was identified as *Streptomyces xiamenensis* based on 16S rRNA gene sequence analysis. The GenBank accession number is PP863885.

The strain 1310KO-148 was inoculated into modified Bennett’s broth medium (0.1% D-glucose, 0.02% tryptone, 0.01% yeast extract, 0.01% beef extract, 0.05% glycerol, sea salt 32 g/L, pH 7.0). The seed culture was incubated at 28 °C for 14 days at 120 rpm and aseptically transferred into a 100 L fermenter containing 70 L of sterilized culture medium (0.1% *v*/*v*). The culture was fermented at 28 °C with agitation at 40 rpm and an airflow rate of 10 L/min (LPM) for 16 days.

### 3.3. Extraction and Isolation of Compounds ***3**–**6***

The 70 L fermentation broth was extracted twice with ethyl acetate (EtOAc, 70 L each) and concentrated under reduced pressure to yield a crude extract (6.9 g). The extract was subjected to ODS column chromatography (YMC Gel ODS-A, 12 nm, S75 μm) followed by stepwise gradient elution with MeOH/H_2_O (*v*/*v*) (ranging from 20:80 to 100:0) as eluent to obtain 10 fractions (Fr.1–Fr.10). The Fr.10 (199.0 mg) was further fractionated on a column of silica gel (Merck silica gel 60, 6 nm, 40–63 μm) by eluting with n-hexane, CH_2_Cl_2_, and EtOAc to afford three subfractions (Fr.10.1–Fr.10.3), including pure **1** (Fr.10.3, 48.8 mg). The 90% MeOH fraction (Fr.8, 637 mg) was suspended in MeOH and extracted with n-hexane to yield MeOH- and n-hexane-soluble layers. The MeOH layer (412.5 mg) was separated into four subfractions (Fr.8.1–Fr.8.4) via an ODS column chromatography (MeOH/H_2_O, 80–100%). A semi-preparative reversed-phase HPLC (YMC-Triart C_18_ column, 250 mm × 10 mm i.d., 5 μm; 80% MeOH in H_2_O; flow rate: 2.0 mL/min; detector: RI) was used to divide the Fr.8.2 fraction (217.0 mg) into 21 subfractions (Fr.8.2.1–Fr.8.2.34), including pure **3** (42.4 mg, *t*_R_ 41.0 min). Compound **2** was purified from the Fr.8.2.11 (4.2 mg) by a semi-preparative RP HPLC (YMC-ODS A C_18_ column, 250 mm × 10 mm i.d., 5 μm; 65% MeCN in H_2_O; flow rate: 1.5 mL/min; detector: RI) to yield **2** (1.3 mg, *t*_R_ 26.0 min). The Fr.8.2.15 (20.6 mg) was purified by a semi-preparative RP HPLC (YMC-Pack C_8_ column, 250 mm × 10 mm i.d., 5 μm; 75% MeOH in H_2_O; flow rate: 2.0 mL/min; detector: RI) to yield **4** (8.0 mg, *t*_R_ 44.0 min). The 80% MeOH fraction (Fr.7, 375.0 mg) was separated into four subfractions (Fr.7.1–Fr.7.5) via ODS column chromatography (MeOH/H_2_O, 70–100%). A semi-preparative reversed-phase HPLC (YMC-Pack C_8_ column, 250 mm ×10 mm i.d., 5 μm; 70% MeOH in H_2_O; flow rate: 2.0 mL/min; detector: RI) was used to divide the Fr.7.3 (255.0 mg) into 25 subfractions (Fr.7.3.1–Fr.7.3.25). The Fr.7.3.7 (33.4 mg) was purified using a semi-preparative reversed-phase HPLC (YMC-ODS A C_18_ column, 250 mm × 10 mm i.d., 5 μm; 65% MeCN in H_2_O; flow rate: 1.5 mL/min; detector: RI) to yield **5** (13.2 mg, *t*_R_ 18.0 min). Compound **6** was purified from the Fr.7.3.9 (49.3 mg) by a semi-preparative reversed-phase HPLC (YMC-ODS A C_18_ column, 250 mm × 10 mm i.d., 5 μm; 65% MeCN in H_2_O; flow rate: 1.5 mL/min; detector: RI) to yield **6** (7.4 mg, *t*_R_ 22.0 min).

Capsimycin D (**3**): Yellow amorphous powder; [α${]}_{D}^{20}$: +168.3 (*c* 0.2, MeOH); UV (MeOH) *λ*_max_ (logԑ): 236 (3.85), 320 (3.83) nm; ECD (MeOH, *λ* [nm] (∆*ԑ*), *c* = 0.75 mM) 328 (+19.84), 240 (−36.91), 214 (+20.91); IR (MeOH) *ν*_max_ 3307, 2940, 1647, 1579, 1438, 1244 cm^−1^; ^1^H and ^13^C NMR data see Table 1 and Table 2; HRESIMS *m*/*z* 531.2626 [M+H]^+^ (calcd for C_29_H_40_N_2_O_5_Cl, 531.2626) and 553.2444 [M+Na]^+^ (calcd for C_29_H_39_N_2_O_5_NaCl, 553.2445).

Chlokamycin B (**4**): Yellow amorphous powder; [α${]}_{D}^{20}$: +150.0 (*c* 0.2, MeOH); UV (MeOH) *λ*_max_ (logԑ): 232 (2.95), 320 (2.74) nm; ECD (MeOH, *λ* [nm] (∆*ԑ*), *c* = 1.13 mM) 326 (+45.07), 240 (−90.75), 213 (+59.47); IR (MeOH) *ν*_max_ 3311, 2933, 1650, 1605, 1459, 1236, 1028 cm^−1^; ^1^H and ^13^C NMR data see Table 1 and Table 2; HRESIMS *m*/*z* 531.2626 [M+H]^+^ (calcd for C_29_H_40_N_2_O_5_Cl, 531.2626) and 553.2446 [M+Na]^+^ (calcd for C_29_H_39_N_2_O_5_NaCl, 553.2445).

Chlokamycin C (**5**): Yellow amorphous powder; [α${]}_{D}^{20}$: +110.0 (*c* 0.2, MeOH); UV (MeOH) *λ*_max_ (logԑ): 230 (3.92), 320 (3.77) nm; ECD (MeOH, *λ* [nm] (∆*ԑ*), *c* = 0.36 mM) 328 (+20.01), 242 (−44.84), 216 (+26.88); IR (MeOH) *ν*_max_ 3307, 2929, 1647, 1579, 1448, 1025 cm^−1^; ^1^H and ^13^C NMR data see Table 1 and Table 2; HRESIMS *m*/*z* 561.2733 [M+H]^+^ (calcd for C_30_H_42_N_2_O_6_Cl, 561.2731) and 583.2551 [M+Na]^+^ (calcd for C_30_H_41_N_2_O_6_NaCl, 583.2551).

Chlokamycin D (**6**): Yellow amorphous powder; [α${]}_{D}^{20}$: +106.6 (*c* 0.2, MeOH); UV (MeOH) *λ*_max_ (logԑ): 230 (4.04), 324 (3.91) nm; ECD (MeOH, *λ* [nm] (∆*ԑ*), *c* = 0.36 mM) 324 (+22.91), 242 (−48.57), 213 (+26.28); IR (MeOH) *ν*_max_ 3303, 2933, 1647, 1583, 1448, 1244, 1028 cm^−1^; ^1^H and ^13^C NMR data see Table 1 and Table 2; HRESIMS *m*/*z* 561.2730 [M+H]^+^ (calcd for C_30_H_42_N_2_O_6_Cl, 561.2731) and 583.2550 [M+Na]^+^ (calcd for C_30_H_41_N_2_O_6_NaCl, 583.2551).

### 3.4. ECD Calculations

The preliminary geometry optimization and conformational search were performed using Conflex 8 [26]. The optimization and calculation for electronic circular dichroism (ECD) were conducted utilizing the Gaussian 16 programme (rev. B.01, Gaussian Inc., Wallingford, CT, USA). Conformational searches were carried out using MMFF94s force field calculations, with a search threshold of 5 kcal/mol. The conformers of **4**–**6** were optimized using the ground state DFT geometry optimization method at the B3LYP/6-311G+(d,p) level in MeOH with an IEFPCM model for ECD. The theoretical calculations of ECD spectra were performed using TD-SCF at the B3LYP/6-311G+(d,p) (NStates = 60). The calculated ECD spectra of the conformers were Boltzmann-weighted and averaged using SpecDis 1.71 software with a *σ* value of 0.28 eV.

### 3.5. Cytotoxic Assay

The cytotoxic activity of **3**–**6** was evaluated using the CellTiter-Glo^®^ luminescent cell viability assay (Promega, Madison, WI, USA) and the sulforhodamine B (SRB) assay, following previously reported protocols [27]. Cancer cell lines were purchased from the Japanese Cancer Research Resources Bank (JCRB, Osaka, Japan). Luminescence signals were recorded with a GloMax^®^ Multi Detection System (Promega, Madison, WI, USA), and GI_50_ values were calculated using the relative GI_50_ model implemented in GraphPad Prism 10 (GraphPad Software, San Diego, CA, USA). Doxorubicin served as the positive control.

### 3.6. Antibacterial Assay

The antibacterial activity was evaluated using the 96-well microplate method, as described in a published report [27], against three Gram-positive bacteria (*Staphylococcus aureus* KCTC1927, *Bacillus subtilis* KCTC1021, and *Micrococcus luteus* KCTC1915) and three Gram-negative bacteria (*Escherichia coli* KCTC2441, *Salmonella typhimurium* KCTC2515, and *Klebsiella pneumoniae* KCTC2690). The six bacterial species were cultured in Müller Hinton (MH) broth medium at 30 °C and 140 rpm for 24 h. The 0.5 McFarland bacterial suspension (1 × 10^8^ CFU/mL) was diluted to 5 × 10^6^ CFU/mL. Aliquots of 100 μL of the diluted suspension and 100 μL of Mueller–Hinton (MH) broth containing compounds **3**–**6** were dispensed into each well of a 96-well plate. Following incubation at 30 °C for 24 h, minimum inhibitory concentrations (MICs) were determined by measuring the optical density at 610 nm (OD_610_). Kanamycin was used as a positive control.

## 4. Conclusions

In summary, three previously unreported chlorohydrin-containing PTMs (**4**–**6**) were isolated and structurally characterized from *S. xiamenensis* 1310KO-148 obtained from a marine sponge. Comparison of the ECD spectra of **3**–**6** revealed a high degree of similarity in their Cotton effects (Figure 4A). In addition, all four compounds (**3**–**6**) exhibited positive optical rotation values, suggesting that variations in substitution at C-7, C-8, and C-29 have little influence on either the ECD spectra or optical rotation. Notably, potent anticancer activity was observed for capsimycin D (**3**), whose cytotoxicity has not been previously reported, as well as for the three newly isolated PTMs (**4**–**6**), with GI_50_ values ranging from 2.68 to 24.92 μM. SAR analysis revealed that both the position of the chlorine substitution at C-7/C-8 and the presence of a methoxy group at C-29 significantly influenced the cytotoxic activity, suggesting that structural differences can significantly influence their biological activity. Overall, these findings expand the chemical diversity and potential bioactivity of halogen-containing PTMs and provide new insights into the SAR of this class of compounds.

## Figures and Tables

**Table 1:** ^1^H NMR (600 MHz) data of **3**–**6**.

Position	3	4		5	6
	*δ*_H_ ^a^ (*J* in Hz)	*δ*_H_ ^a^ (*J* in Hz)	*δ*_H_ ^b^ (*J* in Hz)	*δ*_H_ ^a^ (*J* in Hz)	*δ*_H_ ^a^ (*J* in Hz)
2	5.85, d (11.5)	5.88, d (11.3)	5.79, d (11.4)	5.85, d (11.5)	5.88, d (11.3)
3	6.06, td (11.5, 4.0)	6.11, td (11.3, 2.8)	6.00, t (11.4)	6.06, td (11.5, 3.8)	6.15, t (11.3)
4	2.51, m	2.49, m	2.50, m	2.51, m	2.68, m
	3.47, m	3.49, m	3.35, m	3.48, m	3.51, m
5	2.13, m	2.29, m	2.21, m	2.09, m	2.29, m
6	2.09, m	2.63, m	2.52, m	2.13, m	2.66, m
7	4.11, t (3.0)	4.17, t (3.7)	4.10, t (4.1)	4.11, t (3.0)	4.23, t (5.0)
8	4.24, t (3.0)	3.99, dd (7.3, 3.4)	3.91, dd (7.6, 4.1)	4.32, t (3.4)	3.90, dd (8.0, 5.0)
9	1.70, m	1.11, td (11.4, 7.2)	1.02, td (11.4, 7.6)	2.10, m	1.47, td (11.4, 8.0)
10	1.79, m	1.58, m	1.52, m	2.08, m	2.05, m
11	2.21, m	2.24, m	2.16, m	2.24, q (8.4, 7.9)	2.85, m
12	0.76, td (10.2, 6.2)	0.74, td (11.0, 5.2)	0.61, m	0.78, td (11.7, 8.8)	0.75, q (11.3)
	2.23, m	2.21, m	2.11, m	2.11, m	2.08, m
13	1.65, m	1.96, m	1.77, td (11.4, 7.4)	1.65, m	1.87, m
14	2.08, m	1.93, m	1.83, m	2.07, m	1.97, m
15	1.30, m	1.32, m	1.26, m	1.30, m	1.35, m
	2.11, m	1.97, m	1.93, m	2.11, m	2.02, m
16	2.38, m	2.50, m	2.43, m	2.38, q (10.1, 9.8)	2.51, m
17	6.76, dd (15.4, 10.2)	6.75, dd (15.4, 10.1)	6.76, dd (15.4, 10.1)	6.75, dd (15.4, 10.1)	6.78, dd (15.4, 10.2)
18	7.16, d (15.4)	7.23, d (15.4)	7.07, d (15.4)	7.18, d (15.4)	7.19, d (15.4)
23	3.85, dd (5.6, 2.1)	3.84, m	3.81, dd (4.8, 3.0)	3.84, dd (5.5, 1.6)	3.86, brs
25	1.89, m	1.84, m	1.73, m	1.85, m	1.83, m
	2.00, m	2.02, m	1.99, m	2.00, m	2.01, m
26	1.23, m	1.22, m	1.01, m	1.24, m	1.23, m
	1.55, m	1.53, m	1.50, m	1.55, m	1.52, m
27	2.67, ddd (13.6, 10.4, 2.8)	2.63, m	2.54, m	2.67, ddd (13.1, 10.2, 2.6)	2.67, m
	3.41, ddd (13.6, 5.5, 3.4)	3.45, m,	3.58, m	3.41, m	3.41, m
29	1.37, m	1.40, m	1.32, m	3.43, m	3.60, m
		1.76, m	1.71, m		
30	0.96, t (7.4)	0.97, t (7.4)	0.87, t (7.3)	1.21, d (6.2)	1.27, d (6.2)
31	0.92, d (6.6)	0.90, d (6.8)	0.81, d (6.5)	1.04, d (7.1)	1.01, d (7.1)
32				3.30, s	3.36, s

^a^ Measured in CD_3_OD; ^b^ Measured in 90% CDCl_3_/CD_3_OD.

**Table 2:** ^13^C NMR (150 MHz) data of **3**–**6**.

Position	3 ^a^	4 ^b^	5 ^a^	6 ^a^
	*δ*_C_, Type	*δ*_C_, Type	*δ*_C_, Type	*δ*_C_, Type
1	169.0, C	167.2, C	169.0, C	169.0, C
2	124.9, CH	123.8, CH	124.9, CH	124.8, CH
3	142.7, CH	141.8, CH	142.6, CH	143.1, CH
4	27.7, CH_2_	27.0, CH_2_	27.7, CH_2_	28.5, CH_2_
5	47.1, CH	45.3, CH	47.1, CH	46.8, CH
6	48.9, CH	43.7, CH	48.1, CH	45.8, CH
7	74.6, CH	68.5, CH	74.6, CH	68.6, CH
8	65.6, CH	76.3, CH	66.6, CH	77.3, CH
9	48.1, CH	52.6, CH	43.3, CH	51.8, CH
10	46.2, CH	50.2, CH	48.6, CH	52.2, CH
11	33.9, CH	32.4, CH	35.3, CH	36.4, CH
12	39.8, CH_2_	39.1, CH_2_	40.6, CH_2_	40.9, CH_2_
13	42.5, CH	43.1, CH	42.5, CH	44.8, CH
14	43.8, CH	46.1, CH	45.3, CH	46.7, CH
15	36.6, CH_2_	36.6, CH_2_	36.7, CH_2_	37.4, CH_2_
16	51.3, CH	49.6, CH	51.2, CH	51.2, CH
17	152.4, CH	151.8, CH	152.3, CH	151.5, CH
18	124.3, CH	122.8, CH	124.6, CH	124.7, CH
19	175.1, C	173.6, C	173.7, C	174.5, C
20	102.4, C	101.0, C	102.5, C	102.5, C
21	177.1, C	175.6, C	177.2, C	177.1, C
22, NH				
23	62.9, CH	61.6, CH	62.9, CH	63.0, CH
24	198.4, C	197.3, C	198.4, C	198.4, C
25	28.5, CH_2_	27.4, CH_2_	28.5, CH_2_	28.4, CH_2_
26	22.4, CH_2_	21.2, CH_2_	22.2, CH_2_	22.3, CH_2_
27	40.2, CH_2_	39.0, CH_2_	40.2, CH_2_	40.2, CH_2_
28, NH				
29	22.3, CH_2_	21.7, CH_2_	78.7, CH	78.7, CH
30	13.3, CH_3_	13.2, CH_3_	17.7, CH_3_	17.8, CH_3_
31	18.2, CH_3_	17.9, CH_3_	18.5, CH_3_	18.4, CH_3_
32			56.1, CH_3_	56.3, CH_3_

^a^ Measured in CD_3_OD; ^b^ Measured in 90% CDCl_3_/CD_3_OD.

**Table 3:** Cytotoxicity of **3**–**6** against solid and blood cancer cell lines.

Cancer Cell Lines		GI_50_, μM				
		3	4	5	6	Adriamycin
Solid cancer	ACHN	3.55	5.00	5.70	15.95	0.25
	MDA-MB-231	3.28	6.24	4.95	16.69	0.30
	HCT-15	3.47	4.93	5.79	15.54	0.29
	PC-3	3.25	4.59	5.39	14.46	0.23
	NUGC-3	2.81	3.78	4.49	11.45	0.24
	NCL-H23	3.33	5.16	5.99	11.01	0.28
Blood cancer	RPMI-1788	17.82	15.63	13.90	23.04	0.14
	HL-60	6.83	8.00	8.14	24.92	0.04
	K562	15.65	19.58	10.38	22.76	0.10
	NALM6	2.68	3.96	7.57	10.36	0.01
	Raji	5.39	7.11	9.22	24.50	0.02
	RPMI-8402	3.06	4.19	8.01	11.06	0.02
	U266	4.41	7.97	9.05	24.67	0.10
	WSU-DLCL2	8.97	11.55	9.94	24.13	0.02

ACHN, renal; MDA-MB-231, breast; HCT-15, colon; PC-3, prostate; NUGC-3, stomach; NCI-H23, lung; RPMI-1788, B lymphocyte cell; HL-60, acute myelogenous leukemia; K562, chronic myelogenous leukemia; NALM6, B cell acute lymphocytic leukemia; Raji, Burkitt’s lymphoma; RPMI-8402, T cell acute lymphocytic leukemia; U266, multiple myeloma; WSU-DLCL2, diffuse large B cell lymphoma.

**Table 4:** Antibacterial activities of **3**–**6**.

Gram-Type	Strains	MIC, μg/mL				
		3	4	5	6	Kanamycin
Gram-positive	*S. aureus*	32.00	16.00	>32.00	>32.00	0.12
	*B. subtilis*	>32.00	>32.00	>32.00	>32.00	0.50
	*M. luteus*	4.00	4.00	32.00	16.00	8.00
Gram-negative	*E. coli*	>32.00	>32.00	>32.00	>32.00	1.00
	*S. typhimurium*	>32.00	>32.00	>32.00	>32.00	4.00
	*K. pneumoniae*	>32.00	>32.00	>32.00	>32.00	2.00

## Data Availability

The data presented in this study are available upon request from the corresponding author.

## Supplementary Materials

The following supporting information can be downloaded at: https://www.mdpi.com/article/10.3390/md24030117/s1, Figure S1: PTMs; Figures S2–S6: ^1^H, ^13^C NMR, HSQC, COSY, HR-ESIMS data, and Structure of **1**; Figures S7–S11: ^1^H, ^13^C NMR, HSQC, LR-ESIMS data, and Structure of **2**; Figures S12–S20: ^1^H, ^13^C NMR, HSQC, COSY, HMBC, HR-ESIMS, IR, UV data, and Structure of **3**; Figures S21–S29: ^1^H, ^13^C NMR, DEPT, HSQC, COSY, HMBC, NOESY, HR-ESIMS, IR, and UV data of **4**; Figures S30–S38: ^1^H, ^13^C NMR, DEPT, HSQC, COSY, HMBC, NOESY, HR-ESIMS, IR, and UV data of **5**; Figures S39–S46: ^1^H, ^13^C NMR, DEPT, HSQC, COSY, HMBC, NOESY, HR-ESIMS, IR, and UV data of **6**; Tables S1–S3: ^1^H and ^13^C NMR data of **1**–**3**; Tables S4–S15: TDSCF-ECD calculation data of **4**–**6**; Tables S16 and S17: Cytotoxicity of **3**–**6.**
