# Supplementary material for: Chlokamycins B–D: Chlorohydrin-Containing Polycyclic Tetramate Macrolactams with Cytotoxic Activity from the Marine Sponge-Derived Streptomyces xiamenensis 1310KO-148
Source: Mar Drugs. 2026 Mar 21;24(3):117. doi: 10.3390/md24030117 (PMC13027431; doi:10.3390/md24030117)
Supplement: Supplementary file 1 [file marinedrugs-24-00117-s001.zip › marinedrugs-4213852-supplementary.pdf]

Supporting Information

Chlokamycins B–D: chlorohydrin-containing polycyclic tetramate macrolactams with cytotoxic activities from the marine sponge-derived *Streptomyces xiamenensis* 1310KO-148.

Min Ah Lee [1,2], Jong Soon Kang [3], Joo-Hee Kwon [3], Jeong-Wook Yang [3], Hwa-Sun Lee [1,2], Chang-Su Heo [1,4] and Hee Jae Shin [1,4,*]

1 Marine Natural Products Chemistry Laboratory, Korea Institute of Ocean Science and Technology, 385 Haeyang-ro, Yeongdo-gu, Busan 49111, Republic of Korea
2 Department of Chemistry, Pukyong National University, 45 Yongso-ro, Nam-Gu, Busan 48513, Republic of Korea; parksj@pknu.ac.kr
3 Laboratory Animal Resource Center, Korea Research Institute of Bioscience and Biotechnology, 30 Yeongudanji-ro, Cheongwon-gu, Cheongju 28116, Republic of Korea
4 Department of Marine Biotechnology, University of Science and Technology (UST), 217 Gajeong-ro, Yuseong-gu, Daejeon 34113, Republic of Korea
* Correspondence: shinhj@kiost.ac.kr; Tel.: +82-51-664-3341; Fax: +82-51-664-3340

CONTENTS

Figure S1. ^{1}H NMR spectrum of **1** (600 MHz, in 90% $CDCl_3/CD_3OD$). 4
Figure S2. ^{13}C NMR spectrum of **1** (150 MHz, in 90% $CDCl_3/CD_3OD$). 4
Figure S3. HSQC spectrum of **1** (in 90% $CDCl_3/CD_3OD$). 5
Figure S4. HR-ESIMS spectrum of **1**. 6
Figure S5. Structure of ikarugamycin (**1**). 7
Figure S6. ^{1}H NMR spectrum of **2** (600 MHz, in $CDCl_3$). 8
Figure S7. ^{13}C NMR spectrum of **2** (150 MHz, in $CDCl_3$). 8
Figure S8. HSQC spectrum of **2** (in $CDCl_3$). 9
Figure S9. LR-ESIMS spectrum of **2**. 9
Figure S10. Structure of capsimycin B (**2**). 10
Figure S11. ^{1}H NMR spectrum of **3** (600 MHz, in CD_3OD). 11
Figure S12. ^{13}C NMR spectrum of **3** (150 MHz, in CD_3OD). 11
Figure S13. HSQC spectrum of **3** (in CD_3OD). 12
Figure S14. ^{1}H-^{1}H COSY spectrum of **3** (in CD_3OD). 12
Figure S15. HMBC spectrum of **3** (in CD_3OD). 13
Figure S16. HR-ESIMS spectrum of **3**. 14
Figure S17. IR spectrum of **3**. 15
Figure S18. UV spectrum of **3**. 15
Figure S19. Structure of capsimycin D (**3**). 16
Figure S20. ^{1}H NMR spectrum of **4** (600 MHz, in 90% $CDCl_3/CD_3OD$). 17
Figure S21. ^{13}C and DEPT NMR spectrum of **4** (150 MHz, in 90% $CDCl_3/CD_3OD$). 17
Figure S22. HSQC spectrum of **4** (in 90% $CDCl_3/CD_3OD$). 18
Figure S23. ^{1}H-^{1}H COSY spectrum of **4**. 18
Figure S24. HMBC spectrum of **4**. 19
Figure S25. NOESY spectrum of **4**. 19
Figure S26. HR-ESIMS spectrum of **4**. 20
Figure S27. IR spectrum of **4**. 21
Figure S28. UV spectrum of **4**. 21
Figure S29. ^{1}H NMR spectrum of **5** (600 MHz, in CD_3OD). 22
Figure S30. ^{13}C NMR spectrum of **5** (150 MHz, in CD_3OD). 22
Figure S31. HSQC spectrum of **5** (in CD_3OD). 23
Figure S32. ^{1}H-^{1}H COSY spectrum of **5**. 23
Figure S33. HMBC spectrum of **5**. 24
Figure S34. NOESY spectrum of **5**. 24
Figure S35. HR-ESIMS spectrum of **5**. 25
Figure S36. IR spectrum of **5**. 26
Figure S37. UV spectrum of **5**. 26
Figure S38. ^{1}H NMR spectrum of **6** (600 MHz, in CD_3OD). 27
Figure S39. ^{13}C NMR (150 MHz, in CD_3OD) and DEPT spectra of **6**. 27
Figure S40. HSQC spectrum of **6** (in CD_3OD). 28

Figure S41. ^{1}H-^{1}H COSY spectrum of **6**. 28
Figure S42. HMBC spectrum of **6**. 29
Figure S43. NOESY spectrum of **6**. 29
Figure S44. HR-ESIMS spectrum of **6**. 30
Figure S45. IR spectrum of **6**. 31
Figure S46. UV spectrum of **6**. 31

Table S1. ^{1}H and ^{13}C NMR data of **1** and ikarugamycin (in 90% $CDCl_3/CD_3OD$). 7
Table S2. ^{1}H and ^{13}C NMR data of **2** and Capsimycin B (in $CDCl_3$). 10
Table S3. ^{1}H and ^{13}C NMR data of **3** and Capsimycin D. 16
Table S4. Conformers and Boltzmann distributions of the optimized **4** 32
Table S5. The Cartesian coordinates of the lowest-energy conformers of (5*S*,6*R*,7*R*,8*R*,9*S*,10*R*,11*R*,13*R*,14*R*,16*S*,23*S*)-**4A** 33
Table S6. The Cartesian coordinates of the lowest-energy conformers of (5*R*,6*S*,7*S*,8*S*,9*R*,10*S*,11*S*,13*S*,14*S*,16*R*,23*R*)-**4B** 34
Table S7. The Cartesian coordinates of the lowest-energy conformers of (5*S*,6*R*,7*R*,8*R*,9*S*,10*R*,11*R*,13*R*,14*R*,16*S*,23*R*)-**4C** 35
Table S8. Conformers and Boltzmann distributions of the optimized **5** 36
Table S9. The Cartesian coordinates of the lowest-energy conformers of (5*S*,6*R*,7*S*,8*S*,9*R*,10*R*,11*R*,13*R*,14*R*,16*S*,23*S*,29*S*)-**5A** 37
Table S10. The Cartesian coordinates of the lowest-energy conformers of (5*R*,6*S*,7*R*,8*R*,9*S*,10*S*,11*S*,13*S*,14*S*,16*R*,23*R*,29*R*)-**5B** 38
Table S11. The Cartesian coordinates of the lowest-energy conformers of (5*S*,6*R*,7*S*,8*S*,9*R*,10*R*,11*R*,13*R*,14*R*,16*S*,23*R*,29*S*)-**5C** 39
Table S12. Conformers and Boltzmann distributions of the optimized **6** 40
Table S13. The Cartesian coordinates of the lowest-energy conformers of (5*S*,6*R*,7*R*,8*R*,9*R*,10*R*,11*R*,13*R*,14*R*,16*S*,23*S*,29*S*)-**6A** 41
Table S14. The Cartesian coordinates of the lowest-energy conformers of (5*R*,6*S*,7*S*,8*S*,9*S*,10*S*,11*S*,13*S*,14*S*,16*R*,23*R*,29*R*)-**6B** 42
Table S15. The Cartesian coordinates of the lowest-energy conformers of (5*S*,6*R*,7*R*,8*R*,9*R*,10*R*,11*R*,13*R*,14*R*,16*S*,23*R*,29*S*)-**6C** 43
Table S16. Cytotoxicity of **3**–**6** against solid cancer cell lines. 44
Table S17. Cytotoxicity of **3**–**6** against blood cancer cell lines. 45

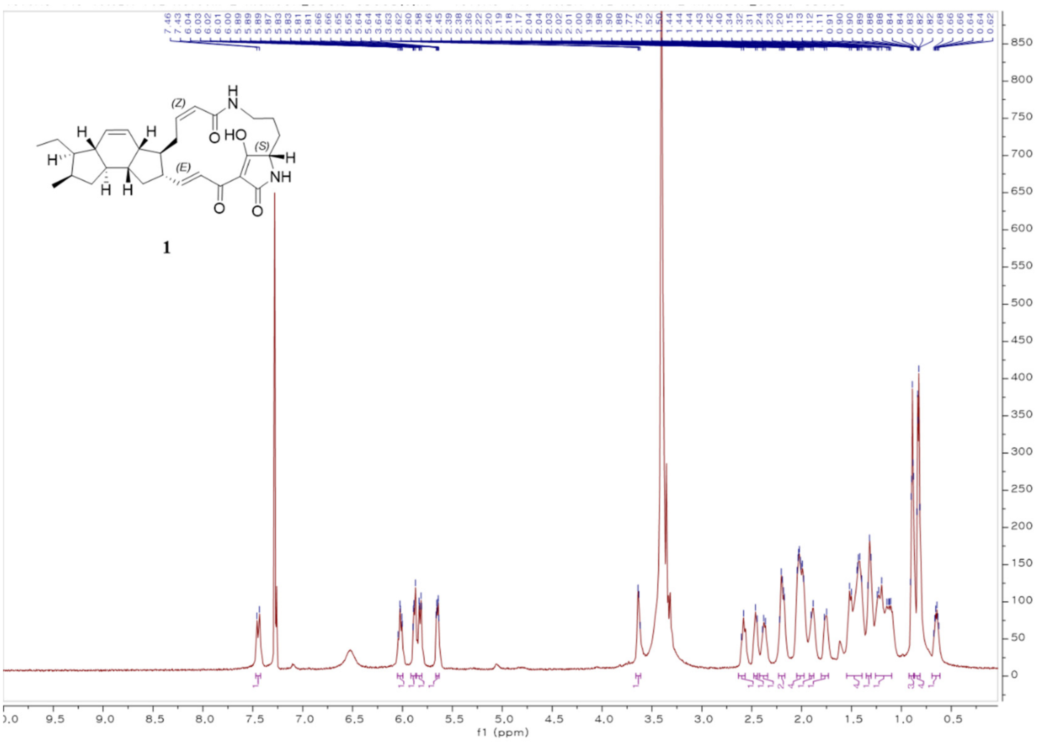

Figure S1. ^{1}H NMR spectrum of **1** (600 MHz, in 90% $CDCl_3/CD_3OD$).

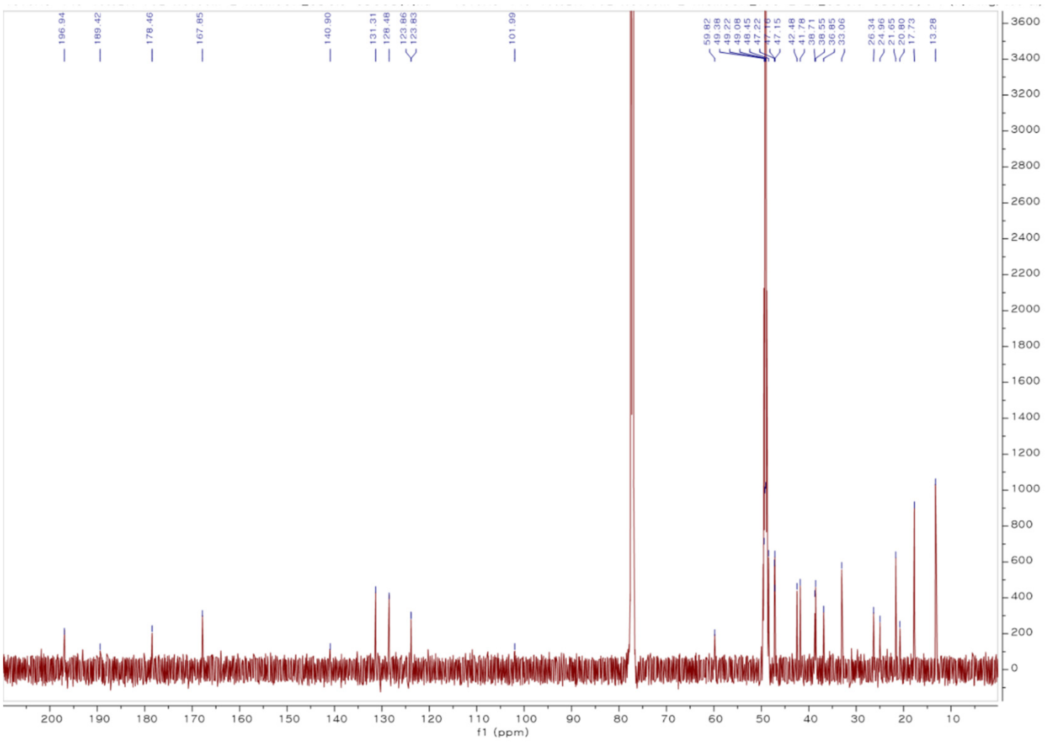

Figure S2. ^{13}C NMR spectrum of **1** (150 MHz, in 90% $CDCl_3/CD_3OD$).

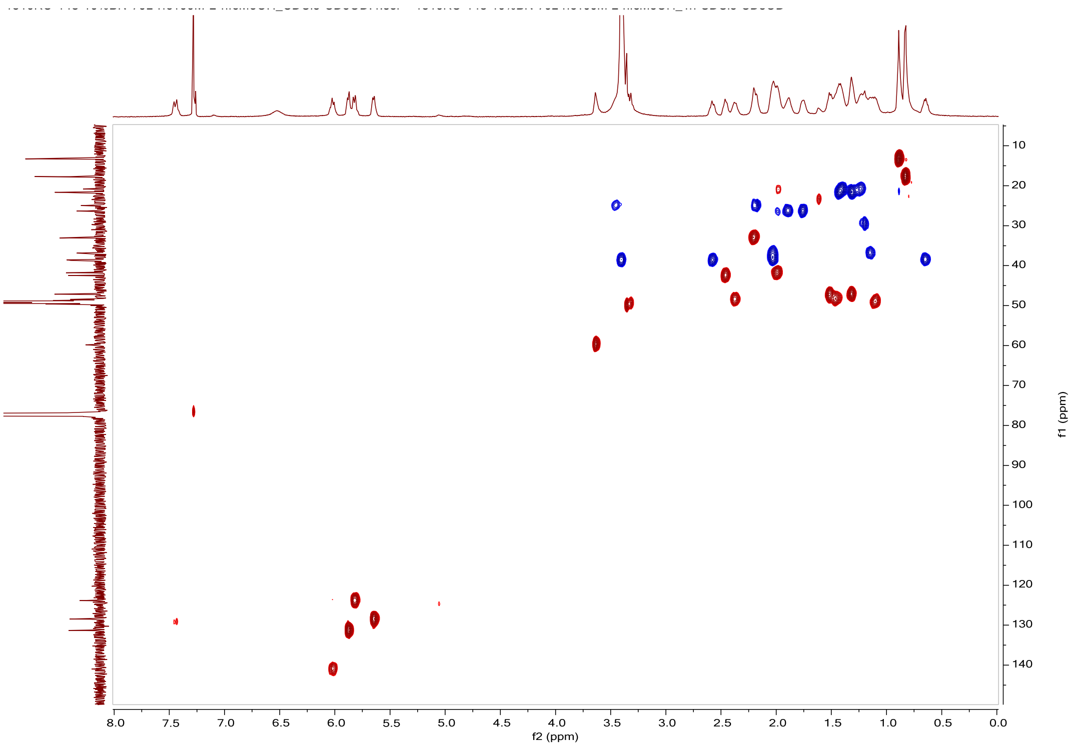

Figure S3. HSQC spectrum of **1** (in 90% $CDCl_3/CD_3OD$).

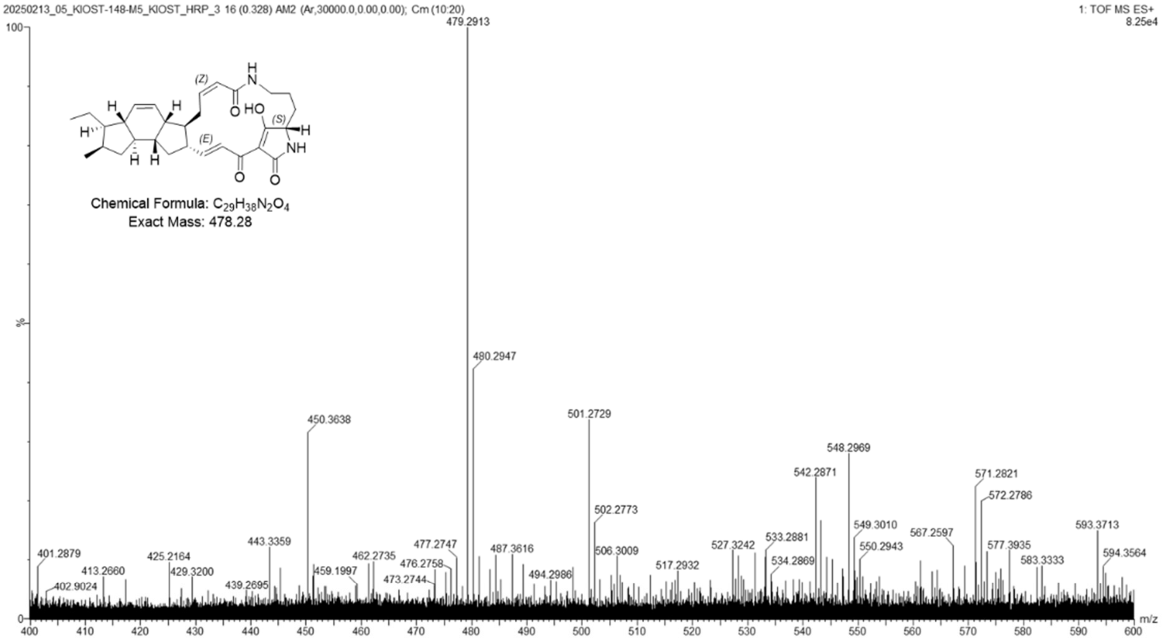

Elemental Composition Report

Single Mass Analysis
Tolerance = 5.0 PPM / DBE: min = -5.0, max = 50.0
Element prediction: Off
Number of isotope peaks used for i-FIT = 3

Monoisotopic Mass, Even Electron Ions
Elements Used:
C: 0-33 H: 0-180 N: 0-10 O: 0-10 Na: 0-1

Minimum:				-5.0				
Maximum:		5.0	5.0	50.0				
Mass	Calc. Mass	mDa	PPM	DBE	i-FIT	Norm	Conf(%)	Formula
479.2913	479.2910	0.3	0.6	11.5	998.2	0.983	37.40	C29 H39 N2 O4

Figure S4. HR-ESIMS spectrum of **1**.

Table S1. ^{1}H and ^{13}C NMR data of **1** and ikarugamycin (in 90% $CDCl_3/CD_3OD$).

Position	**1**		**Ikarugamycin**[a]	
	δ_H, Mult (*J* in Hz)	δ_C, Mult	δ_H, Mult (*J* in Hz)	δ_C, Mult
1		167.8, C		167.6, C
2	5.82, d (11.4)	123.9, CH	5.81, d (10.5)	123.7, CH
3	6.02, dd (11.5, 11.3)	140.9, CH	6.02, dd (10.5, 10.5)	140.8, CH
4	2.17, m<br>3.45, m	25.0, CH_2	2.15, m<br>3.50, m	24.7, CH_2
5	1.46, m	48.4, CH	1.45, m	48.5, CH
6	2.46, m	42.5, CH	2.45, m	42.3, CH
7	5.64, d (9.4)	128.5, CJ	5.64, d (10.0)	128.3, CH
8	5.87, d (9.6)	131.3, CH	5.87, d (10.0)	131.2, CH
9	1.52, m	47.1, CH	1.50, m	47.1, CH
10	1.32, m	47.2, CH	1.30, m	46.9, CH
11	2.20, m	33.1, CH	2.20. m	32.9, CH
12	0.65, m<br>2.03, m	38.5, CH_{22}	0.66, m<br>2.05, m	38.4, CH_2
13	1.10, m	48.8, CH	1.09, m	48.8, CH
14	1.99, m	41.8, CH	2.04, m	41.7, CH
15	1.15, m<br>2.03, m	36.8, CH_2	2.05, m	36.7, CH_2
16	2.38, m	49.8, CH	2.38, m	48.3, CH
17	6.53, bs	145.8, CH	6.55, dd (15.0, 10.0)	146.7, CH
18	7.43, d (14.0)	123.8, CH	7.43, d (15.0)	129.0. CH
19		189.4, CH		183.5, CH
20		102.0, CH		103.2, CH
21		178.5, C		178.1, C
22-NH				
23	3.63, m	59.8, CH	3.63, m	59.6, CH
24		196.9, C		196.7, C
25	1.76, m<br>1.89, m	26.3, CH_2	1.74, m<br>1.98, m	26.2, CH_2
26		20.8, CH_2	1.48, m<br>1.34, m	20.6, CH_2
27	2.58, m<br>3.40, m	38.7, CH_2	2.58, m<br>3.42, m	38.5, CH_2
28-NH				
29	1.31, m<br>1.42, m	21.6, CH_2	1.30, m<br>1.45, m	21.5, CH_2
30	0.89, t (7.1)	13.3, CH_3	0.89, t (7.0)	13.1, CH_3
31	0.83, d (6.8)	17.7, CH_3	0.82, d (7.0)	17.5, CH_3

[a] Measured in 90% $CDCl_3/CD_3OD$ (*Angew. Chem. Int. Ed.* **2014**, 53, 4840–4844).

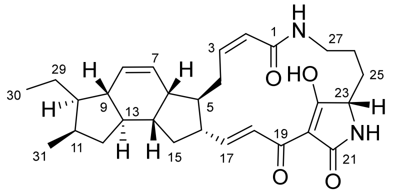

Figure S5. Structure of ikarugamycin (**1**).

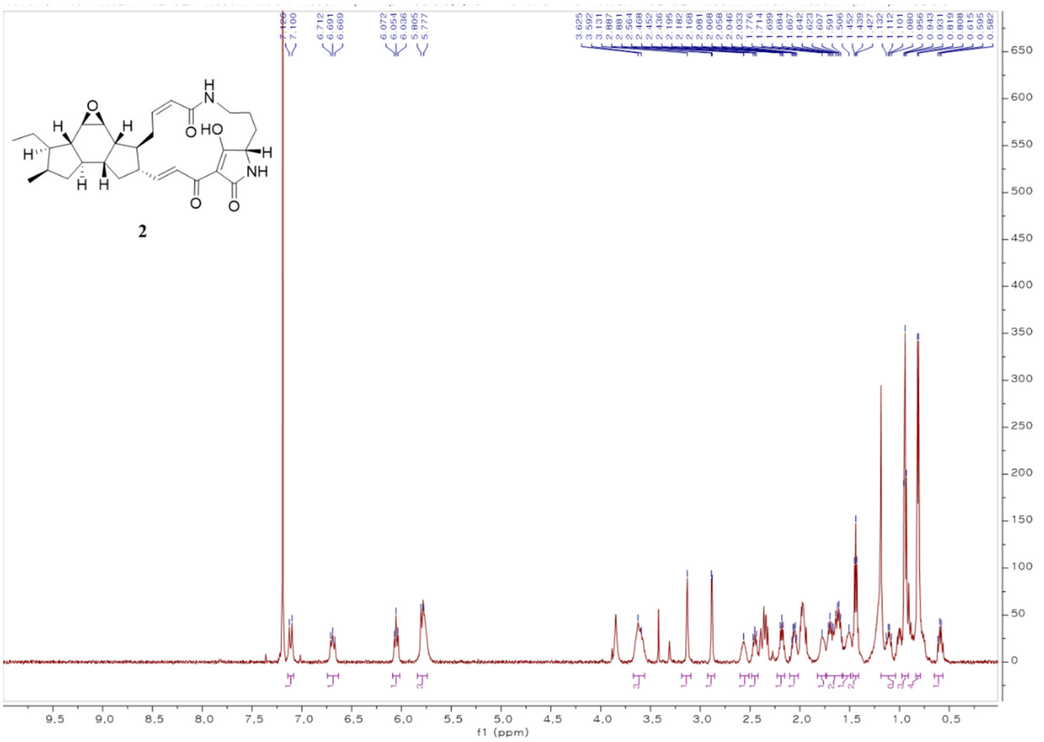

Figure S6. ^{1}H NMR spectrum of **2** (600 MHz, in CDCl$_3$).

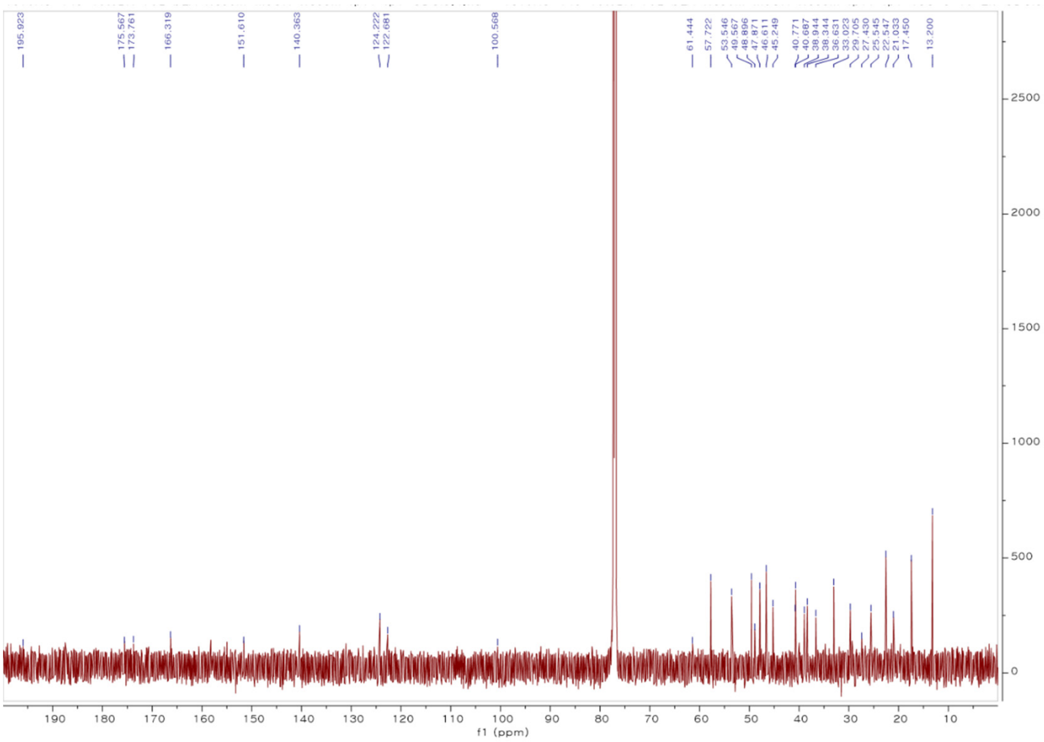

Figure S7. ^{13}C NMR spectrum of **2** (150 MHz, in CDCl$_3$).

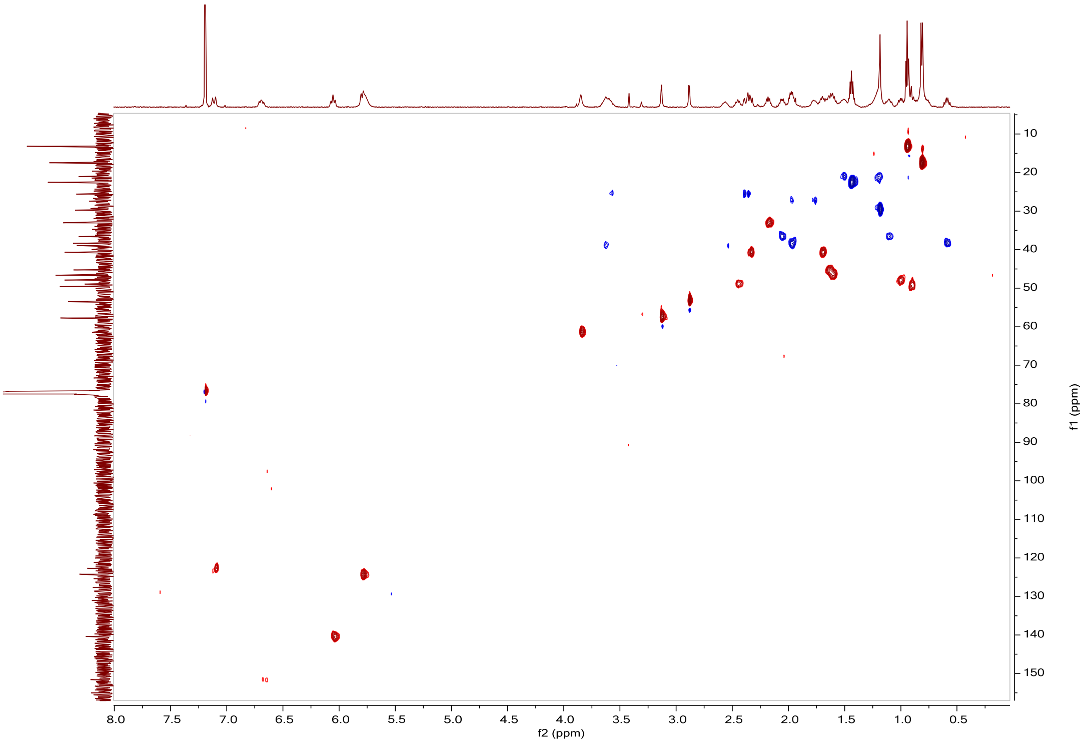

Figure S8. HSQC spectrum of **2** (in $CDCl_3$).

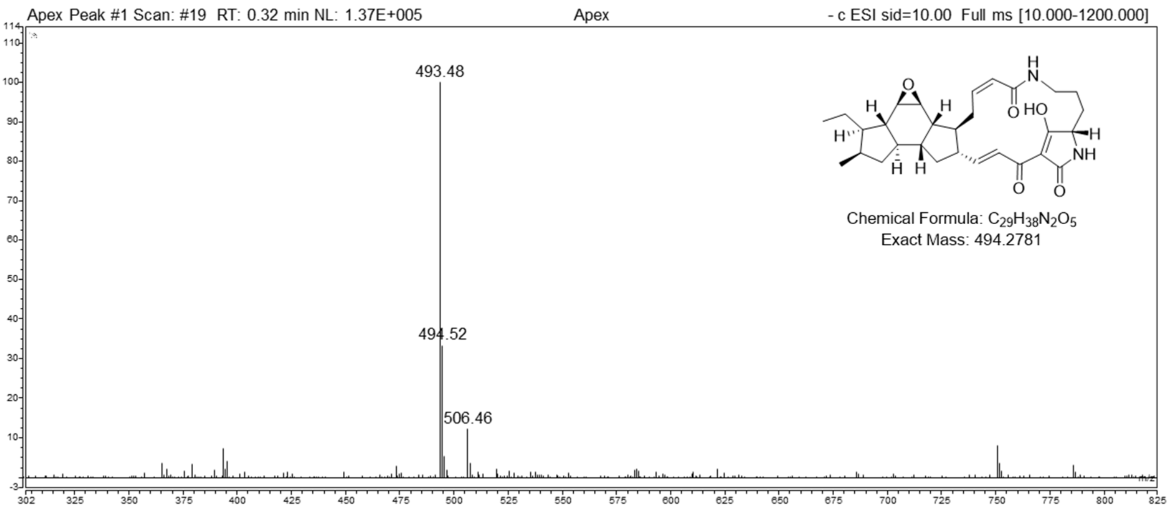

Figure S9. LR-ESIMS spectrum of **2**.

Table S2. ^{1}H and ^{13}C NMR data of **2** and Capsimycin B (in $CDCl_3$).

Position	**2**		**Capsimycin B**[a]	
	δ_H, Mult (*J* in Hz)	δ_C, Mult	δ_H, Mult (*J* in Hz)	δ_C, Mult
1		166.3, C		166.3, C
2	5.79, d (11.6)	124.2, CH	5.79, dd (11.9, 2.0)	124.2, CH
3	6.05, t (11.1)	140.4, CH	6.06, ddd (11.6, 11.6, 2.5)	140.4, CH
4	2.37, m<br>3.59, m	25.5, CH_2	2.38, m<br>3.61, m	25.5, CH_2
5	1.63, m	45.2, CH	1.65, m	45.2, CH
6	2.35, m	40.8, CH	2.34, m	40.8, CH
7	2.88, d (3.9)	53.5, CH	2.89, d (3.8)	53.5, CH
8	3.13, brs	57.7, CH	3.13, dd (3.8, 2.0)	57.7, CH
9	0.90, m	49.6, CH	0.91, m	49.6, CH
10	1.60, m	46.6, CH	1.60, m	46.6, CH
11	2.17, m	33.0, CH	2.18, m	33.0, CH
12	0.59, m<br>1.97, m	38.3, CH_2	0.59, m<br>1.96, m	38.3, CH_2
13	1.00, m	47.9, CH	1.01, m	47.9, CH
14	1.69, m	40.7, CH	1.70, m	40.7, CH
15	1.11, m<br>2.06, m	36.6, CH_2	1.11, m<br>2.06, m	36.6, CH_2
16	2.45, m	48.9, CH	2.46, m	48.9, CH
17	6.69, dd (15.5, 10.5)	151.6, CH	6.70, dd (15.6, 10.2)	151.6, CH
18	7.11, d (15.5)	122.7, CH	7.11, d (15.6)	122.7, CH
19		173.8, C		173.7, C
20		100.6, C		100.6, C
21		175.6, C		175.5, C
22-NH				
23	3.85, brs	61.4, CH	3.85, br d (3.7)	61.4, CH
24		195.9, C		195.9, C
25	1.77, m<br>1.97, m	27.4, CH_2	1.77, m<br>1.98, m	27.4, CH_2
26	1.20, m<br>1.50, m	21.0, CH_2	1.20, m<br>1.51, m	21.0, CH_2
27	2.54, m<br>3.63, m	38.9, CH_2	2.56, br t (11.4)<br>3.63, m	38.9, CH_2
28-NH				
29	1.44, m	22.5, CH_2	1.44, m	22.5, CH_2
30	0.94, t (7.4)	13.2, CH_3	0.94, d (7.3)	13.2, CH_3
31	0.81, d (7.0)	17.4, CH_3	0.81, d (7.3)	17.5, CH_3

[a] Measured in $CDCl_3$ (*Scientific Reports*, **2017**, 7, 40689).

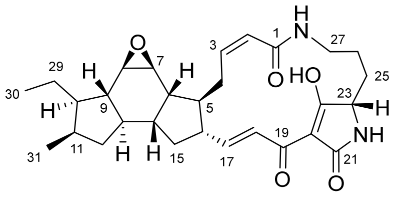

Figure S10. Structure of capsimycin B (**2**).

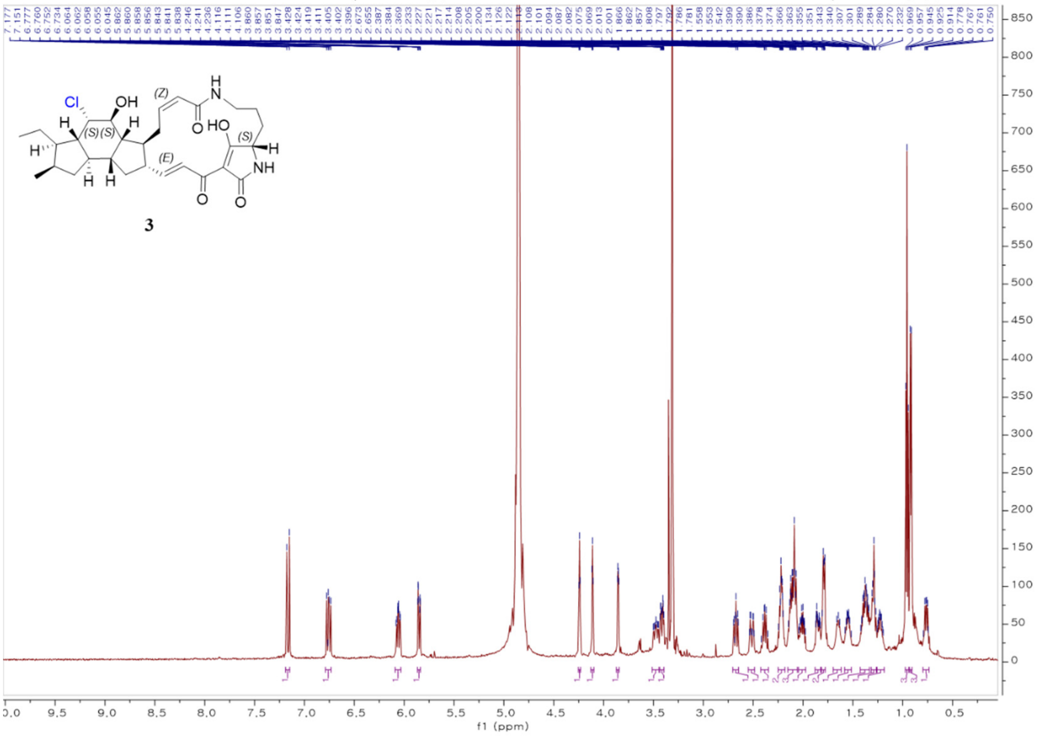

Figure S11. ^{1}H NMR spectrum of **3** (600 MHz, in CD_3OD).

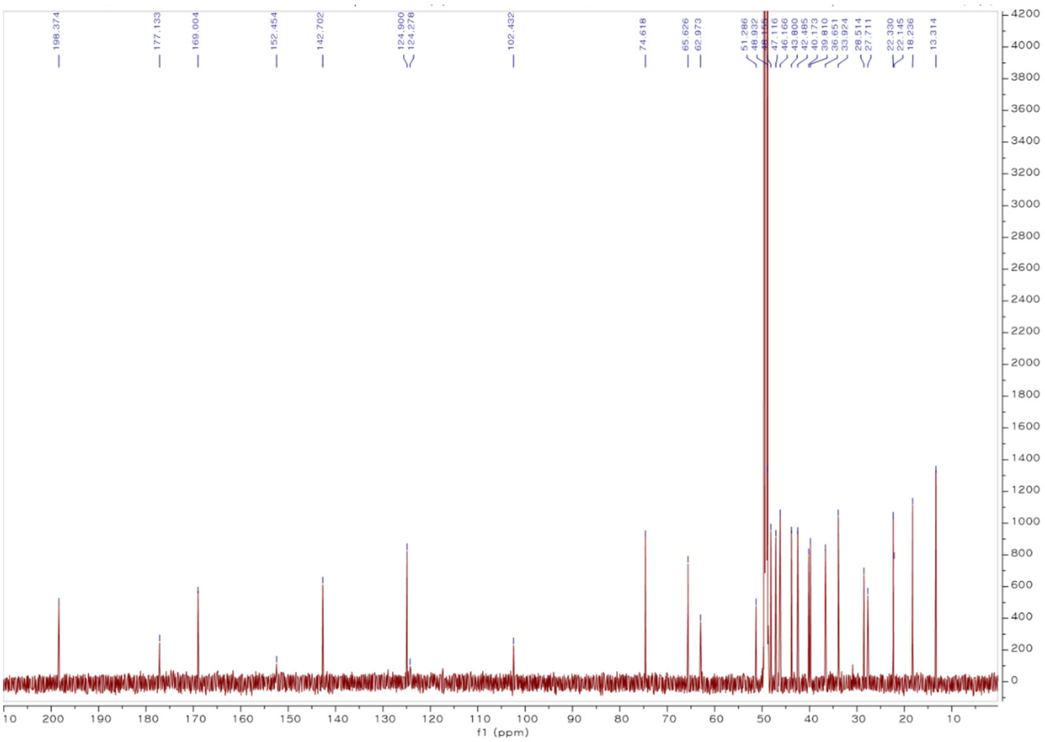

Figure S12. ^{13}C NMR spectrum of **3** (150 MHz, in CD_3OD).

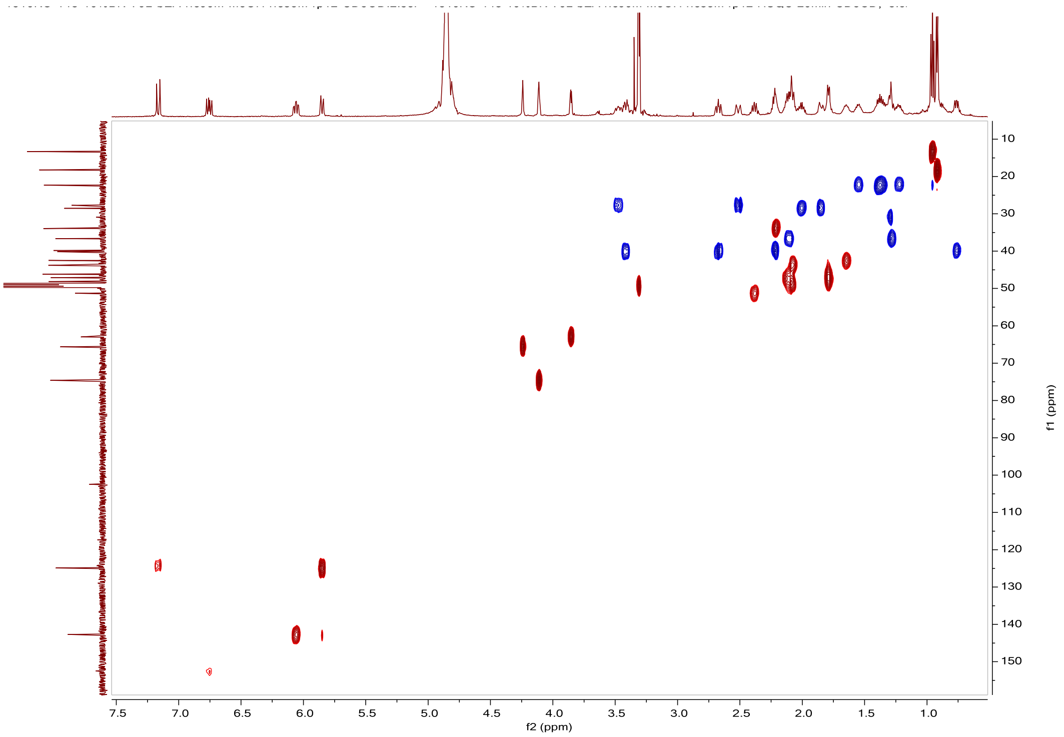

Figure S13. HSQC spectrum of **3** (in CD_3OD).

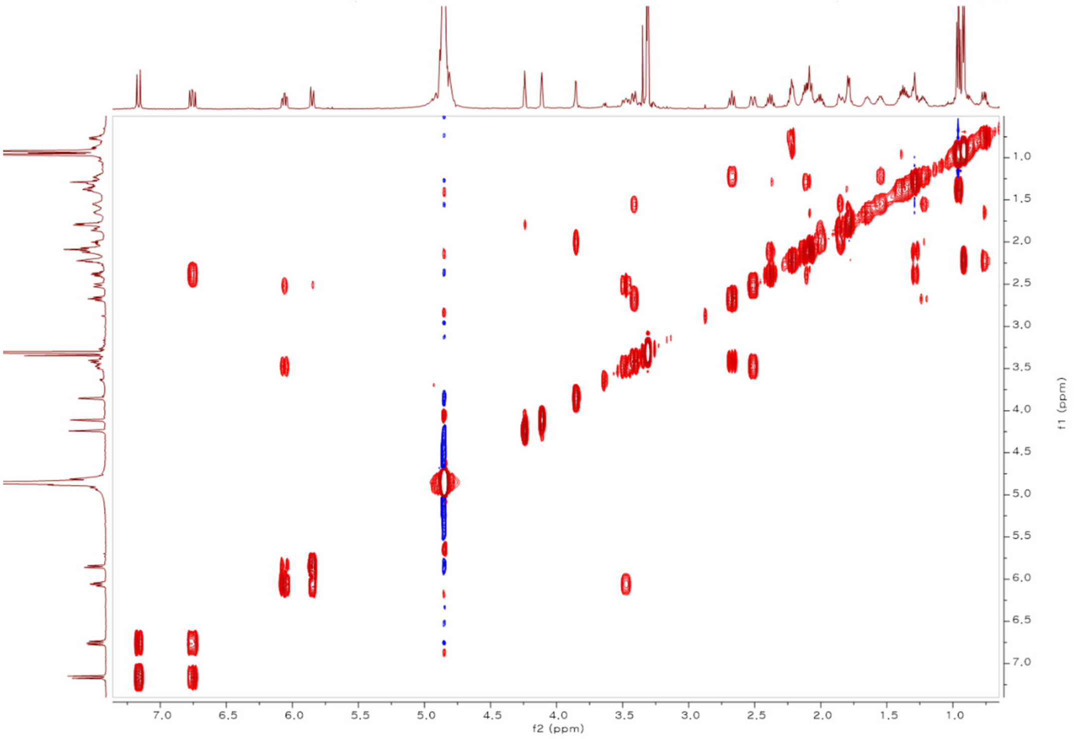

Figure S14. ^{1}H-^{1}H COSY spectrum of **3** (in CD_3OD).

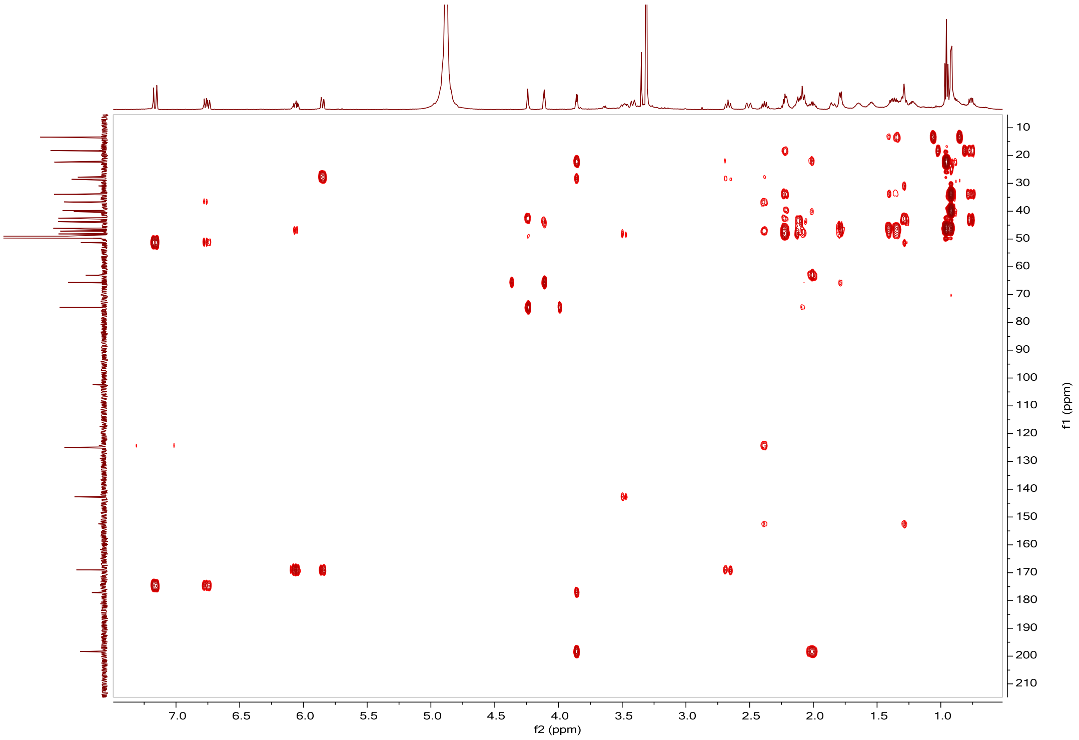

Figure S15. HMBC spectrum of **3** (in CD_3OD).

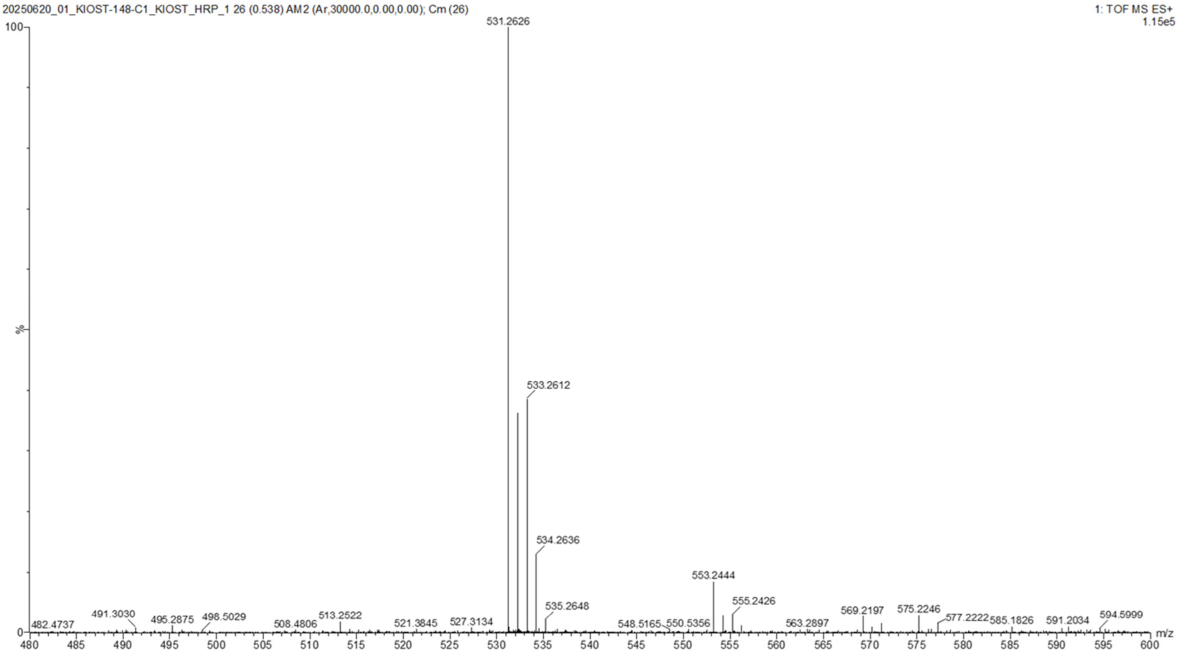

Elemental Composition Report

Single Mass Analysis
Tolerance = 5.0 PPM / DBE: min = -5.0, max = 50.0
Element prediction: Off
Number of isotope peaks used for i-FIT = 3

Monoisotopic Mass, Even Electron Ions
Elements Used:
C: 0-31 H: 0-180 N: 0-3 O: 0-6 Na: 0-1 Cl: 0-1

Minimum:				-5.0				
Maximum:		50.0	5.0	50.0				
Mass	Calc. Mass	mDa	PPM	DBE	i-FIT	Norm	Conf(%)	Formula
531.2626	531.2626	0.0	0.0	10.5	619.9	0.007	99.33	C29 H40 N2 O5 Cl
553.2444	553.2445	-0.1	-0.2	10.5	479.3	0.052	94.98	C29 H39 N2 O5 Na Cl

Figure S16. HR-ESIMS spectrum of **3**.

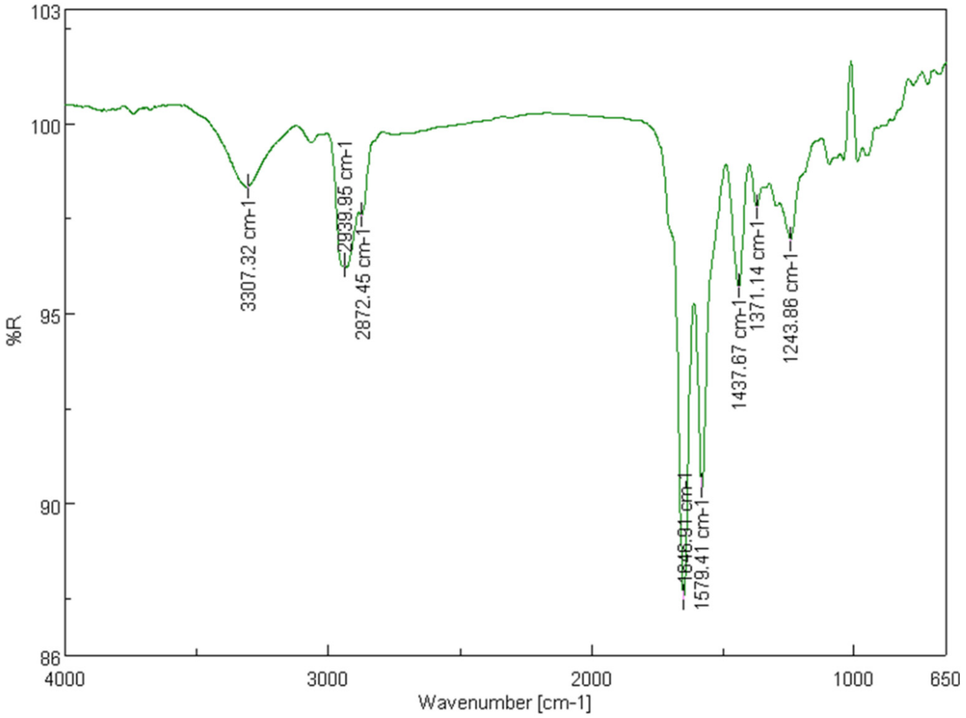

Figure S17. IR spectrum of **3**.

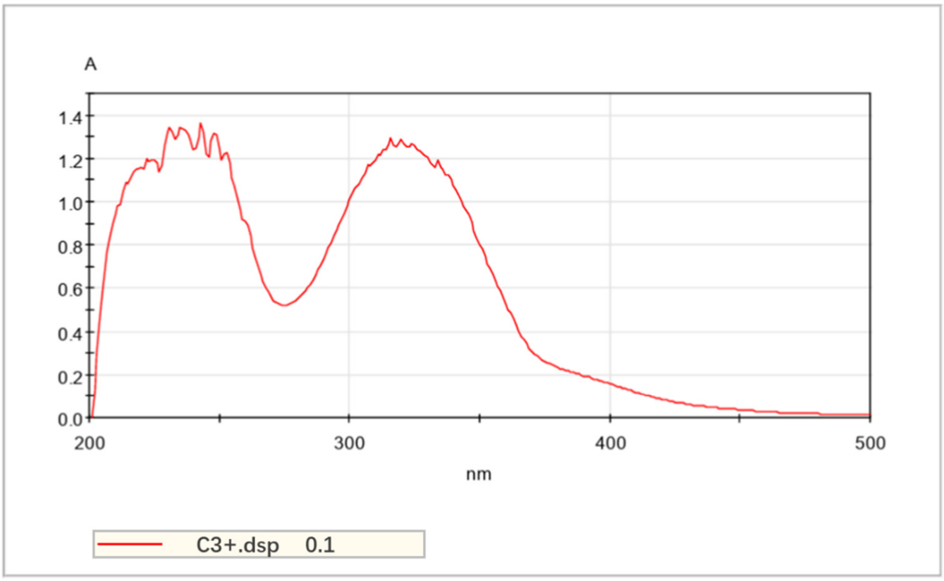


```
C3+.dsp       0.1

   Maxima       Threshold: 2 A
No peaks found

C3+.dsp    0.1
 236 nm   1.335 A
 320 nm   1.285 A
```

Figure S18. UV spectrum of **3**.

Table S3. ^{1}H and ^{13}C NMR data of **3** and Capsimycin D.

Position	**3**[a]		**Capsimycin D**[b]	
	δ_H, Mult (*J* in Hz)	δ_C, Mult	δ_H, Mult (*J* in Hz)	δ_C, Mult
1		169.0, C		167.5, C
2	5.85, d (11.5)	124.9, CH	5.84, dd (11.5, 1.3)	123.7, CH
3	6.06, ddd (11.5, 10.2, 4.0)	142.7, CH	6.06, ddd (11.5, 11.5, 3.4)	141.6, CH
4	2.51, m<br>3.47, m	27.7, CH2	2.53, dd (17.3, 3.0)<br>3.38, m	26.4, CH_2
5	2.13, m	47.1, CH	2.14, m	45.6, CH
6	2.09, m	48.9, CH	2.07, m	47.3, CH
7	4.11, t (3.0)	74.6, CH	4.13, bs t (2.6)	73.7, CH
8	4.24, t (3.0)	65.6, CH	4.23, bs t (2.9)	64.7, CH
9	1.70, m	48.1, CH	1.77, m	47.0, CH
10	1.79, m	46.2, CH	1.76, d (3.3)	44.7, CH
11	2.21, m	33.9, CH	2.21, m	32.6, CH
12	0.76, dd (10.2, 6.2)<br>2.23, m	39.8, CH2	0.75, m<br>2.19, d (7.6)	38.6, CH_2
13	1.65, m	42.5, CH	1.61, m	41.1, CH
14	2.08, m	43.8, CH	2.07, m	42.6, CH
15	1.30, m<br>2.11, m	36.6, CH2	1.29, m<br>2.13, dd (7.6, 4.8)	35.6, CH_2
16	2.38, m	51.3, CH	2.41, m	49.6, CH
17	6.76, dd (15.4, 10.2)	152.4, CH	6.83, dd (15.4, 10.3)	153.0, CH
18	7.16, d (15.4)	124.3, CH	7.13, d (15.4)	122.2, CH
19		175.1, C		173.6, C
20		102.4, C		100.8, C
21		177.1, C		175.6, C
22-NH				
23	3.85, dd (5.6, 2.1)	62.9, CH	3.88, dd (5.5, 2.1)	61.6, CH
24		198.4, C		197.1, C
25	1.89, m<br>2.00, m	28.5, CH2	1.82, m<br>2.05, m	27.5, CH_3
26	1.23, m<br>1.55, m	22.4, CH2	1.18, m<br>1.62, m	21.1, CH_2
27	2.67, ddd (13.6, 10.4, 2.8)<br>3.41, ddd (13.6, 5.5, 3.4)	40.2, CH2	2.65, br t (11.2)<br>3.55, ddd (11.2, 4.9, 3.0)	39.0, CH_2
28-NH				
29	1.37, m	22.3, CH2	1.35, m	21.2, CH_2
30	0.96, t (7.4)	13.3, CH3	0.94, t (7.4)	12.8, CH_3
31	0.92, d (6.6)	18.2, CH3	0.90, d (6.8)	17.7, CH_3

[a] Measured in CD_3OD; [b] Measured in 90% $CDCl_3/CD_3OD$ (*Scientific Reports,* **2017**, 7, 40689*)*.

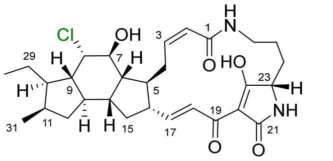

Figure S19. Structure of capsimycin D (**3**).

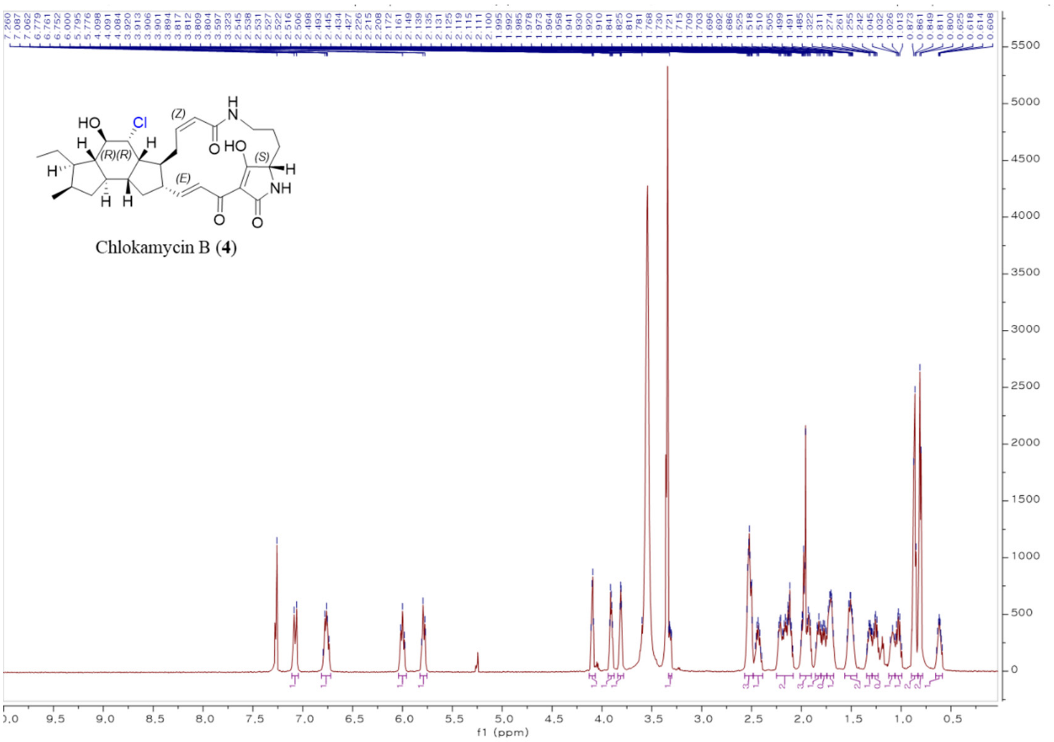

Figure S20. ^{1}H NMR spectrum of **4** (600 MHz, in 90% $CDCl_3$/CD_3OD).

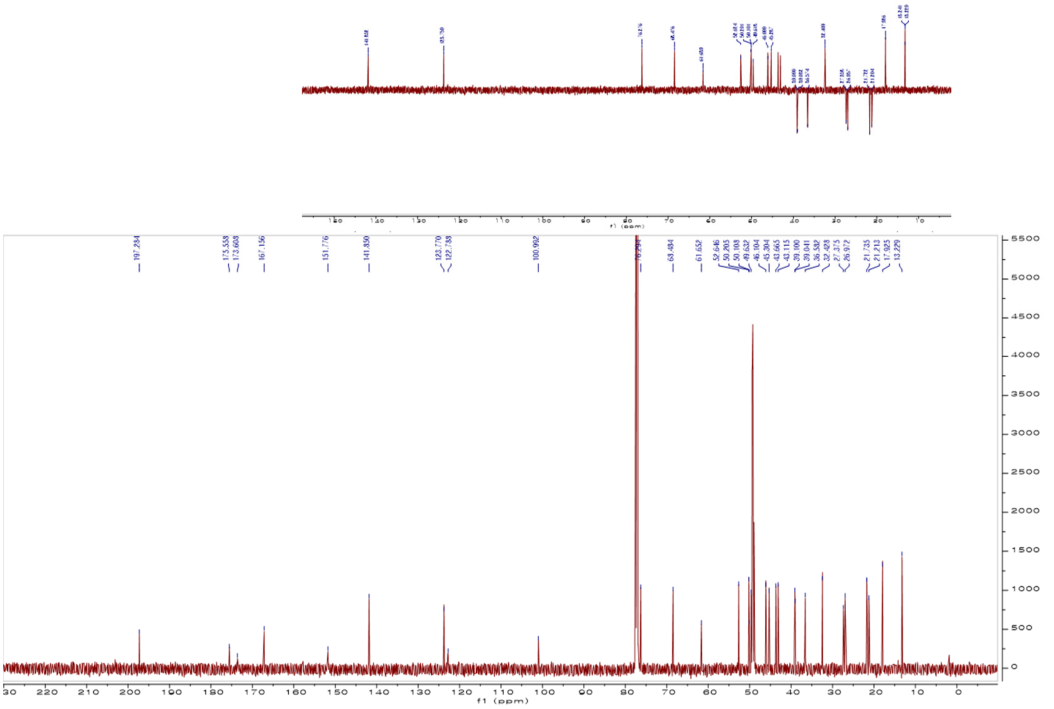

Figure S21. ^{13}C and DEPT NMR spectrum of **4** (150 MHz, in 90% $CDCl_3$/CD_3OD).

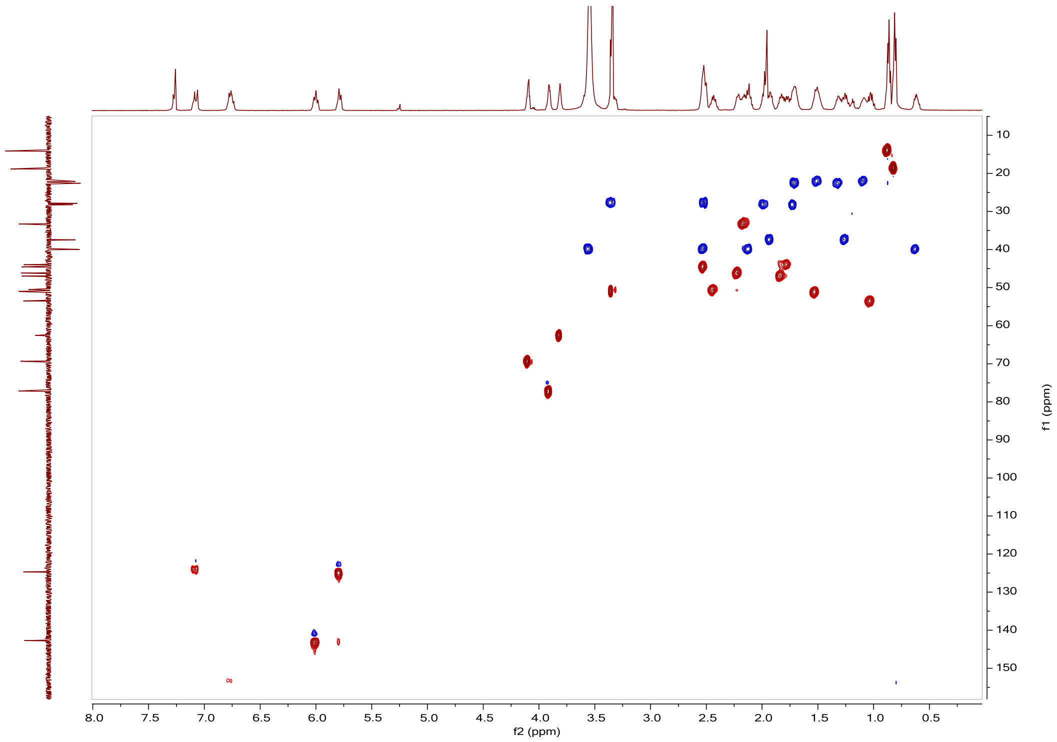

Figure S22. HSQC spectrum of **4** (in 90% $CDCl_3/CD_3OD$).

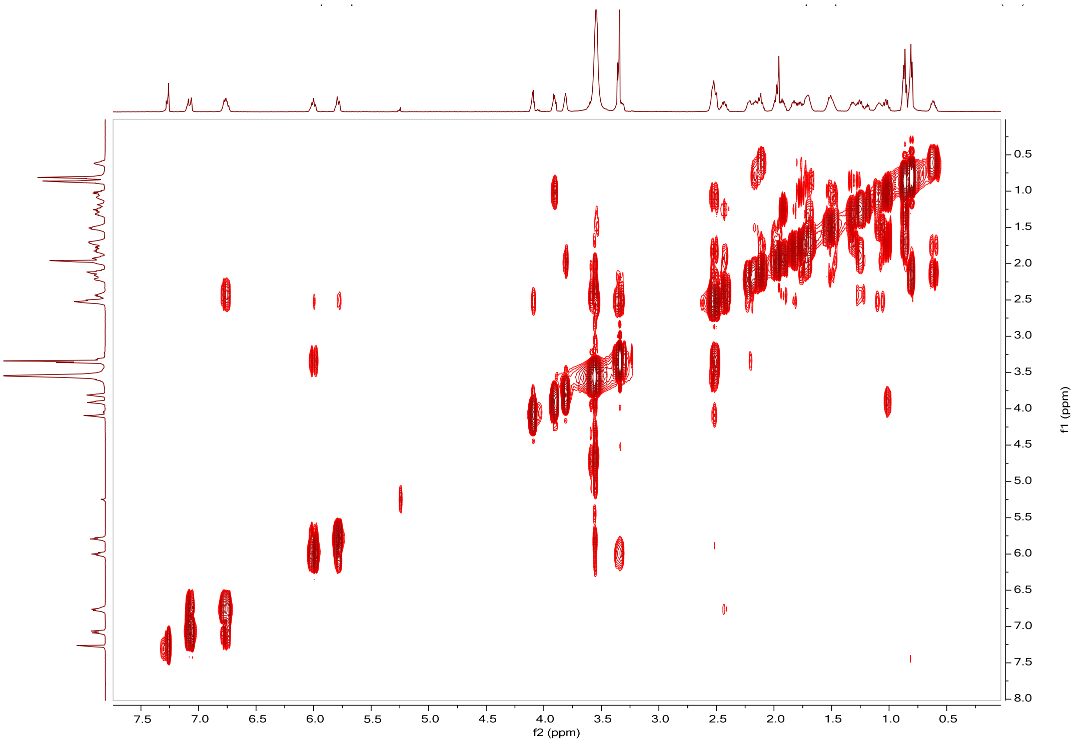

Figure S23. ^1H-^1H COSY spectrum of **4**.

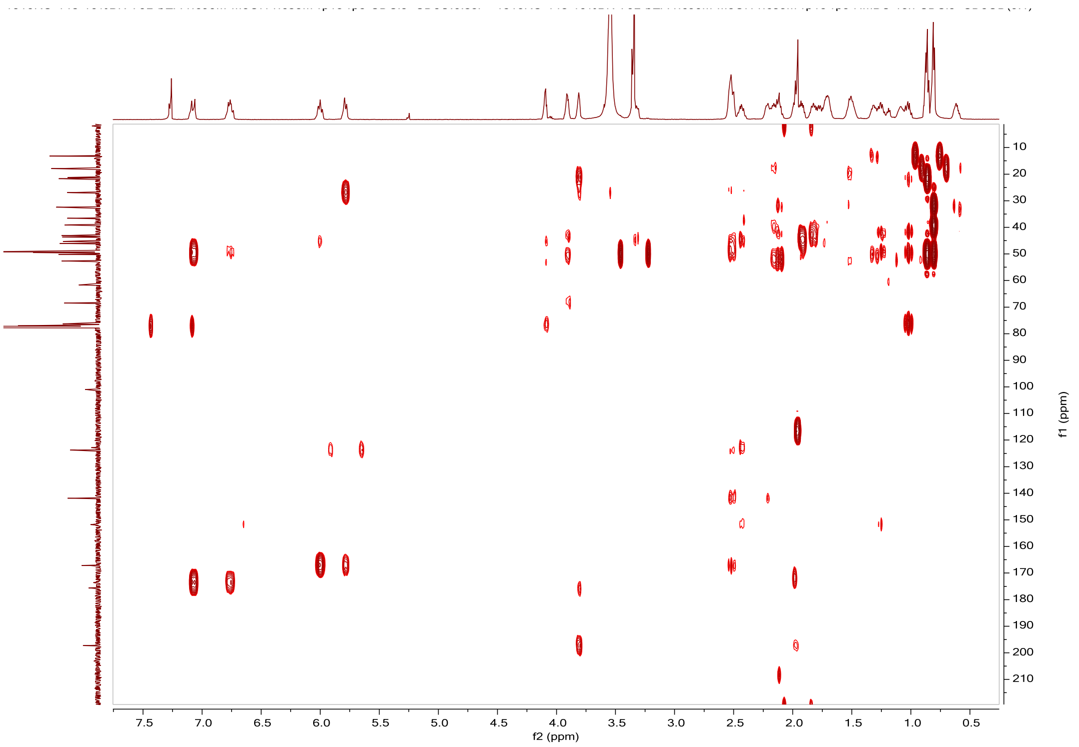

Figure S24. HMBC spectrum of **4**.

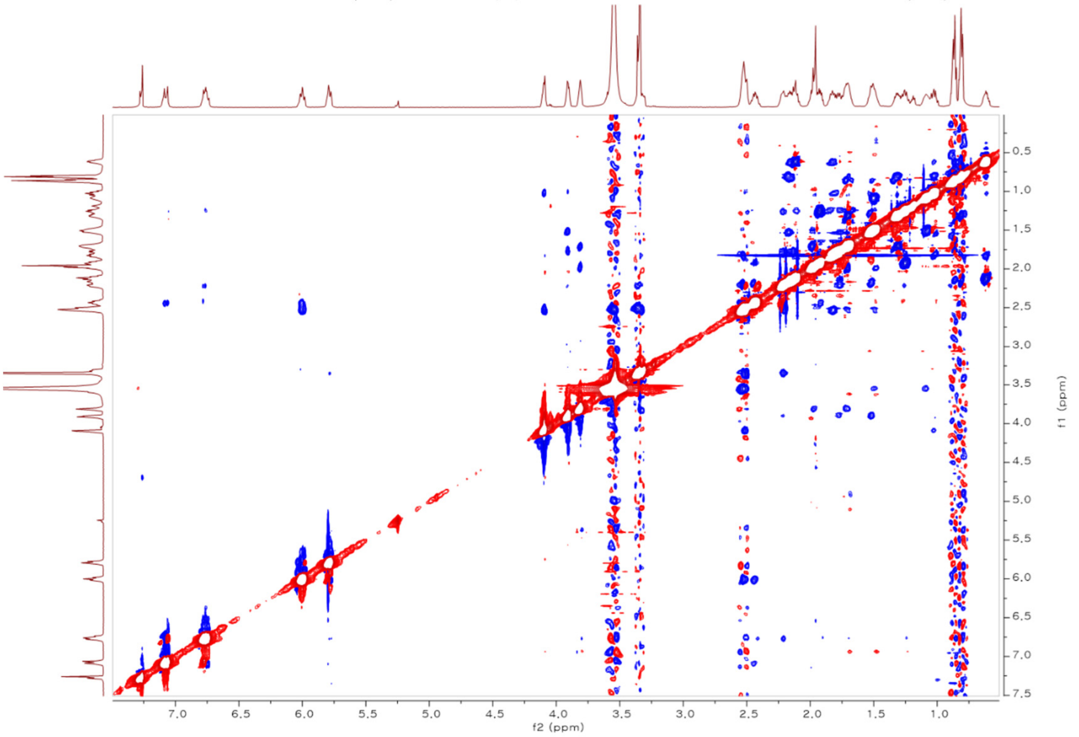

Figure S25. NOESY spectrum of **4**.

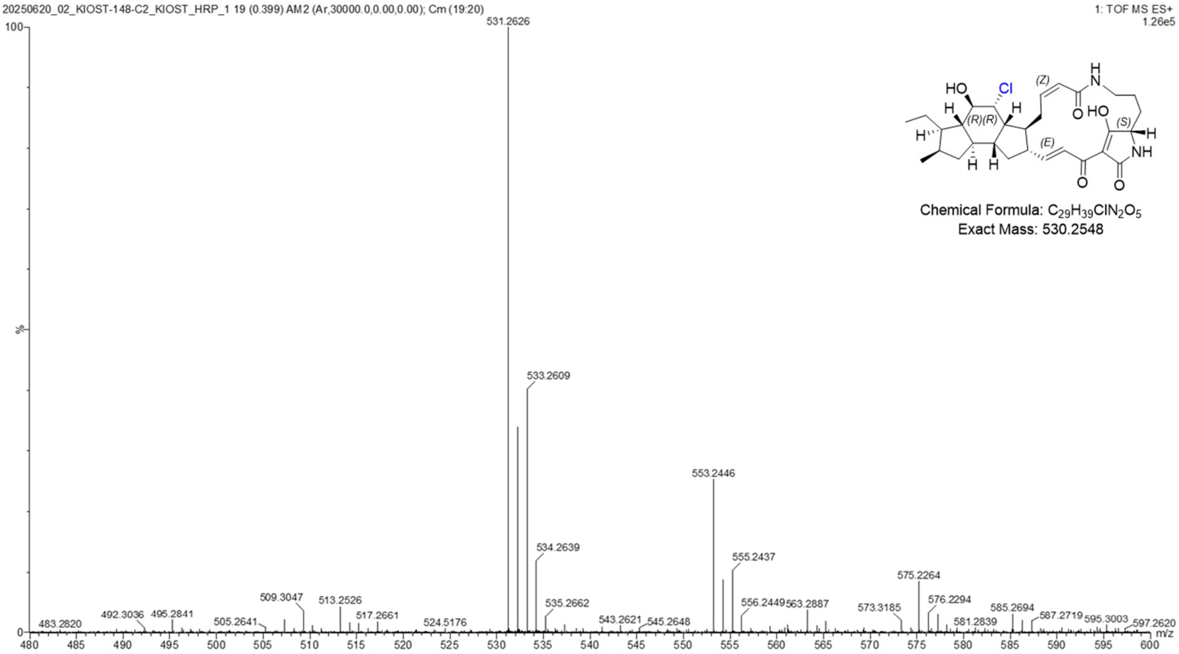

Elemental Composition Report

Single Mass Analysis
Tolerance = 5.0 PPM / DBE: min = -5.0, max = 50.0
Element prediction: Off
Number of isotope peaks used for i-FIT = 3
Monoisotopic Mass, Even Electron Ions
Elements Used:
C: 0-31 H: 0-180 N: 0-3 O: 0-6 Na: 0-1 Cl: 0-1

Minimum:				-5.0				
Maximum:		50.0	5.0	50.0				
Mass	Calc. Mass	mDa	PPM	DBE	i-FIT	Norm	Conf(%)	Formula
531.2626	531.2626	0.0	0.0	10.5	638.4	0.004	99.59	C29 H40 N2 O5 Cl
553.2446	553.2445	0.1	0.2	10.5	615.2	1.015	36.25	C29 H39 N2 O5 Na Cl

Figure S26. HR-ESIMS spectrum of **4**.

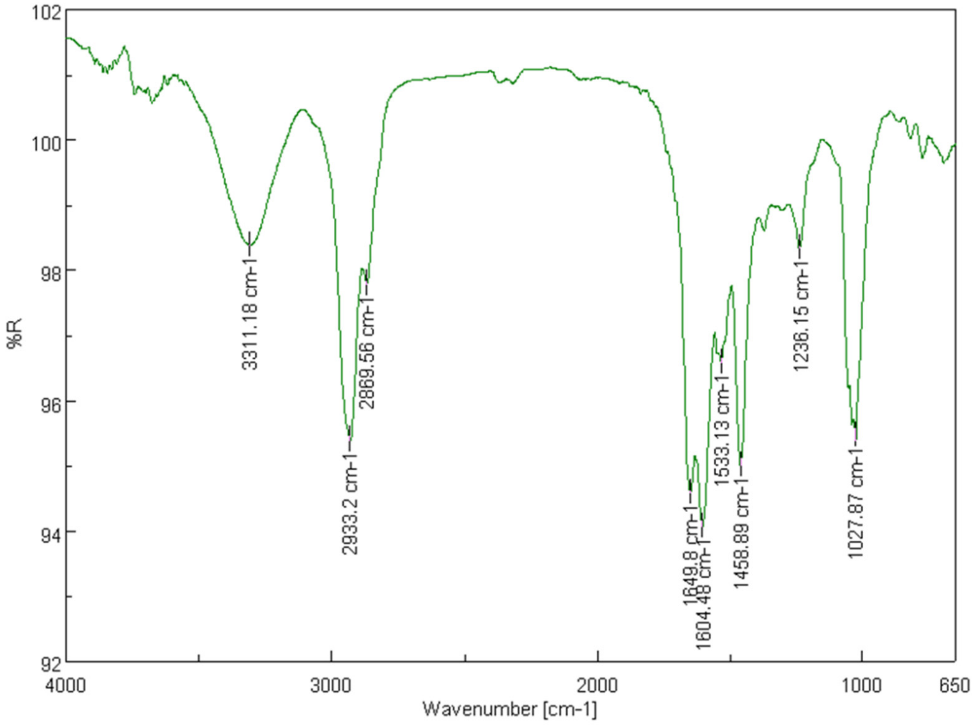

Figure S27. IR spectrum of **4**.

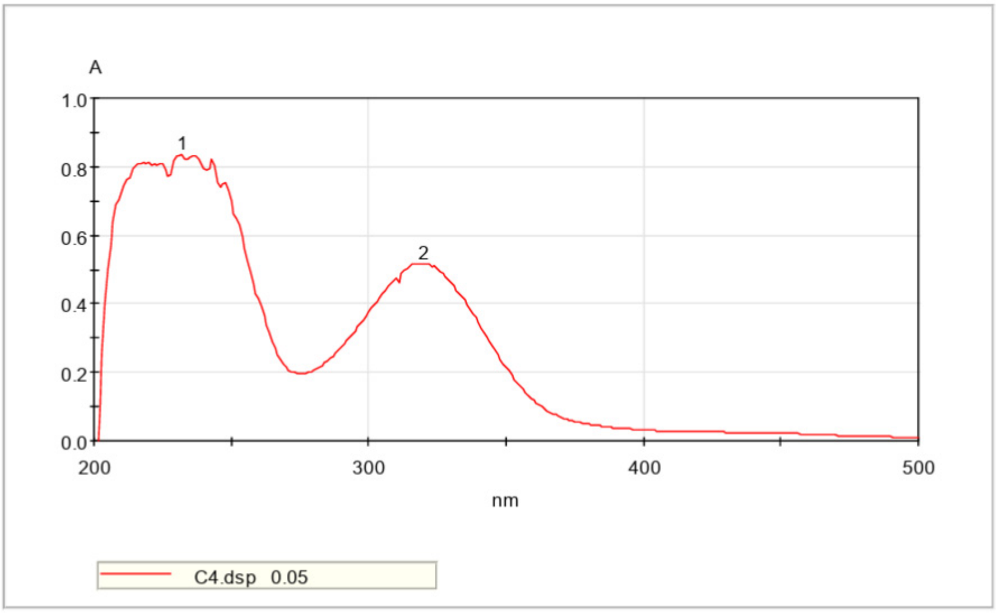


```
C4.dsp      0.05

   Maxima      Threshold: 0.1 A
  1  232 nm;    0.835 A      2  320 nm;    0.518 A
```

Figure S28. UV spectrum of **4**.

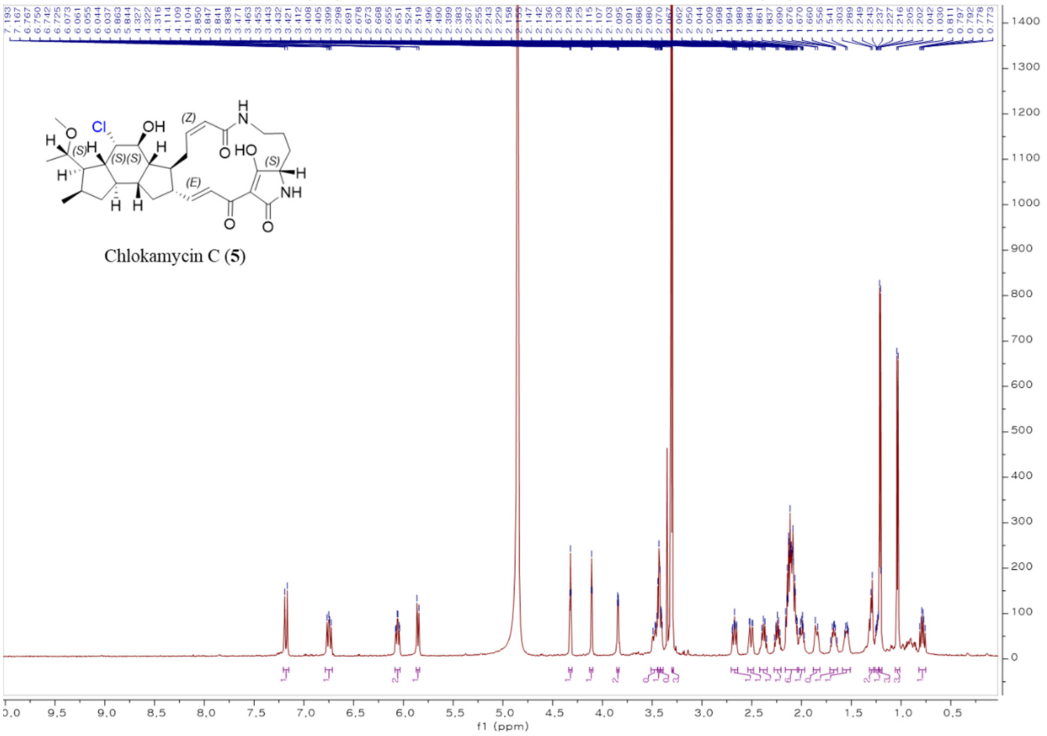

Figure S29. ^{1}H NMR spectrum of **5** (600 MHz, in CD_3OD).

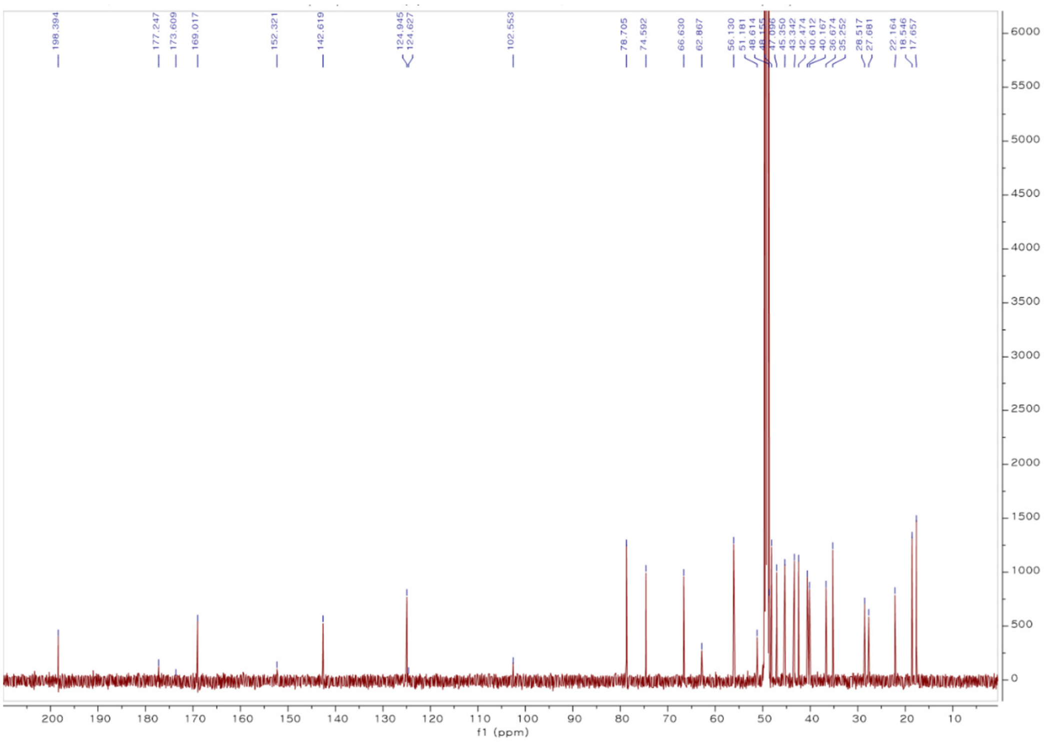

Figure S30. ^{13}C NMR spectrum of **5** (150 MHz, in CD_3OD).

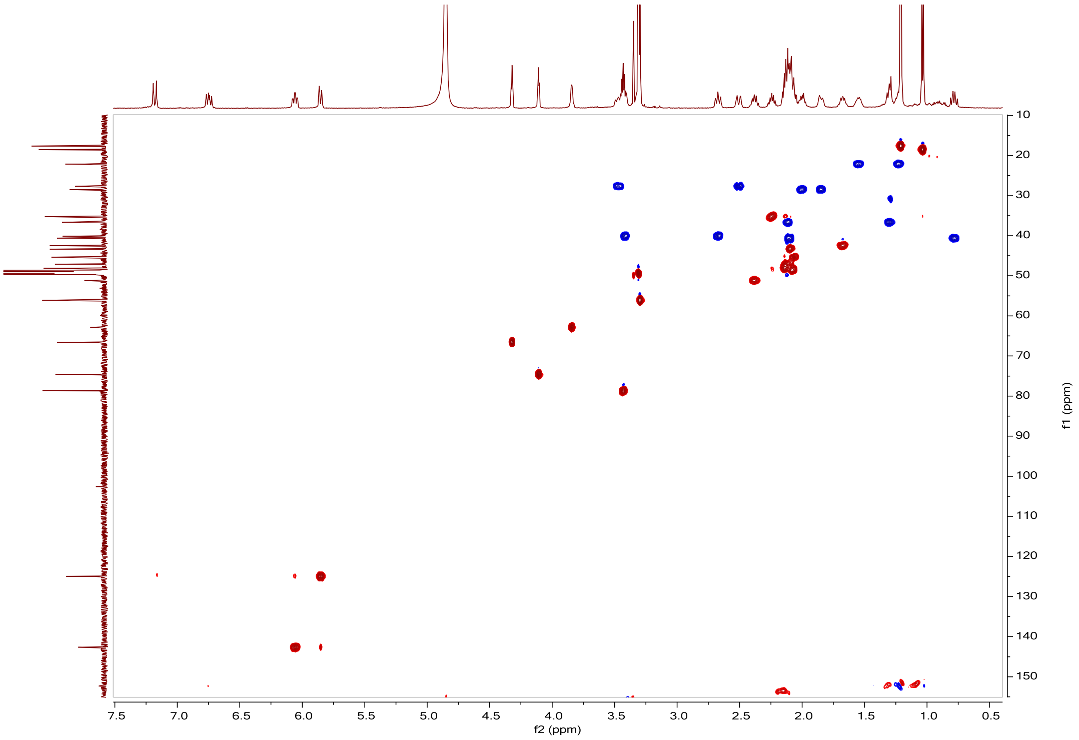

Figure S31. HSQC spectrum of **5** (in CD_3OD).

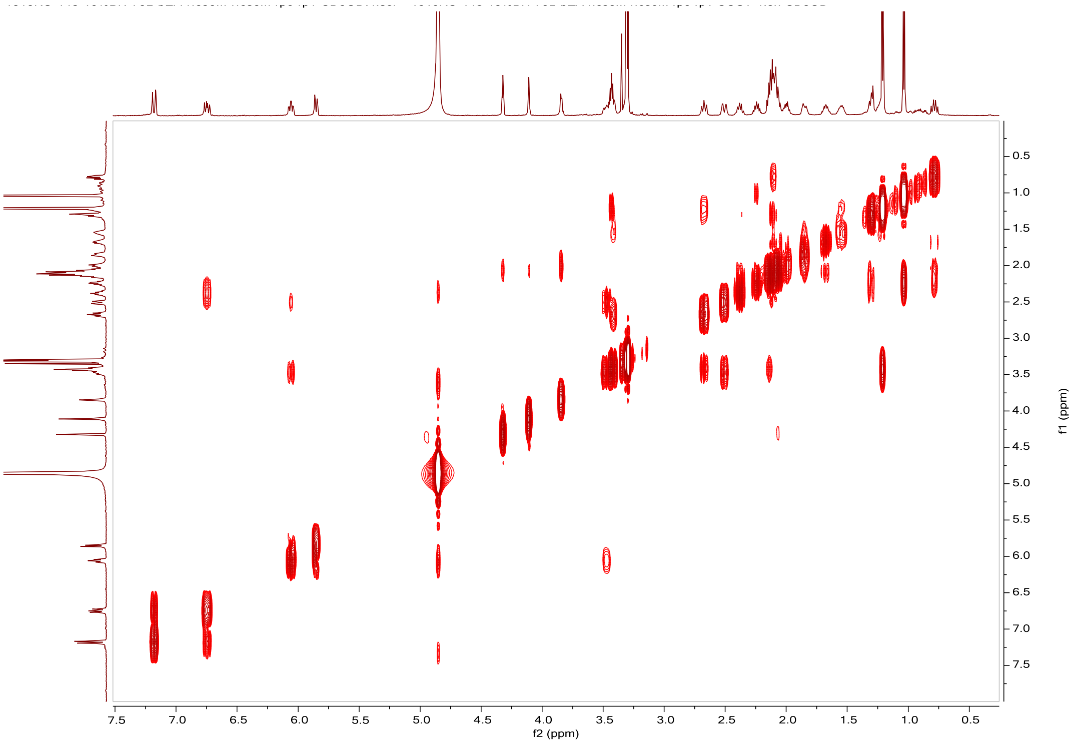

Figure S32. ^1H-^1H COSY spectrum of **5**.

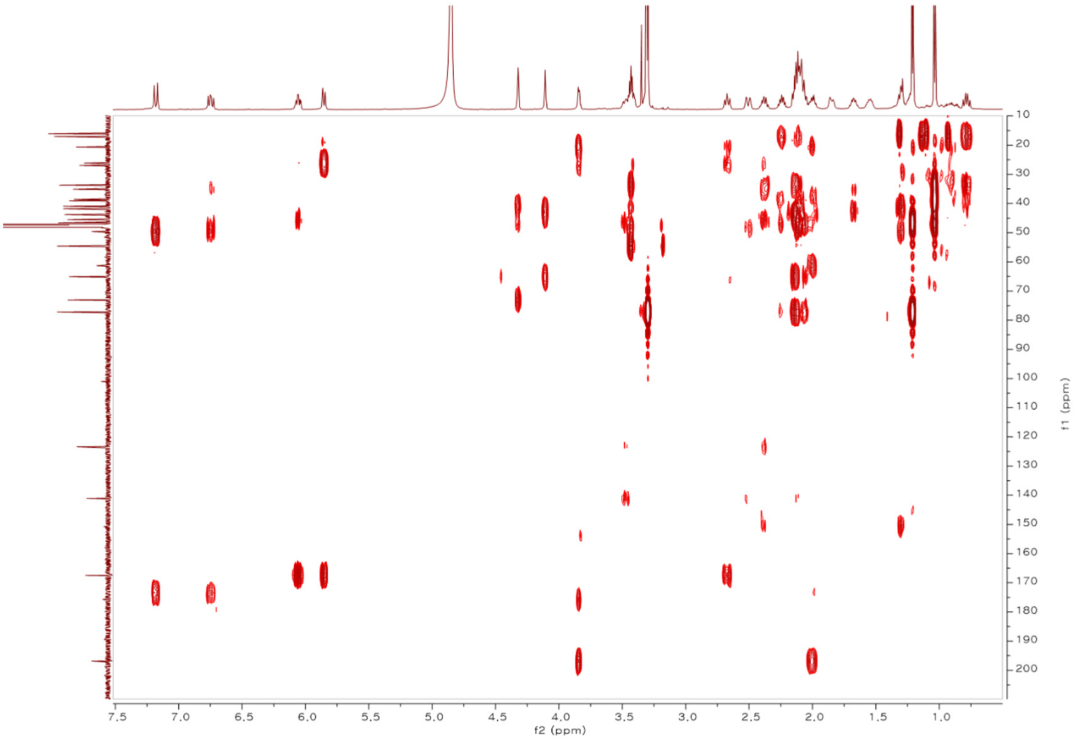

Figure S33. HMBC spectrum of **5**.

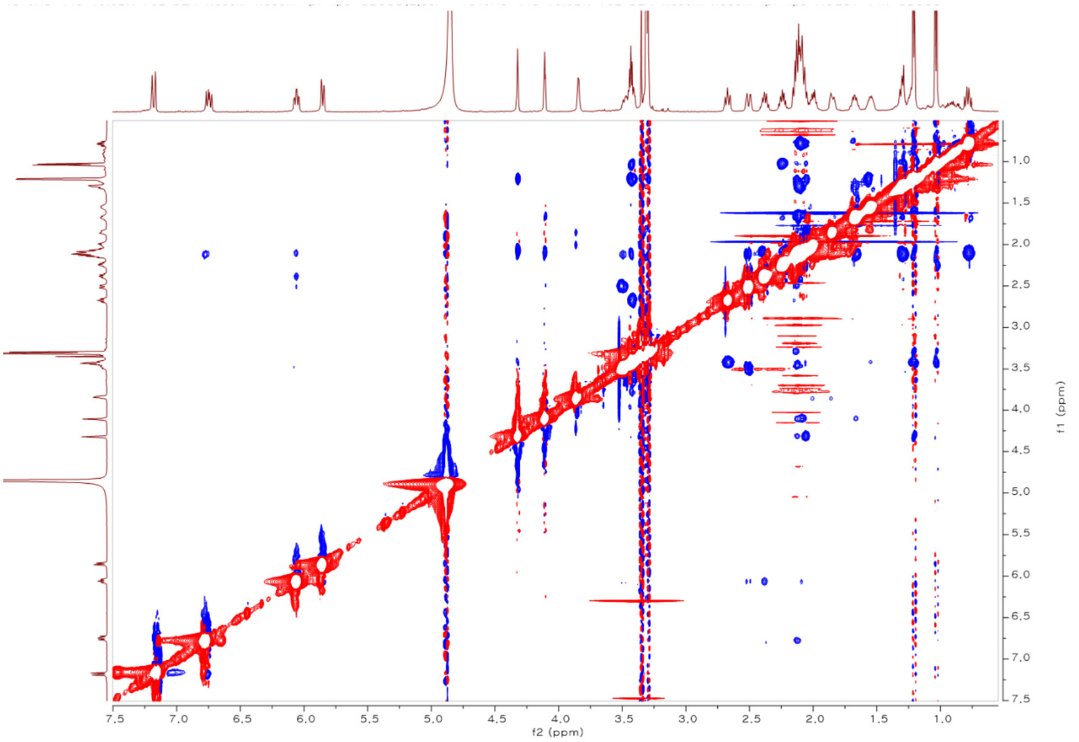

Figure S34. NOESY spectrum of **5**.

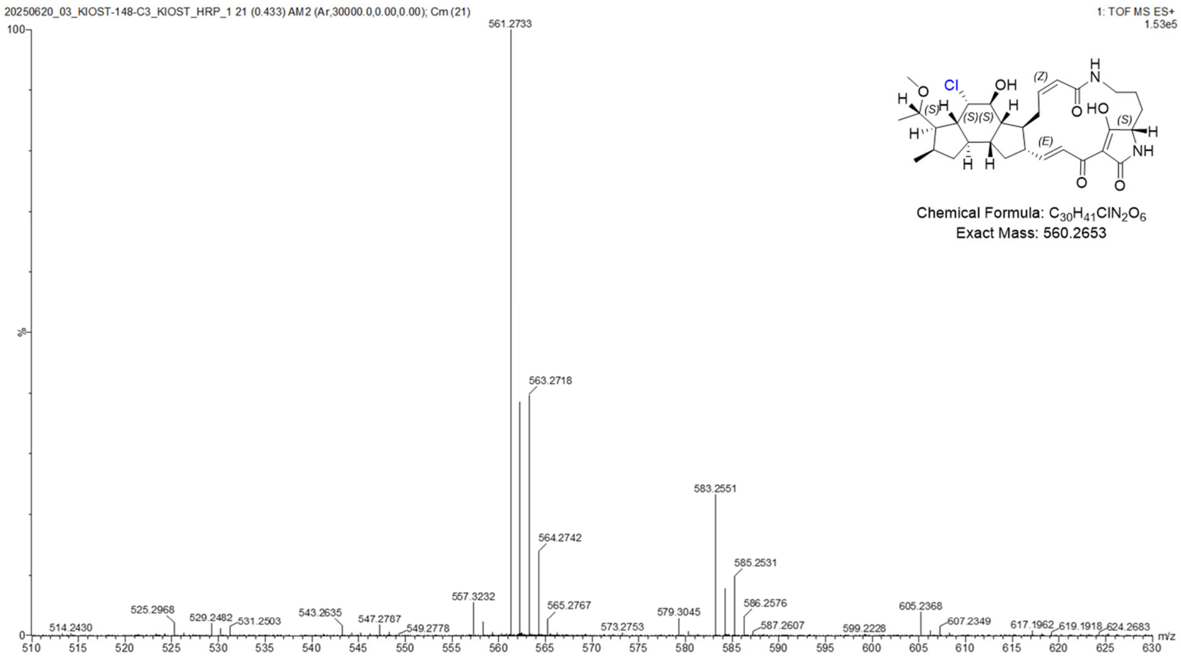

Elemental Composition Report

Single Mass Analysis
Tolerance = 5.0 PPM / DBE: min = -5.0, max = 50.0
Element prediction: Off
Number of isotope peaks used for i-FIT = 3
Monoisotopic Mass, Even Electron Ions
Elements Used:
C: 0-31 H: 0-180 N: 0-3 O: 0-6 Na: 0-1 Cl: 0-1

Minimum:				-5.0				
Maximum:		50.0	5.0	50.0				
Mass	Calc. Mass	mDa	PPM	DBE	i-FIT	Norm	Conf(%)	Formula
561.2733	561.2731	0.2	0.4	10.5	638.3	0.012	98.80	C30 H42 N2 O6 Cl
583.2551	583.2551	0.0	0.0	10.5	521.4	n/a	n/a	C30 H41 N2 O6 Na Cl

Figure S35. HR-ESIMS spectrum of **5**.

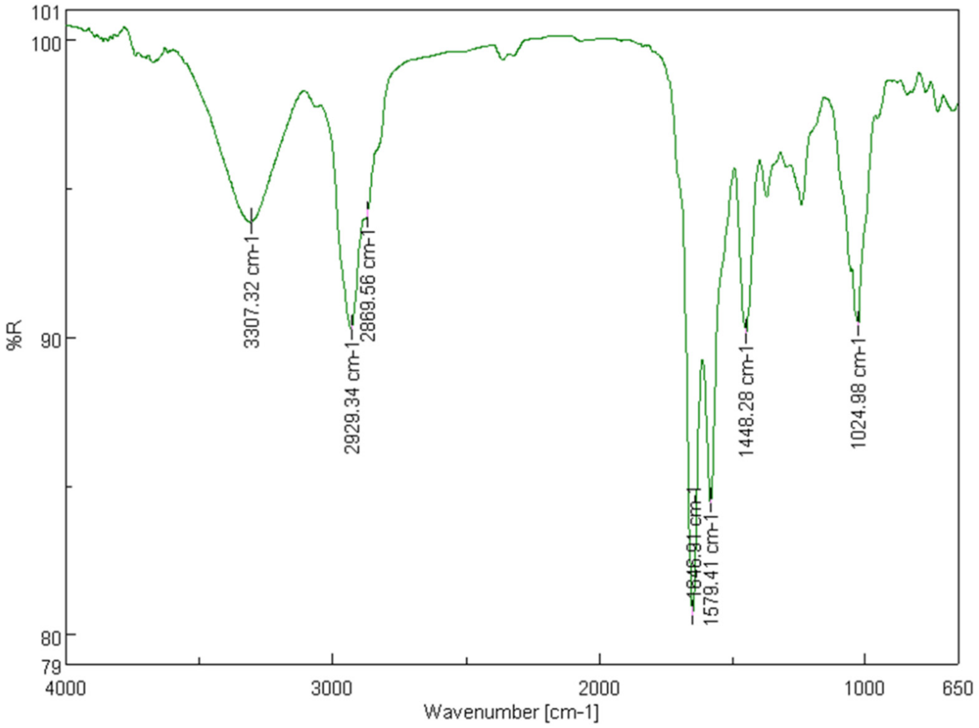

Figure S36. IR spectrum of **5**.

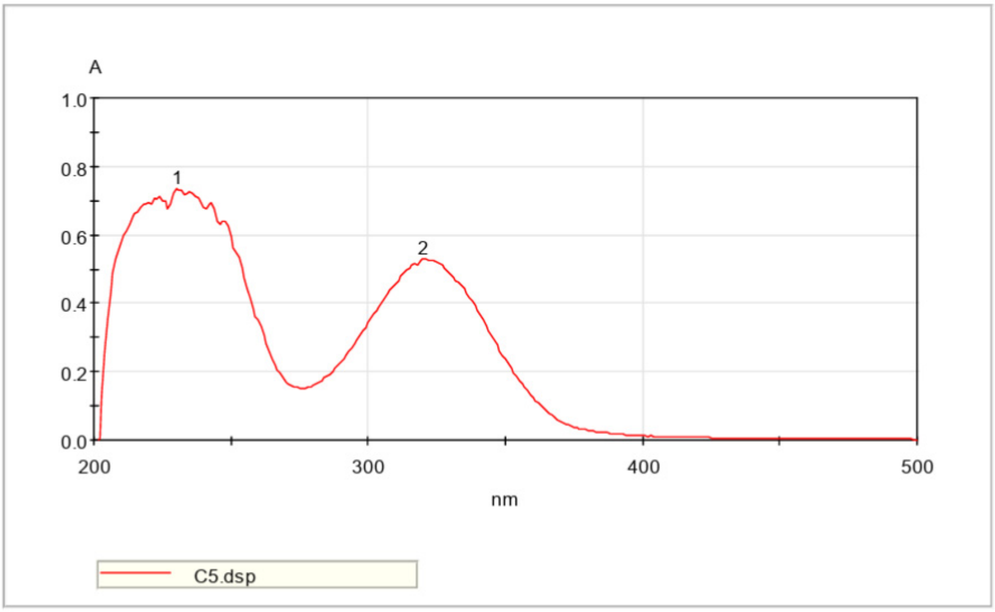

C5.dsp

Maxima Threshold: 0.1 A
1 230 nm; 0.736 A 2 320 nm; 0.528 A

Figure S37. UV spectrum of **5**.

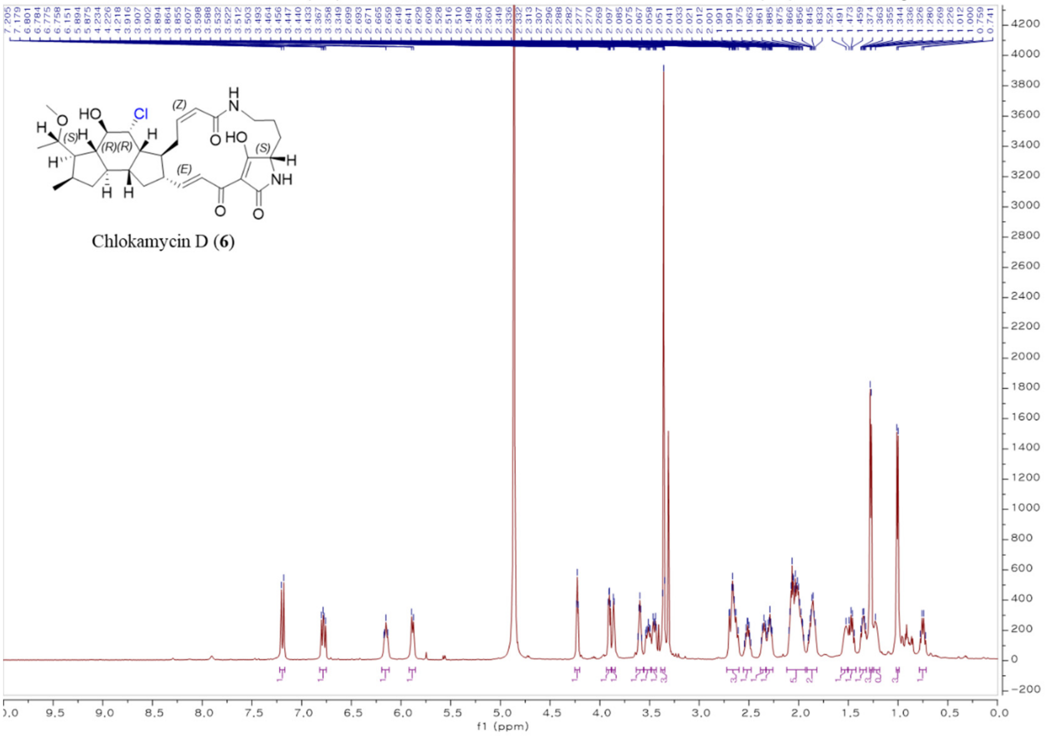

Figure S38. ^{1}H NMR spectrum of **6** (600 MHz, in CD_3OD).

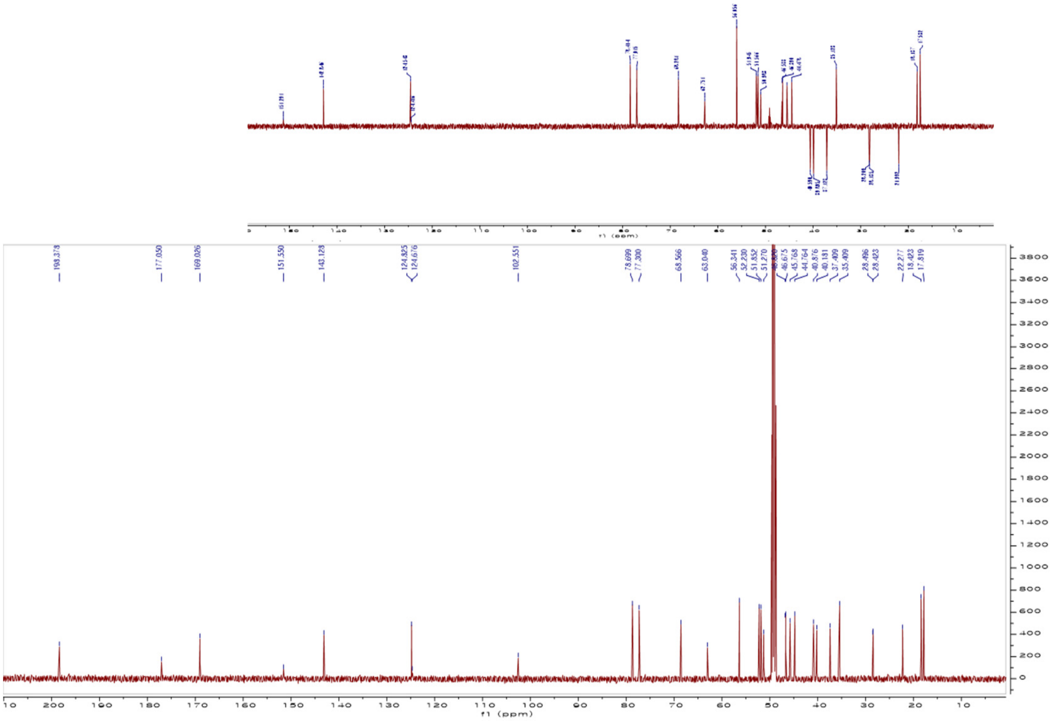

Figure S39. ^{13}C NMR (150 MHz, in CD_3OD) and DEPT spectra of **6.**

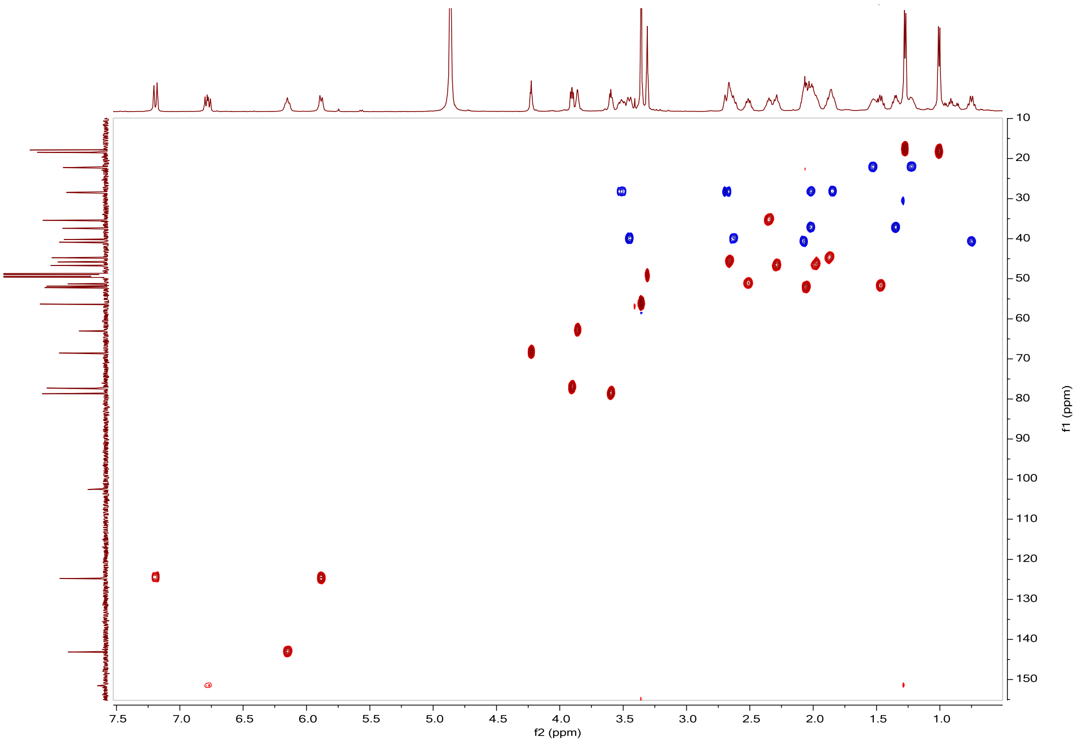

Figure S40. HSQC spectrum of **6** (in CD_3OD).

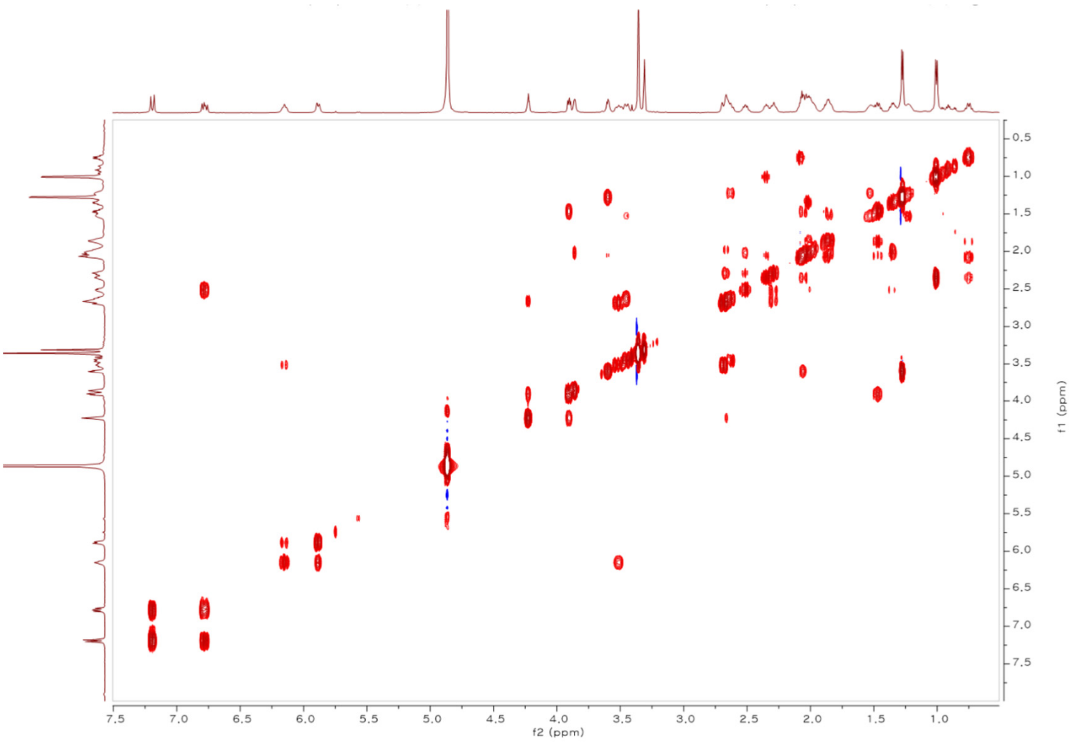

Figure S41. ^1H-^1H COSY spectrum of **6**.

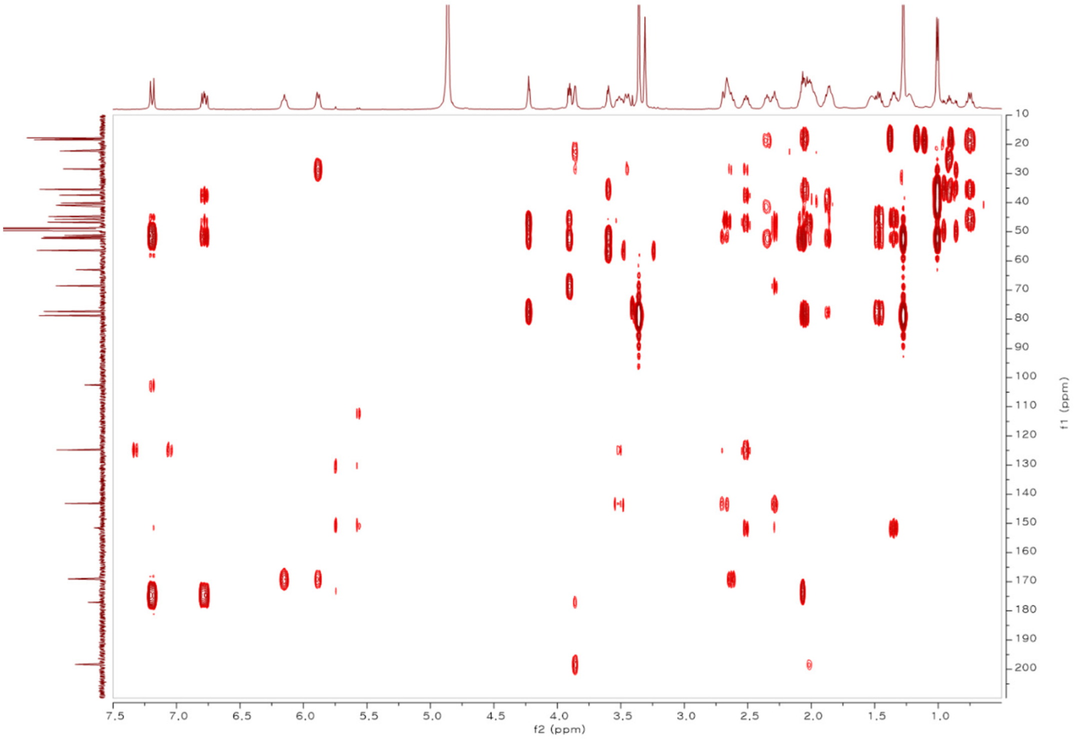

Figure S42. HMBC spectrum of **6**.

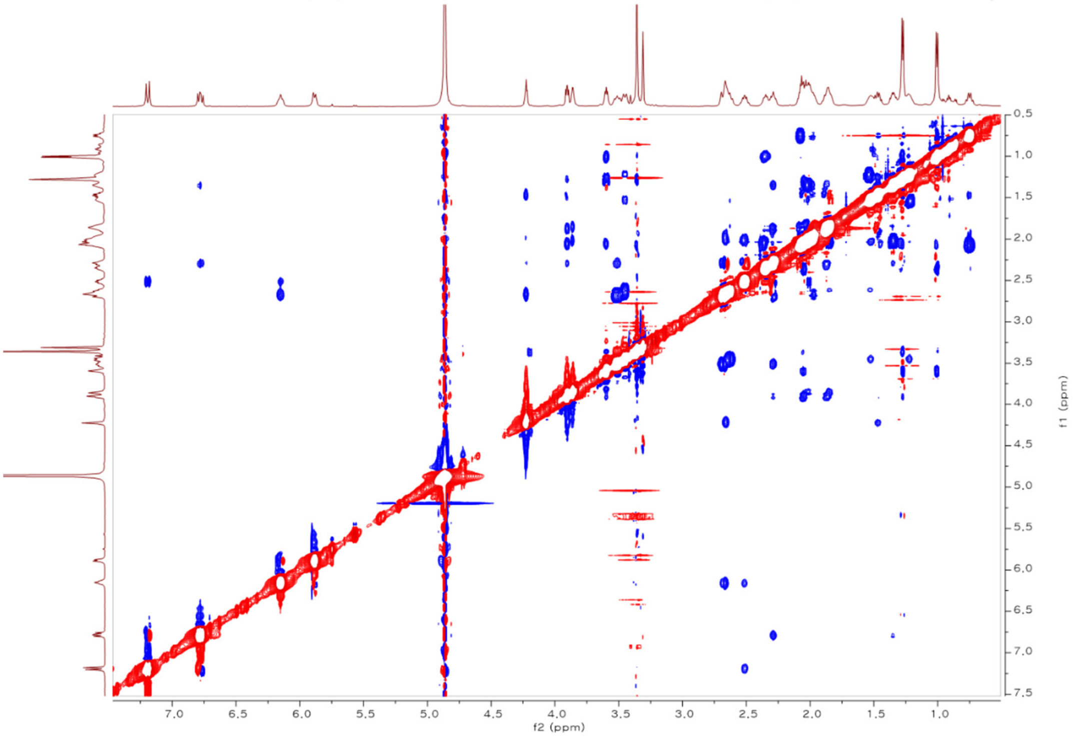

Figure S43. NOESY spectrum of **6**.

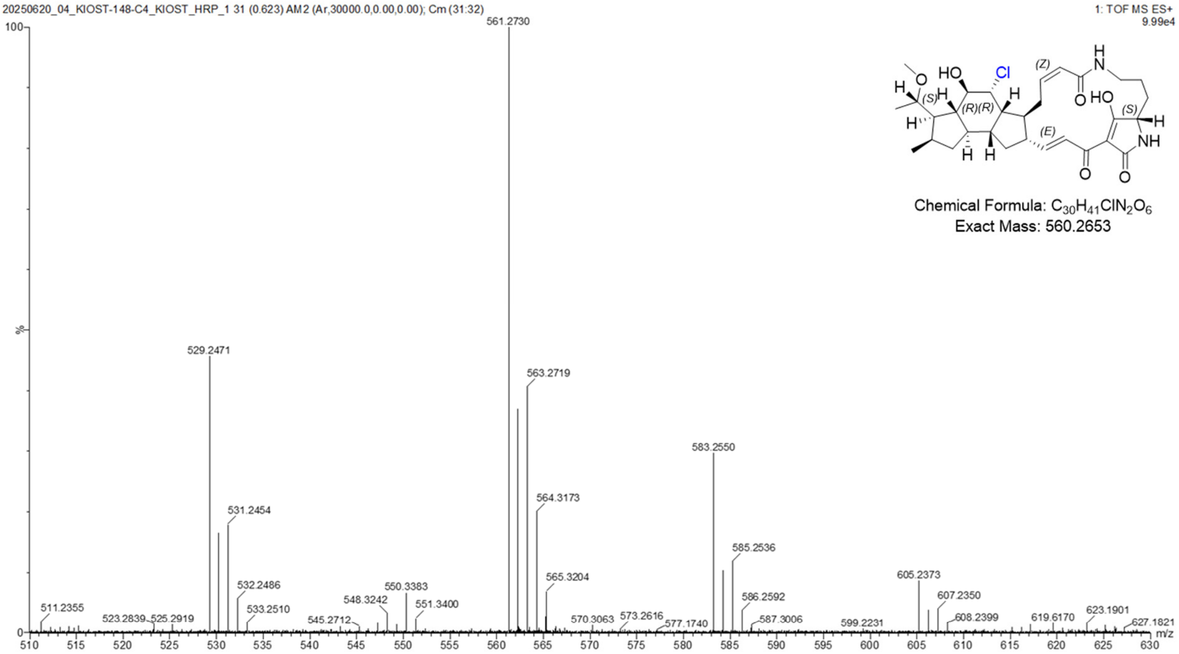

Elemental Composition Report

Single Mass Analysis
Tolerance = 5.0 PPM / DBE: min = -5.0, max = 50.0
Element prediction: Off
Number of isotope peaks used for i-FIT = 3
Monoisotopic Mass, Even Electron Ions
Elements Used:
C: 0-31 H: 0-180 N: 0-3 O: 0-6 Na: 0-1 Cl: 0-1

Minimum:				-5.0				
Maximum:		50.0	5.0	50.0				
Mass	Calc. Mass	mDa	PPM	DBE	-FIT	Norm	Conf(%)	Formula
561.2730	561.2731	-0.1	-0.2	10.5	614.1	0.007	99.26	C30 H42 N2 O6 Cl
583.2550	583.2551	-0.1	-0.2	10.5	600.1	n/a	n/a	C30 H41 N2 O6 Na Cl

Figure S44. HR-ESIMS spectrum of **6**.

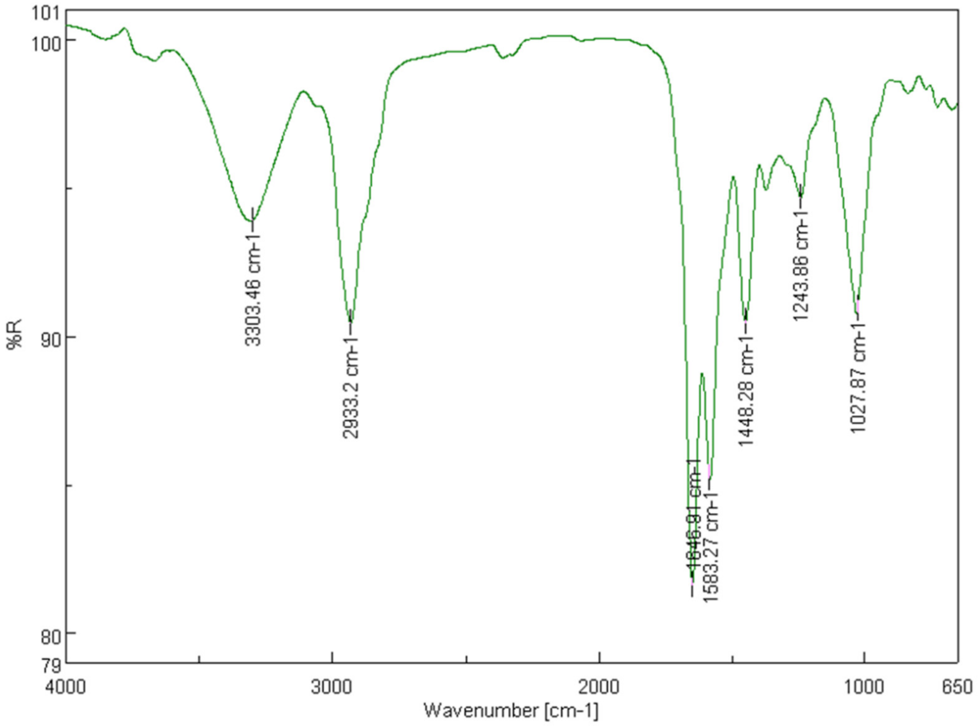

Figure S45. IR spectrum of **6**.

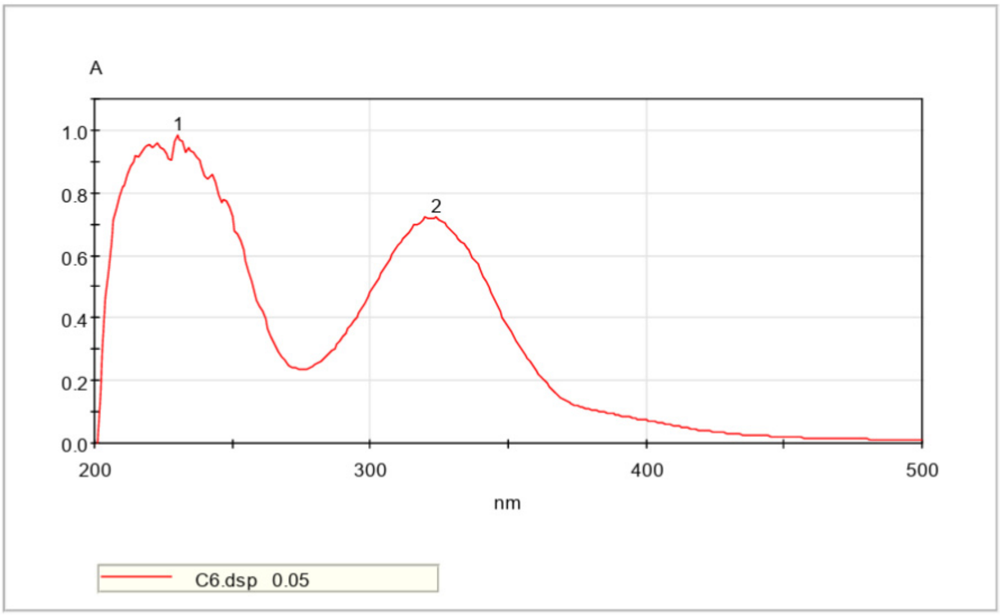

C6.dsp 0.05

Maxima **Threshold: 0.1 A**

1 230 nm; 0.985 A 2 324 nm; 0.722 A

Figure S46. UV spectrum of **6**.

Table S4. Conformers and Boltzmann distributions of the optimized **4**.

Comp. **4**	Conf. No.	BOLTZMANN POPULATION, %	TOTAL GIBBS FREE ENERGY, KCAL/MOL
7R,8R,23S-**4A**	#1	26.4544	420.4390
	#2	24.9486	420.4730
	#3	12.3645	420.2065
	#4	6.5609	420.9988
	#5	5.0007	422.0303
7S,8S,23R-**4B**	#1	26.4544	420.4390
	#2	24.9486	420.4730
	#3	12.3645	420.2065
	#4	6.5609	420.9988
	#5	5.0007	422.0303
7R,8R,23R-**4C**	#1	22.9596	420.6119
	#2	16.5898	419.7961
	#3	9.9497	420.6850
	#4	7.8640	421.2599
	#5	7.7431	420.6359
	#6	7.3208	420.7922
	#7	5.3202	420.1781

Table S5. The Cartesian coordinates of the lowest-energy conformers of (5*S*,6*R*,7*R*,8*R*,9*S*,10*R*,11*R*,13*R*,14*R*,16*S*,23*S*)-**4A**.

4A	Conf. #1			Conf. #2			Conf. #3			Conf. #4			Conf. #5		
	Coordinates (Angstroms)			Coordinates (Angstroms)			Coordinates (Angstroms)			Coordinates (Angstroms)			Coordinates (Angstroms)		
	X	Y	Z	X	Y	Z	X	Y	Z	X	Y	Z	X	Y	Z
C	-1.80895	-2.10763	-0.42086	1.38373	-0.12853	-2.23893	-1.76713	-1.686	-1.21328	-1.80664	-2.10361	-0.43884	-1.36099	-1.25676	-0.94271
C	-1.1011	-2.00066	-1.55589	1.00929	-1.3985	-2.46853	-1.11276	-1.03723	-2.19007	-1.10383	-1.99289	-1.57668	-0.71189	-1.06106	-2.1007
C	-0.30759	0.91206	-2.48347	-0.03631	-3.29989	-0.04694	-0.18576	1.98826	-1.99644	-0.31757	0.92581	-2.48973	-0.26685	1.97878	-1.07615
Cl	1.38164	1.00476	1.54961	-2.82002	-3.31101	1.73001	1.33563	0.33387	1.80654	1.39815	0.99565	1.54212	2.48775	3.65143	-0.13892
O	4.1526	2.55287	-0.72196	-3.95104	-0.56106	2.284	4.19665	2.63066	0.48324	4.27253	2.47098	-0.7842	4.593	2.04431	1.33288
C	1.37462	-2.04288	-0.73166	-1.54077	-1.1413	-2.8969	1.35829	-1.4806	-1.48896	1.37539	-2.03839	-0.76451	1.76002	-0.87937	-2.723
C	0.30064	-1.47533	-1.67725	-0.36947	-1.95883	-2.2745	0.30893	-0.56271	-2.13623	0.29692	-1.46556	-1.70169	0.43984	-0.13672	-2.33773
C	0.37684	0.06146	-1.39059	-0.72018	-2.10143	-0.74706	0.42303	0.75153	-1.28894	0.37273	0.06925	-1.40511	0.80135	0.85051	-1.19907
C	2.55308	-1.07686	-0.938	-2.62905	-1.01085	-1.80538	2.55989	-0.54415	-1.28492	2.55241	-1.06896	-0.96474	2.89921	-0.10907	-2.00913
C	1.8869	0.33011	-1.15182	-2.27198	-2.13965	-0.78948	1.92812	0.84398	-0.91232	1.88356	0.33764	-1.17191	2.24698	1.25156	-1.60537
C	2.22345	1.35937	-0.06855	-3.12357	-1.97264	0.47333	2.2384	1.31424	0.51246	2.22624	1.36159	-0.08657	3.18335	2.00567	-0.65714
C	3.74208	1.51266	0.18828	-3.06459	-0.60051	1.16439	3.75004	1.30932	0.84762	3.75292	1.51568	0.15785	3.66975	1.24755	0.58893
C	6.02853	-1.59371	0.60178	-3.54183	2.64217	-0.91675	6.00076	-1.71647	-0.05746	6.00957	-1.60122	0.62603	5.09505	-2.34649	0.25662
C	5.70859	-0.0895	0.90017	-3.42271	1.9417	0.48872	5.68665	-0.47638	0.84595	5.69656	-0.09007	0.9048	4.84773	-1.06472	1.13721
C	4.51985	0.20451	-0.03429	-3.50546	0.43971	0.13272	4.52223	0.20698	0.10391	4.52079	0.20437	-0.04541	4.37068	-0.02118	0.10122
C	3.64221	-1.04933	0.13096	-2.61413	0.36021	-1.11834	3.6298	-0.98402	-0.28854	3.638	-1.04599	0.10825	3.41464	-0.85228	-0.77052
C	4.66494	-2.20348	0.13299	-3.08039	1.56533	-1.949	4.64065	-2.05084	-0.75662	4.65922	-2.19967	0.10749	4.20579	-2.14712	-1.01117
C	7.11995	-1.82169	-0.45426	-4.94124	3.164	-1.27206	7.10826	-1.48926	-1.09687	7.13944	-1.8521	-0.38296	6.55635	-2.59359	-0.14387
C	6.87862	0.89984	0.83029	-4.36834	2.45728	1.58199	6.86664	0.42687	1.22527	6.8722	0.89179	0.83857	5.99571	-0.65382	2.06924
C	7.93648	0.68843	1.92084	-4.04059	3.88294	2.04568	7.89626	-0.24907	2.13961	7.89492	0.70783	1.96678	6.22492	-1.64	3.22233
C	-3.26663	-2.28191	-0.47848	2.80591	0.18652	-2.07586	-3.23228	-1.78897	-1.25023	-3.26442	-2.27854	-0.48965	-2.60701	-2.03221	-0.87851
C	-4.0788	-1.69868	0.57911	3.17273	1.3066	-1.22083	-3.95599	-1.84624	0.01243	-4.07109	-1.70238	0.57605	-3.45795	-1.86099	0.28954
C	-0.46744	2.37654	-2.15449	1.45387	-3.40597	-0.25924	-0.48904	3.11462	-1.04443	-0.47715	2.38831	-2.15205	-0.66163	2.26367	0.34456
C	-1.42854	2.96338	-1.42159	2.45805	-2.78401	0.37768	-1.65892	3.38733	-0.44681	-1.43521	2.97049	-1.41144	-1.88685	2.36398	0.87924
C	-2.51484	2.24191	-0.70686	2.33498	-1.7742	1.46169	-2.93505	2.63378	-0.6318	-2.51764	2.24443	-0.69539	-3.19279	2.27692	0.16509
N	-3.48014	3.02096	-0.1418	3.5226	-1.22151	1.84425	-3.78602	2.71093	0.4298	-3.47888	3.02013	-0.1189	-4.23741	1.93772	0.97028
O	-2.54724	1.01338	-0.59813	1.26335	-1.44893	1.98381	-3.21156	1.98976	-1.64846	-2.55013	1.01523	-0.59511	-3.33927	2.51833	-1.0392
O	-3.8543	-2.77357	-1.47015	3.72141	-0.56152	-2.48959	-3.89622	-1.68394	-2.30547	-3.85737	-2.76436	-1.4811	-3.04256	-2.71062	-1.83554
C	-3.70563	-1.11412	1.88864	2.38917	2.4556	-0.70284	-3.49717	-2.10292	1.40066	-3.69105	-1.12654	1.88741	-3.19551	-1.24837	1.6136
N	-4.80801	-0.42548	2.32531	3.20615	3.09262	0.19482	-4.58163	-1.86472	2.20315	-4.7913	-0.44129	2.33473	-4.42591	-1.02359	2.18164
C	-5.96197	-0.51671	1.43534	4.47542	2.41554	0.43024	-5.7523	-1.36067	1.49524	-5.95018	-0.52738	1.45065	-5.54018	-1.53859	1.3902
C	-5.39246	-1.39221	0.35105	4.38831	1.32177	-0.59439	-5.27098	-1.47213	0.07516	-5.38615	-1.39515	0.35732	-4.80173	-2.10468	0.20885
O	-6.10131	-1.71985	-0.70718	5.38249	0.4826	-0.78313	-6.05495	-1.18246	-0.93938	-6.10068	-1.71627	-0.69908	-5.43852	-2.66435	-0.79456
O	-2.64783	-1.21928	2.50573	1.25399	2.81183	-1.01055	-2.39347	-2.47884	1.78843	-2.6299	-1.23538	2.4981	-2.11602	-1.00221	2.14353
C	-6.51485	0.83673	0.92027	4.67358	1.90506	1.88329	-6.16876	0.07081	1.92921	-6.50697	0.82921	0.94817	-6.60316	-0.49101	0.98242
C	-4.48491	2.44225	0.75159	3.63352	-0.2636	2.94047	-5.0079	1.91902	0.53767	-4.47825	2.43597	0.77702	-5.63042	1.93369	0.53772
C	-5.59569	1.67548	0.01139	3.45516	1.21309	2.54316	-5.05661	1.13523	1.85897	-5.59409	1.67449	0.03901	-6.19605	0.61355	-0.01899
H	3.17089	-1.00183	1.11832	-1.58819	0.56413	-0.78398	3.13883	-1.34767	0.62049	3.16256	-1.00566	1.09391	2.55024	-1.10264	-0.14044
H	4.87906	0.2227	-1.0739	-4.54124	0.19807	-0.14879	4.90372	0.65704	-0.8244	4.89632	0.22504	-1.07843	5.23064	0.29434	-0.50804
H	3.03523	-1.34484	-1.8865	-3.6274	-1.1809	-2.22212	3.04976	-0.41591	-2.25818	3.03752	-1.33099	-1.9134	3.74073	0.0651	-2.68778
H	2.31633	0.77739	-2.05676	-2.55875	-3.09917	-1.23584	2.40124	1.61408	-1.53338	2.30875	0.78852	-2.07712	2.16174	1.86588	-2.50956
H	6.35466	-2.07789	1.52658	-2.85445	3.49244	-0.94107	6.30787	-2.55139	0.57857	6.29193	-2.08334	1.56635	4.75072	-3.22281	0.81312
H	5.31168	-0.04592	1.92463	-2.39703	2.11549	0.84372	5.26551	-0.86533	1.78421	5.28753	-0.03449	1.92417	3.97873	-1.28094	1.77503
H	-0.16914	0.22777	-0.4622	-0.38634	-1.19504	-0.23771	-0.15215	0.57135	-0.37807	-0.16899	0.22908	-0.473	0.84315	0.30035	-0.25433
H	0.63692	-1.64506	-2.70727	-0.38728	-2.96704	-2.69931	0.63383	-0.33526	-3.1586	0.62853	-1.62797	-2.73438	0.15584	0.47545	-3.20478
H	-6.78316	-1.04588	1.93442	5.318	3.07047	0.18093	-6.61124	-2.02536	1.64031	-6.76816	-1.06055	1.95074	-6.0479	-2.34471	1.93263
H	-1.41003	4.04465	-1.31689	3.47138	-3.01605	0.06194	-1.69096	4.20021	0.27369	-1.4172	4.05123	-1.3012	-1.9551	2.51024	1.95366
H	0.27645	3.04299	-2.58569	1.75919	-4.09803	-1.04122	0.34961	3.75241	-0.77225	0.26381	3.0578	-2.5837	0.16751	2.3664	1.04012
H	-1.62814	-2.19852	-2.48572	1.79829	-2.10513	-2.70466	-1.69774	-0.72134	-3.04967	-1.63445	-2.18886	-2.50487	-1.07415	-1.59372	-2.97783
H	-1.39217	-1.85963	0.54785	0.68197	0.64687	-1.95424	-1.28689	-1.98746	-0.2898	-1.38546	-1.85802	0.52856	-1.07518	-0.75192	-0.02906
H	-6.82567	1.41792	1.79497	4.95937	2.77525	2.47844	-6.52627	-0.01659	2.95825	-6.81245	1.40433	1.82877	-6.99874	-0.04548	1.90071
H	-7.43152	0.60286	0.37139	5.54887	1.2482	1.87717	-7.03606	0.36619	1.33044	-7.42701	0.59859	0.40358	-7.42736	-1.06116	0.54435
H	-5.13472	1.03656	-0.7443	3.20996	1.75618	3.4601	-5.19973	1.84336	2.68066	-5.13843	1.04096	-0.72441	-5.51459	0.224	-0.78039
H	-6.21795	2.39735	-0.52693	2.56883	1.29445	1.91006	-4.08346	0.66651	2.03066	-6.22008	2.40015	-0.48981	-7.10755	0.89339	-0.55698
H	-3.97248	1.77606	1.44569	2.86322	-0.52108	3.66921	-5.88535	2.57057	0.45877	-3.96169	1.76513	1.46356	-6.23774	2.26599	1.38432
H	-4.91572	3.26051	1.33224	4.6047	-0.41618	3.41749	-5.02095	1.25588	-0.32569	-4.90496	3.25066	1.36569	-5.71565	2.6908	-0.24364
H	1.82561	2.33681	-0.33183	-4.16385	-2.15943	0.20479	1.86383	2.32195	0.67623	1.81037	2.33564	-0.3379	4.06703	2.30706	-1.22064
H	3.88174	1.86723	1.21445	-2.03989	-0.38991	1.4967	3.8603	1.1852	1.92963	3.89994	1.89499	1.17349	2.81712	0.97984	1.22645
H	-1.29607	0.48263	-2.65671	-0.49423	-4.22492	-0.41289	-1.10725	1.68839	-2.49122	-1.30654	0.49666	-2.66085	-1.1556	1.71612	-1.6485
H	0.2652	0.82458	-3.41321	-0.23924	-3.22654	1.01853	0.51311	2.33927	-2.76392	0.2511	0.84406	-3.4225	0.12587	2.89713	-1.52593
H	4.77076	-2.60635	-0.88031	-3.92807	1.26779	-2.57733	4.76183	-1.9954	-1.84397	4.79154	-2.57329	-0.91419	4.84322	-2.02482	-1.89464
H	4.3451	-3.03165	0.76995	-2.3041	1.94201	-2.61868	4.30001	-3.06359	-0.52837	4.32458	-3.04603	0.7123	3.55946	-3.00683	-1.2015
H	1.63022	-3.07945	-0.96501	-1.92386	-1.66553	-3.77467	1.59493	-2.34532	-2.11376	1.63197	-3.07261	-1.00702	1.89196	-0.88517	-3.80631
H	1.02616	-2.0079	0.30573	-1.20891	-0.15965	-3.23813	1.00147	-1.85251	-0.52336	1.02988	-2.01273	0.27417	1.73636	-1.92127	-2.39773
H	7.34838	0.8597	-0.1586	-5.40608	2.4073	1.23464	7.35958	0.80374	0.32244	7.37016	0.81557	-0.13399	6.91993	-0.53086	1.49419
H	6.48501	1.91851	0.94062	-4.30483	1.78391	2.43846	6.4798	1.308	1.75321	6.47531	1.9114	0.89024	5.76674	0.32892	2.48492
H	6.87129	-1.33486	-1.40281	-5.68976	2.36598	-1.23453	6.88002	-0.64143	-1.75086	6.93664	-1.36721	-1.34339	6.97722	-1.73605	-0.67865
H	7.2242	-2.89229	-0.65546	-4.9391	3.56085	-2.29218	7.20761	-2.3739	-1.73327	7.23574	-2.9255	-0.57382	6.6143	-3.45641	-0.81487
H	8.09649	-1.45177	-0.13244	-5.27179	3.96643	-0.60865	8.08198	-1.30559	-0.63618	8.10743	-1.49433	-0.02366	7.19817	-2.80231	0.71502
H	7.49055	0.77643	2.91656	-3.02549	3.93453	2.45322	7.42739	-0.58739	3.06901	7.42134	0.83457	2.94579	5.32666	-1.72711	3.84263
H	8.40033	-0.29906	1.85297	-4.10393	4.6102	1.2316	8.35357	-1.12075	1.6643	8.35144	-0.28564	1.94886	6.47696	-2.64328	2.86804
H	8.73257	1.43434	1.84544	-4.72902	4.20716	2.83168	8.69994	0.44337	2.40496	8.70119	1.44313	1.89073	7.04119	-1.30392	3.86849
H	5.11502	2.60447	-0.71351	-3.7063	-1.27995	2.88093	5.15875	2.65525	0.53652	3.97641	3.35211	-0.5262	4.15368	2.87422	1.55933
H	-3.41585	4.02421	-0.22088	4.36662	-1.53546	1.39017	-3.4968	3.24867	1.23441	-3.4147	4.02384	-0.19186	-4.03784	1.69129	1.92738
H	-4.87478	-0.08263	3.27198	2.90138	3.90084	0.71594	-4.532	-1.94296	3.20773	-4.85327	-0.10567	3.28432	-4.5117	-0.85596	3.17376
H	-5.47292	-2.24802	-1.27894	5.06609	-0.1281	-1.50798	-5.48693	-1.3229	-1.74983	-5.47522	-2.24054	-1.27764	-4.72775	-2.89321	-1.45726

Table S6. The Cartesian coordinates of the lowest-energy conformers of (5*R*,6*S*,7*S*,8*S*,9*R*,10*S*,11*S*,13*S*,14*S*,16*R*,23*R*)-**4B.**

4B	Conf. #1			Conf. #2			Conf. #3			Conf. #4			Conf. #5		
	Coordinates (Angstroms)			Coordinates (Angstroms)			Coordinates (Angstroms)			Coordinates (Angstroms)			Coordinates (Angstroms)		
	X	Y	Z	X	Y	Z	X	Y	Z	X	Y	Z	X	Y	Z
C	1.80895	-2.10763	-0.42086	-1.38373	-0.12853	-2.23893	1.76713	-1.686	-1.21328	1.80664	-2.10361	-0.43884	1.36099	-1.25676	-0.94271
C	1.10111	-2.00066	-1.55589	-1.00929	-1.3985	-2.46853	1.11276	-1.03723	-2.19007	1.10383	-1.99289	-1.57668	0.71189	-1.06106	-2.1007
C	0.30759	0.91207	-2.48347	0.03631	-3.29989	-0.04694	0.18576	1.98826	-1.99644	0.31757	0.92581	-2.48973	0.26685	1.97878	-1.07615
Cl	-1.38164	1.00476	1.54961	2.82002	-3.31101	1.73001	-1.33563	0.33388	1.80654	-1.39815	0.99565	1.54212	-2.48775	3.65143	-0.13893
O	-4.1526	2.55287	-0.72196	3.95104	-0.56106	2.284	-4.19665	2.63066	0.48324	-4.27253	2.47098	-0.7842	-4.593	2.04431	1.33288
C	-1.37461	-2.04288	-0.73166	1.54077	-1.1413	-2.8969	-1.35829	-1.4806	-1.48896	-1.37539	-2.03839	-0.76451	-1.76002	-0.87937	-2.723
C	-0.30064	-1.47533	-1.67725	0.36947	-1.95883	-2.27449	-0.30893	-0.56271	-2.13623	-0.29692	-1.46556	-1.70169	-0.43984	-0.13672	-2.33773
C	-0.37684	0.06146	-1.39059	0.72018	-2.10143	-0.74706	-0.42303	0.75153	-1.28894	-0.37273	0.06925	-1.40511	-0.80135	0.85051	-1.19907
C	-2.55308	-1.07685	-0.938	2.62905	-1.01085	-1.80538	-2.55989	-0.54414	-1.28492	-2.55241	-1.06896	-0.96474	-2.89921	-0.10907	-2.00913
C	-1.8869	0.33011	-1.15182	2.27198	-2.13965	-0.78948	-1.92812	0.84398	-0.91232	-1.88356	0.33764	-1.17191	-2.24698	1.25156	-1.60537
C	-2.22345	1.35937	-0.06855	3.12357	-1.97264	0.47333	-2.2384	1.31424	0.51245	-2.22624	1.36159	-0.08657	-3.18335	2.00567	-0.65714
C	-3.74208	1.51266	0.18828	3.06459	-0.60051	1.16439	-3.75004	1.30932	0.84762	-3.75292	1.51568	0.15785	-3.66975	1.24755	0.58893
C	-6.02853	-1.59371	0.60178	3.54183	2.64217	-0.91675	-6.00076	-1.71647	-0.05746	-6.00957	-1.60122	0.62603	-5.09505	-2.34649	0.25662
C	-5.70859	-0.0895	0.90017	3.42271	1.9417	0.48872	-5.68665	-0.47638	0.84595	-5.69656	-0.09007	0.9048	-4.84773	-1.06472	1.13721
C	-4.51985	0.20451	-0.03429	3.50546	0.43971	0.13272	-4.52223	0.20698	0.10391	-4.52079	0.20437	-0.04541	-4.37068	-0.02118	0.10122
C	-3.64221	-1.04933	0.13096	2.61413	0.36021	-1.11834	-3.6298	-0.98402	-0.28854	-3.638	-1.04599	0.10825	-3.41464	-0.85228	-0.77052
C	-4.66494	-2.20348	0.13299	3.08039	1.56533	-1.949	-4.64065	-2.05084	-0.75662	-4.65922	-2.19967	0.10749	-4.20579	-2.14712	-1.01117
C	-7.11995	-1.82169	-0.45426	4.94124	3.164	-1.27206	-7.10826	-1.48926	-1.09687	-7.13944	-1.8521	-0.38296	-6.55635	-2.59359	-0.14387
C	-6.87862	0.89984	0.83029	4.36834	2.45728	1.58199	-6.86663	0.42687	1.22527	-6.8722	0.89179	0.83857	-5.99571	-0.65382	2.06924
C	-7.93648	0.68842	1.92084	4.04059	3.88294	2.04568	-7.89626	-0.24908	2.13962	-7.89492	0.70783	1.96678	-6.22492	-1.64	3.22233
C	3.26663	-2.28191	-0.47848	-2.80591	0.18652	-2.07586	3.23228	-1.78897	-1.25024	3.26442	-2.27854	-0.48965	2.60701	-2.03221	-0.87851
C	4.0788	-1.69868	0.57911	-3.17273	1.3066	-1.22083	3.95599	-1.84624	0.01243	4.07109	-1.70238	0.57605	3.45795	-1.86099	0.28954
C	0.46744	2.37654	-2.15449	-1.45387	-3.40597	-0.25924	0.48904	3.11462	-1.04443	0.47715	2.38831	-2.15205	0.66163	2.26367	0.34456
C	1.42854	2.96338	-1.42159	-2.45805	-2.78401	0.37768	1.65892	3.38733	-0.44681	1.43521	2.97049	-1.41144	1.88685	2.36398	0.87924
C	2.51484	2.24191	-0.70686	-2.33498	-1.7742	1.46169	2.93505	2.63378	-0.6318	2.51764	2.24443	-0.69539	3.19279	2.27692	0.16509
N	3.48014	3.02096	-0.14179	-3.5226	-1.22151	1.84425	3.78601	2.71093	0.42981	3.47888	3.02013	-0.1189	4.23741	1.93772	0.97028
O	2.54725	1.01338	-0.59814	-1.26335	-1.44893	1.98381	3.21156	1.98976	-1.64846	2.55013	1.01523	-0.59511	3.33927	2.51833	-1.0392
O	3.85431	-2.77357	-1.47015	-3.72141	-0.56152	-2.48959	3.89623	-1.68394	-2.30547	3.85737	-2.76436	-1.4811	3.04256	-2.71062	-1.83554
C	3.70563	-1.11412	1.88864	-2.38918	2.4556	-0.70284	3.49717	-2.10292	1.40066	3.69105	-1.12654	1.88741	3.19551	-1.24837	1.6136
N	4.80801	-0.42548	2.32531	-3.20615	3.09262	0.19482	4.58163	-1.86472	2.20315	4.7913	-0.44129	2.33473	4.42591	-1.02359	2.18164
C	5.96197	-0.51672	1.43534	-4.47542	2.41554	0.43024	5.7523	-1.36067	1.49524	5.95018	-0.52738	1.45065	5.54018	-1.53859	1.3902
C	5.39246	-1.39221	0.35104	-4.38831	1.32177	-0.59439	5.27097	-1.47213	0.07516	5.38615	-1.39515	0.35732	4.80173	-2.10468	0.20885
O	6.10131	-1.71985	-0.70718	-5.38249	0.4826	-0.78313	6.05495	-1.18245	-0.93938	6.10068	-1.71627	-0.69908	5.43852	-2.66435	-0.79456
O	2.64783	-1.21928	2.50573	-1.25399	2.81183	-1.01055	2.39347	-2.47884	1.78843	2.6299	-1.23538	2.4981	2.11602	-1.00221	2.14353
C	6.51485	0.83673	0.92028	-4.67358	1.90506	1.88329	6.16876	0.07081	1.92921	6.50697	0.82921	0.94817	6.60316	-0.49101	0.98242
C	4.48491	2.44224	0.7516	-3.63352	-0.2636	2.94047	5.0079	1.91902	0.53767	4.47825	2.43597	0.77702	5.63042	1.93369	0.53772
C	5.59569	1.67548	0.0114	-3.45516	1.21309	2.54316	5.05661	1.13523	1.85898	5.59409	1.67449	0.03901	6.19605	0.61355	-0.01899
H	-3.17089	-1.00183	1.11832	1.58819	0.56413	-0.78398	-3.13883	-1.34767	0.62049	-3.16256	-1.00566	1.09391	-2.55024	-1.10264	-0.14044
H	-4.87906	0.2227	-1.0739	4.54124	0.19807	-0.14879	-4.90372	0.65704	-0.8244	-4.89632	0.22504	-1.07843	-5.23065	0.29434	-0.50804
H	-3.03523	-1.34484	-1.8865	3.6274	-1.1809	-2.22212	-3.04976	-0.41591	-2.25818	-3.03752	-1.33099	-1.9134	-3.74073	0.0651	-2.68778
H	-2.31634	0.77739	-2.05676	2.55875	-3.09917	-1.23584	-2.40124	1.61408	-1.53338	-2.30875	0.78852	-2.07712	-2.16174	1.86588	-2.50956
H	-6.35465	-2.07789	1.52659	2.85445	3.49244	-0.94107	-6.30787	-2.55139	0.57857	-6.29193	-2.08334	1.56635	-4.75072	-3.22281	0.81312
H	-5.31168	-0.04592	1.92463	2.39703	2.11549	0.84372	-5.26551	-0.86533	1.78421	-5.28753	-0.03449	1.92417	-3.97873	-1.28094	1.77503
H	0.16914	0.22778	-0.4622	0.38634	-1.19504	-0.23771	0.15215	0.57135	-0.37807	0.16899	0.22908	-0.473	-0.84315	0.30035	-0.25433
H	-0.63692	-1.64505	-2.70727	0.38728	-2.96704	-2.69931	-0.63383	-0.33526	-3.1586	-0.62853	-1.62797	-2.73438	-0.15584	0.47545	-3.20478
H	6.78316	-1.04589	1.93442	-5.31801	3.07047	0.18093	6.61124	-2.02536	1.64031	6.76816	-1.06055	1.95074	6.0479	-2.34471	1.93263
H	1.41003	4.04465	-1.31688	-3.47138	-3.01605	0.06194	1.69096	4.20021	0.27369	1.4172	4.05123	-1.3012	1.9551	2.51024	1.95366
H	-0.27645	3.04299	-2.58569	-1.75919	-4.09803	-1.04122	-0.34961	3.75241	-0.77225	-0.26381	3.0578	-2.5837	-0.16751	2.3664	1.04012
H	1.62814	-2.19851	-2.48573	-1.79829	-2.10513	-2.70466	1.69774	-0.72134	-3.04967	1.63445	-2.18886	-2.50487	1.07415	-1.59372	-2.97783
H	1.39217	-1.85964	0.54784	-0.68197	0.64687	-1.95424	1.28689	-1.98746	-0.28981	1.38546	-1.85802	0.52856	1.07518	-0.75192	-0.02906
H	6.82567	1.41791	1.79498	-4.95937	2.77525	2.47844	6.52627	-0.01659	2.95826	6.81245	1.40433	1.82877	6.99874	-0.04548	1.90071
H	7.43152	0.60286	0.3714	-5.54887	1.2482	1.87717	7.03606	0.36619	1.33044	7.42701	0.59859	0.40358	7.42736	-1.06116	0.54435
H	5.13472	1.03656	-0.74429	-3.20996	1.75618	3.4601	5.19973	1.84336	2.68066	5.13843	1.04096	-0.72441	5.51459	0.224	-0.78039
H	6.21795	2.39736	-0.52692	-2.56883	1.29445	1.91006	4.08346	0.66651	2.03066	6.22008	2.40015	-0.48981	7.10755	0.89339	-0.55698
H	3.97247	1.77605	1.44569	-2.86322	-0.52108	3.66921	5.88535	2.57057	0.45877	3.96169	1.76513	1.46356	6.23774	2.26599	1.38432
H	4.91571	3.2605	1.33225	-4.6047	-0.41618	3.41749	5.02094	1.25588	-0.32569	4.90496	3.25066	1.36569	5.71565	2.6908	-0.24364
H	-1.82561	2.33681	-0.33183	4.16385	-2.15943	0.20479	-1.86383	2.32195	0.67622	-1.81037	2.33564	-0.3379	-4.06703	2.30706	-1.22064
H	-3.88174	1.86723	1.21445	2.03989	-0.38991	1.4967	-3.8603	1.1852	1.92963	-3.89994	1.89499	1.17349	-2.81713	0.97984	1.22645
H	1.29607	0.48263	-2.65671	0.49423	-4.22491	-0.41289	1.10725	1.68839	-2.49122	1.30654	0.49666	-2.66085	1.1556	1.71612	-1.6485
H	-0.2652	0.82458	-3.41321	0.23924	-3.22654	1.01853	-0.51311	2.33927	-2.76392	-0.2511	0.84406	-3.4225	-0.12587	2.89713	-1.52593
H	-4.77076	-2.60635	-0.88031	3.92807	1.26779	-2.57733	-4.76183	-1.9954	-1.84397	-4.79154	-2.57329	-0.91419	-4.84322	-2.02482	-1.89464
H	-4.3451	-3.03165	0.76995	2.30411	1.94201	-2.61868	-4.30001	-3.06359	-0.52837	-4.32458	-3.04603	0.7123	-3.55946	-3.00683	-1.2015
H	-1.63022	-3.07945	-0.965	1.92386	-1.66553	-3.77467	-1.59492	-2.34532	-2.11376	-1.63197	-3.07261	-1.00702	-1.89196	-0.88517	-3.80631
H	-1.02615	-2.0079	0.30573	1.20891	-0.15965	-3.23813	-1.00147	-1.85251	-0.52336	-1.02988	-2.01273	0.27417	-1.73636	-1.92127	-2.39773
H	-7.34838	0.8597	-0.1586	5.40608	2.4073	1.23464	-7.35958	0.80373	0.32244	-7.37016	0.81557	-0.13399	-6.91993	-0.53086	1.49419
H	-6.48501	1.91851	0.94062	4.30483	1.78391	2.43846	-6.47979	1.308	1.75321	-6.47531	1.9114	0.89024	-5.76674	0.32892	2.48492
H	-6.87129	-1.33486	-1.4028	5.68976	2.36598	-1.23453	-6.88002	-0.64143	-1.75086	-6.93665	-1.36721	-1.34339	-6.97722	-1.73605	-0.67865
H	-7.2242	-2.89229	-0.65546	4.9391	3.56085	-2.29218	-7.20761	-2.3739	-1.73327	-7.23574	-2.9255	-0.57382	-6.6143	-3.45641	-0.81487
H	-8.09649	-1.45177	-0.13243	5.27179	3.96643	-0.60865	-8.08198	-1.30559	-0.63618	-8.10743	-1.49433	-0.02366	-7.19817	-2.80231	0.71502
H	-7.49055	0.77642	2.91656	3.02549	3.93453	2.45322	-7.42739	-0.58739	3.06901	-7.42134	0.83457	2.94579	-5.32666	-1.72711	3.84263
H	-8.40033	-0.29907	1.85297	4.10393	4.6102	1.2316	-8.35357	-1.12075	1.6643	-8.35144	-0.28564	1.94886	-6.47696	-2.64328	2.86804
H	-8.73257	1.43434	1.84543	4.72902	4.20716	2.83168	-8.69994	0.44336	2.40497	-8.70119	1.44313	1.89073	-7.04119	-1.30392	3.86849
H	-5.11503	2.60447	-0.71351	3.7063	-1.27995	2.88093	-5.15875	2.65524	0.53652	-3.97641	3.35211	-0.5262	-4.15368	2.87422	1.55933
H	3.41584	4.02421	-0.22087	-4.36662	-1.53546	1.39017	3.49679	3.24867	1.23441	3.4147	4.02384	-0.19186	4.03784	1.69129	1.92738
H	4.87478	-0.08263	3.27198	-2.90138	3.90084	0.71594	4.532	-1.94296	3.20773	4.85327	-0.10567	3.28432	4.5117	-0.85596	3.17376
H	5.47293	-2.24802	-1.27894	-5.06609	-0.1281	-1.50798	5.48693	-1.3229	-1.74983	5.47522	-2.24054	-1.27764	4.72775	-2.89321	-1.45726

Table S7. The Cartesian coordinates of the lowest-energy conformers of (5*S*,6*R*,7*R*,8*R*,9*S*,10*R*,11*R*,13*R*,14*R*,16*S*,23*R*)-**4C.**

4C	Conf. #1			Conf. #2			Conf. #3			Conf. #4			Conf. #5			Conf. #6			Conf. #7		
	Coordinates (Angstroms)			Coordinates (Angstroms)			Coordinates (Angstroms)			Coordinates (Angstroms)			Coordinates (Angstroms)			Coordinates (Angstroms)			Coordinates (Angstroms)		
	X	Y	Z	X	Y	Z	X	Y	Z	X	Y	Z	X	Y	Z	X	Y	Z	X	Y	Z
C	-1.9484	-1.4601	-1.2693	-2.1291	-2.0531	0.0374	-2.0278	-1.4892	-1.2663	-1.9250	-1.6839	-0.8253	-2.0857	-1.9484	-0.3622	-2.0497	-1.7516	0.0643	-2.1307	-2.0524	0.0232
C	-0.6394	-1.3901	-0.9801	-0.9407	-1.6939	0.5478	-0.7282	-1.4361	-0.9380	-0.6428	-1.6344	-0.4326	-0.8779	-1.7246	0.1803	-0.9020	-1.5143	0.7187	-0.9416	-1.6946	0.5333
C	-0.1088	1.7788	-0.9320	-0.4083	0.8083	-1.3612	-0.1177	1.7006	-1.0858	-0.0990	1.3810	0.0631	-0.3009	1.2040	-1.1105	-0.3056	1.5633	0.2256	-0.4163	0.8185	-1.3590
Cl	2.7676	3.4998	-0.7820	2.3953	1.2668	2.0957	2.8221	3.4010	-1.2517	2.5090	3.4249	-0.4709	2.4174	0.5386	2.4688	3.0744	0.9775	2.6531	2.4185	1.2690	2.0798
O	5.0566	2.1691	0.6927	4.3889	2.4448	-1.0436	5.0504	2.2081	0.4213	5.1243	2.3532	0.6144	4.4798	2.6521	-0.0715	4.5522	2.6379	-0.5617	4.4870	2.3429	-1.1470
C	1.6226	-1.4448	-2.1607	1.6085	-1.9330	0.3498	1.5678	-1.6507	-2.0274	1.6523	-1.6540	-1.6019	1.6479	-1.9122	-0.1483	1.6315	-1.7718	0.2637	1.6069	-1.9321	0.3305
C	0.3726	-0.6108	-1.7511	0.2891	-1.4079	-0.2436	0.3319	-0.7506	-1.7337	0.3966	-0.8558	-1.1649	0.3273	-1.2278	-0.5451	0.3189	-0.9937	0.0415	0.2868	-1.4043	-0.2587
C	0.9190	0.6127	-0.9367	0.5137	0.1436	-0.3153	0.8995	0.5237	-1.0148	0.9672	0.3381	-0.3382	0.5696	0.2894	-0.2180	0.7028	0.4335	0.5335	0.5120	0.1473	-0.3229
C	2.8640	-0.5934	-1.8006	2.6834	-1.1018	-0.3689	2.8224	-0.7829	-1.7628	2.8453	-0.6601	-1.5145	2.7281	-0.9198	-0.6047	2.6901	-0.8145	-0.3147	2.6821	-1.0988	-0.3856
C	2.2921	0.8472	-1.6258	2.0416	0.3175	-0.5447	2.2681	0.6750	-1.7371	2.1728	0.7277	-1.2370	2.1091	0.4940	-0.3406	2.1460	0.6262	-0.0027	2.0386	0.3199	-0.5616
C	3.3643	1.7533	-1.0132	2.7192	1.4207	0.2724	3.3642	1.6220	-1.2433	3.2344	1.7430	-0.8027	2.7800	1.2663	0.7985	3.0664	1.4896	0.8661	2.7238	1.4230	0.2480
C	4.0237	1.2689	0.2886	4.2475	1.5054	0.0406	4.0074	1.2830	0.1108	4.1495	1.3399	0.3640	4.3134	1.4019	0.6259	4.5296	1.5343	0.3652	4.2557	1.5052	-0.0012
C	5.3480	-2.3674	0.5740	6.4589	-1.6805	0.0194	5.2822	-2.3201	0.7994	5.9149	-2.1173	0.3365	6.5006	-1.6060	-0.4668	6.4109	-1.7353	-0.5532	6.4564	-1.6845	0.0580
C	5.2465	-0.9136	1.1702	6.2932	-0.1401	0.2489	5.2020	-0.8069	1.2306	5.7576	-0.6832	0.9682	6.3503	-0.2271	0.2593	6.4193	-0.2629	-0.0146	6.2922	-0.1370	0.2510
C	4.6302	-0.1061	0.0051	4.8759	0.1403	-0.2860	4.5969	-0.1236	-0.0172	4.8542	0.0471	-0.0521	4.9309	0.2205	-0.1392	4.9812	0.2159	-0.2888	4.8799	0.1370	-0.2989
C	3.5462	-1.0670	-0.5113	4.0648	-1.0392	0.2801	3.5042	-1.1240	-0.4315	3.8141	-1.0345	-0.3856	4.1144	-1.0744	0.0200	4.1352	-0.9972	0.1380	4.0617	-1.0356	0.2678
C	4.2785	-2.4168	-0.5628	5.0012	-2.2481	0.0791	4.2261	-2.4765	-0.3399	4.6748	-2.2894	-0.5962	5.0406	-2.1617	-0.5636	4.9172	-2.1916	-0.4415	4.9953	-2.2456	0.0716
C	6.7302	-2.7558	0.0302	7.1407	-2.0634	-1.3024	6.6619	-2.7947	0.3222	7.2143	-2.3481	-0.4478	7.1504	-1.5304	-1.8564	6.9244	-1.8999	-1.9909	7.1884	-2.1018	-1.2256
C	6.5245	-0.3564	1.8118	7.4068	0.7687	-0.2861	6.4890	-0.2001	1.8060	7.0584	0.0186	1.3813	7.4686	0.7998	0.0422	7.5380	0.6588	-0.5156	7.4095	0.7583	-0.2974
C	6.9144	-1.0738	3.1110	8.7524	0.5869	0.4277	6.8652	-0.7650	3.1822	7.7534	-0.6449	2.5778	8.8209	0.3779	0.6310	8.9355	0.2638	-0.0216	8.7374	0.6208	0.4579
C	-2.9064	-2.0990	-0.3561	-3.3524	-1.9269	0.8384	-3.0377	-1.9992	-0.3290	-3.0012	-2.2164	0.0159	-3.2656	-2.1685	0.4874	-3.3266	-1.9852	0.7446	-3.3523	-1.9328	0.8278
C	-4.3034	-1.6996	-0.4192	-4.6138	-1.6839	0.1500	-4.4103	-1.5361	-0.4528	-4.3539	-1.7458	-0.2424	-4.5720	-1.7790	-0.0208	-4.5459	-1.6797	0.0103	-4.6156	-1.6865	0.1439
C	-0.3038	2.4138	0.4167	-0.5448	2.3033	-1.2380	-0.2054	2.5223	0.1717	-0.9582	1.9000	-1.0550	-0.5709	2.5572	-0.5130	-0.8492	1.5707	-1.1750	-0.5528	2.3127	-1.2266
C	-1.4521	2.7072	1.0459	-1.5557	2.9763	-0.6688	-1.2446	2.6693	1.0105	-2.2598	2.2203	-1.0079	-1.7576	3.1661	-0.3752	-2.1057	1.8236	-1.5689	-1.5616	2.9819	-0.6492
C	-2.8253	2.4551	0.5273	-2.7109	2.3260	0.0177	-2.5630	1.9859	0.9112	-3.1229	2.1969	0.2089	-3.0602	2.6111	-0.8485	-3.2371	2.2316	-0.6853	-2.7135	2.3267	0.0383
N	-3.7782	2.3548	1.4987	-3.8998	2.9871	-0.0766	-3.3191	2.0293	2.0503	-4.4540	2.0558	-0.0372	-4.1354	2.9451	-0.0801	-4.4740	1.9489	-1.1776	-3.9044	2.9849	-0.0520
O	-3.0936	2.3377	-0.6726	-2.6012	1.2673	0.6400	-2.9622	1.3926	-0.0928	-2.6831	2.3301	1.3573	-3.1626	1.9073	-1.8575	-3.0829	2.8123	0.3959	-2.5994	1.2667	0.6576
O	-2.5541	-2.8810	0.5566	-3.3458	-1.8749	2.0889	-2.7436	-2.7222	0.6523	-2.8009	-2.9426	1.0154	-3.1810	-2.5400	1.6801	-3.4193	-2.3008	1.9523	-3.3431	-1.8894	2.0785
C	-5.0409	-0.8584	-1.3941	-4.9995	-1.8131	-1.2766	-5.0859	-0.7315	-1.5013	-4.9169	-0.9472	-1.3592	-5.0177	-1.3755	-1.3772	-4.7720	-1.3501	-1.4184	-5.0042	-1.8059	-1.2829
N	-6.2281	-0.5319	-0.7890	-6.2636	-1.2934	-1.3723	-6.2920	-0.3586	-0.9698	-6.1574	-0.5363	-0.9454	-6.2408	-0.7758	-1.2113	-6.0471	-0.8516	-1.4974	-6.2691	-1.2869	-1.3721
C	-6.4291	-1.1676	0.5115	-6.7754	-0.7257	-0.1309	-6.5193	-0.8233	0.3927	-6.5469	-1.0512	0.3631	-6.7419	-0.8155	0.1607	-6.7733	-0.8804	-0.2319	-6.7787	-0.7286	-0.1255
C	-5.1420	-1.9370	0.6370	-5.6585	-1.0936	0.8072	-5.2697	-1.6326	0.6076	-5.3220	-1.8467	0.7195	-5.6024	-1.5229	0.8436	-5.7219	-1.4584	0.6741	-5.6595	-1.1023	0.8075
O	-4.8735	-2.6556	1.7026	-5.7220	-0.8173	2.0912	-5.0577	-2.2816	1.7322	-5.2216	-2.4752	1.8682	-5.6136	-1.7485	2.1373	-5.9405	-1.6310	1.9574	-5.7207	-0.8352	2.0936
O	-4.7046	-0.5214	-2.5259	-4.3603	-2.3009	-2.2067	-4.6988	-0.4659	-2.6370	-4.4175	-0.6890	-2.4521	-4.4517	-1.5489	-2.4528	-4.0096	-1.4868	-2.3725	-4.3665	-2.2863	-2.2178
C	-6.6643	-0.1938	1.6950	-7.0842	0.7920	-0.2248	-6.7500	0.3191	1.4213	-6.9012	0.0260	1.4110	-7.0515	0.5646	0.7929	-7.3088	0.4863	0.2465	-7.0884	0.7897	-0.2078
C	-5.1307	1.8658	1.2427	-5.1493	2.3978	0.3976	-4.4376	1.1031	2.2492	-5.4731	2.2189	0.9979	-5.4510	2.3311	-0.2449	-5.7041	2.4346	-0.5560	-5.1519	2.3910	0.4217
C	-5.4572	0.6547	2.1348	-5.9211	1.6749	-0.7222	-5.7017	1.4470	1.4450	-5.7912	0.9898	1.8772	-5.8479	1.5062	0.9925	-6.2923	1.5919	0.5969	-5.9268	1.6766	-0.7015
H	2.7819	-1.1327	0.2751	3.9415	-0.8724	1.3556	2.7406	-1.1013	0.3578	3.2224	-1.1884	0.5271	3.9963	-1.2608	1.0932	4.1618	-1.0619	1.2309	3.9349	-0.8685	1.3428
H	5.3924	0.0286	-0.7768	4.8810	0.0466	-1.3819	5.3632	-0.0832	-0.8055	5.4447	0.2724	-0.9527	4.9263	0.4924	-1.2049	4.8484	0.3411	-1.3737	4.8984	0.0379	-1.3935
H	3.6008	-0.6103	-2.6106	2.8092	-1.5188	-1.3759	3.5557	-0.8928	-2.5687	3.4000	-0.6235	-2.4575	2.8378	-1.0189	-1.6917	2.6756	-0.9314	-1.4053	2.8114	-1.5149	-1.3925
H	2.0782	1.2492	-2.6232	2.2082	0.6434	-1.5785	2.0457	0.9702	-2.7693	1.7711	1.0984	-2.1863	2.3110	1.1267	-1.2129	2.1046	1.1877	-0.9440	2.1988	0.6431	-1.5973
H	5.0860	-3.0844	1.3572	7.0555	-2.0992	0.8346	4.9953	-2.9402	1.6535	5.8764	-2.8579	1.1405	7.1124	-2.2693	0.1509	7.0374	-2.3532	0.0961	7.0170	-2.0892	0.9055
H	4.4801	-0.9465	1.9578	6.2462	0.0157	1.3363	4.4365	-0.7411	2.0169	5.1543	-0.8025	1.8795	6.3135	-0.4386	1.3378	6.5191	-0.3242	1.0785	6.2382	0.0425	1.3347
H	1.0853	0.2859	0.0948	0.2531	0.5502	0.6642	1.0688	0.2724	0.0367	1.3577	-0.0607	0.6042	0.2783	0.4449	0.8249	0.7661	0.3731	1.6237	0.2589	0.5483	0.6610
H	-0.0999	-0.2112	-2.6535	0.1731	-1.7917	-1.2640	-0.0994	-0.4144	-2.6821	-0.0702	-0.4325	-2.0602	0.1781	-1.3285	-1.6260	0.1258	-0.9586	-1.0365	0.1693	-1.7828	-1.2809
H	-7.2760	-1.8622	0.4658	-7.6903	-1.2413	0.1833	-7.3891	-1.4898	0.4338	-7.4051	-1.7271	0.2657	-7.6477	-1.4306	0.2171	-7.6204	-1.5741	-0.2958	-7.6926	-1.2469	0.1868
H	-1.3931	3.1245	2.0477	-1.5632	4.0618	-0.7166	-1.1024	3.2971	1.8862	-2.7465	2.5347	-1.9268	-1.8006	4.1183	0.1465	-2.3421	1.7300	-2.6251	-1.5703	4.0676	-0.6911
H	0.6094	2.6448	0.9592	0.2399	2.8989	-1.6999	0.7005	3.0592	0.4395	-0.4746	2.0128	-2.0231	0.2926	3.0850	-0.1124	-0.1431	1.3157	-1.9633	0.2291	2.9115	-1.6892
H	-0.2812	-1.8949	-0.0841	-0.8916	-1.4548	1.6084	-0.4157	-1.8708	0.0103	-0.3509	-2.1128	0.5010	-0.7791	-1.8166	1.2609	-0.8744	-1.6131	1.8027	-0.8908	-1.4612	1.5951
H	-2.3667	-0.9438	-2.1258	-2.2582	-2.2487	-1.0210	-2.4012	-1.0264	-2.1722	-2.2418	-1.1815	-1.7317	-2.2662	-1.8244	-1.4240	-2.1075	-1.6168	-1.0094	-2.2617	-2.2423	-1.0359
H	-7.5169	0.4428	1.4389	-7.9373	0.8863	-0.9013	-7.7260	0.7495	1.1808	-7.7530	0.5950	1.0244	-7.8242	1.0461	0.1855	-8.0098	0.8499	-0.5119	-7.9431	0.8887	-0.8815
H	-6.9840	-0.8076	2.5415	-7.4392	1.1232	0.7562	-6.8561	-0.1406	2.4097	-7.2722	-0.5158	2.2861	-7.5099	0.3614	1.7645	-7.9088	0.2791	1.1377	-7.4412	1.1136	0.7765
H	-5.6481	1.0097	3.1527	-5.2167	1.0713	-1.3007	-5.4174	1.7009	0.4234	-6.1261	1.3884	2.8400	-6.0850	2.1942	1.8101	-6.8268	2.2963	1.2420	-5.2239	1.0776	-1.2866
H	-4.5672	0.0222	2.2020	-6.3123	2.4255	-1.4148	-6.1631	2.3387	1.8798	-4.8644	0.4504	2.0906	-4.9752	0.9360	1.3242	-5.4755	1.1979	1.2079	-6.3201	2.4325	-1.3871
H	-5.1711	1.6079	0.1874	-5.7665	3.1892	0.8308	-4.6653	1.1010	3.3169	-6.3817	2.5738	0.5059	-5.3904	1.7048	-1.1316	-6.4453	2.5543	-1.3498	-5.7684	3.1782	0.8633
H	-5.8605	2.6647	1.4140	-4.8872	1.7055	1.1967	-4.0888	0.1016	1.9933	-5.1245	3.0186	1.6544	-6.2003	3.1071	-0.4330	-5.4820	3.4292	-0.1646	-4.8865	1.6926	1.2143
H	4.1570	1.8864	-1.7501	2.2891	2.3919	0.0378	4.1597	1.6345	-1.9892	3.8751	1.9491	-1.6607	2.3659	2.2684	0.8831	2.6968	2.5117	0.9160	2.2802	2.3920	0.0251
H	3.2734	1.1881	1.0859	4.7085	1.9297	0.9383	3.2494	1.3016	0.9047	3.5539	1.1679	1.2704	4.7657	1.4868	1.6192	5.1736	1.7764	1.2164	4.7226	1.9586	0.8783
H	-1.0715	1.4263	-1.2977	-1.3944	0.3529	-1.2691	-1.1037	1.3025	-1.3106	0.3963	2.2248	0.5503	-1.2496	0.7145	-1.3246	0.1915	2.5247	0.4148	-1.4018	0.3625	-1.2639
H	0.2191	2.5500	-1.6379	-0.0255	0.5760	-2.3622	0.1650	2.3638	-1.9109	-0.7380	0.9403	0.8300	0.2024	1.3311	-2.0784	-1.1322	1.5235	0.9346	-0.0394	0.5923	-2.3636
H	3.6018	-3.2676	-0.4559	4.8826	-2.9920	0.8705	3.5424	-3.3087	-0.1577	4.1289	-3.2137	-0.3934	4.9292	-3.1144	-0.0404	4.8061	-3.0908	0.1693	4.8527	-3.0047	0.8446
H	4.7755	-2.5242	-1.5340	4.7619	-2.7496	-0.8649	4.7341	-2.6822	-1.2893	5.0016	-2.3345	-1.6416	4.7840	-2.3452	-1.6125	4.5323	-2.4357	-1.4379	4.7776	-2.7268	-0.8886
H	1.5829	-1.6658	-3.2290	1.7329	-3.0084	0.2008	1.5319	-2.0055	-3.0592	1.5196	-2.0573	-2.6075	1.7560	-2.8987	-0.6059	1.6284	-2.7473	-0.2280	1.7305	-3.0070	0.1771
H	1.6475	-2.4062	-1.6423	1.6353	-1.7392	1.4279	1.5695	-2.5384	-1.3900	1.8179	-2.5058	-0.9369	1.6892	-2.0420	0.9386	1.7954	-1.9345	1.3351	1.6349	-1.7427	1.4093
H	7.3534	-0.4023	1.0972	7.5325	0.6179	-1.3638	7.3175	-0.3447	1.1042	7.7458	0.0659	0.5297	7.5784	1.0188	-1.0256	7.5275	0.7048	-1.6100	7.5626	0.5565	-1.3630
H	6.3699	0.7027	2.0254	7.0998	1.8145	-0.1568	6.3530	0.8793	1.8924	6.8250	1.0533	1.6378	7.1780	1.7438	0.5209	7.3357	1.6792	-0.1656	7.0792	1.8008	-0.2369
H	6.6802	-3.7470	-0.4312	7.1459	-3.1517	-1.4160	6.5955	-3.8303	-0.0260	7.1951	-3.3400	-0.9102	7.1471	-2.5204	-2.3225	6.8105	-2.9408	-2.3088	7.1886	-3.1925	-1.3155
H	7.4931	-2.7954	0.8109	8.1781	-1.7236	-1.3507	7.4150	-2.7624	1.1128	8.1026	-2.2988	0.1860	8.1883	-1.1913	-1.8137	7.9813	-1.6405	-2.0896	8.2298	-1.7710	-1.2387
H	7.0709	-2.0551	-0.7388	6.6100	-1.6484	-2.1653	7.0267	-2.1921	-0.5158	7.3332	-1.6156	-1.2526	6.6029	-0.8552	-2.5219	6.3585	-1.2808	-2.6945	6.6967	-1.7012	-2.1180
H	6.1188	-0.9836	3.8581	8.6530	0.7838	1.4998	6.0713	-0.5716	3.9113	7.0994	-0.6430	3.4562	8.7375	0.2016	1.7080	8.9757	0.2667	1.0722	8.6112	0.8724	1.5160
H	7.8218	-0.6397	3.5410	9.5034	1.2755	0.0309	7.7806	-0.2997	3.5596	8.6682	-0.1083	2.8461	9.5748	1.1551	0.4781	9.6928	0.9642	-0.3844	9.4959	1.2925	0.0456
H	7.1003	-2.1404	2.9576	9.1406	-0.4285	0.3132	7.0311	-1.8456	3.1575	8.0280	-1.6839	2.3761	9.1956	-0.5416	0.1739	9.2201	-0.7361	-0.3595	9.1349	-0.3966	0.4073
H	4.6661	3.0480	0.7826	5.3047	2.4313	-1.3437	4.6735	3.0972	0.4045	4.6558	3.1798	0.7882	5.3983	2.7270	-0.3540	5.4013	2.6381	-1.0178	4.3177	3.2598	-0.8998
H	-3.4809	2.4345	2.4607	-3.9490	3.8051	-0.6670	-2.9100	2.4453	2.8734	-4.7538	1.9192	-0.9902	-3.9683	3.4895	0.7545	-4.5408	1.4459	-2.0491	-3.9567	3.8042	-0.6402
H	-6.9901	-0.1311	-1.3161	-6.7619	-1.2531	-2.2485	-6.9849	0.1372	-1.5097	-6.8264	-0.1533	-1.5972	-6.8444	-0.5970	-2.0005	-6.5161	-0.7561	-2.3863	-6.7692	-1.2408	-2.2469
H	-3.9404	-2.9872	1.5591	-4.8622	-1.1627	2.4673	-4.1436	-2.6761	1.6276	-4.3026	-2.8677	1.8684	-4.7217	-2.1565	2.3338	-5.0747	-1.9706	2.3234	-4.8596	-1.1823	2.4655

Table S8. Conformers and Boltzmann distributions of the optimized **5**.

Comp. **5**	Conf. No.	BOLTZMANN POPULATION,%	TOTAL GIBBS FREE ENERGY, KCAL/MOL
7S,8S,23S-**5A**	#1	14.9357	447.1714
	#2	10.6068	447.3030
	#3	8.8506	447.1550
	#4	6.5341	447.2297
	#5	6.4614	448.1514
	#6	5.3631	448.1601
7R,8R,23R-**5B**	#1	14.9342	447.1714
	#2	10.6058	447.3030
	#3	8.8497	447.1550
	#4	6.5335	447.2297
	#5	6.4608	448.1514
	#6	5.3626	448.1601
7S,8S,23R-**5C**	#1	14.6727	446.5555
	#2	9.1586	446.7934
	#3	5.5068	447.4018
	#4	5.4470	447.0105

Table S9. The Cartesian coordinates of the lowest-energy conformers of (5*S*,6*R*,7*S*,8*S*,9*R*,10*R*,11*R*,13*R*,14*R*,16*S*,23*S*,29*S*)-**5A.**

5A	Conf. #1			Conf. #2			Conf. #3			Conf. #4			Conf. #5			Conf. #6		
	Coordinates (Angstroms)			Coordinates (Angstroms)			Coordinates (Angstroms)			Coordinates (Angstroms)			Coordinates (Angstroms)			Coordinates (Angstroms)		
	X	Y	Z	X	Y	Z	X	Y	Z	X	Y	Z	X	Y	Z	X	Y	Z
C	-2.1564	2.2029	0.2801	-2.1574	2.1958	0.3363	-2.0803	-0.3305	-2.0819	-2.0902	-0.3875	-2.0781	-2.0237	1.4834	1.6765	-2.0325	1.5194	1.6366
C	-1.6832	2.3632	1.5253	-1.6848	2.3234	1.5855	-1.6529	0.8643	-2.5205	-1.6588	0.7971	-2.5398	-1.5266	0.7497	2.6837	-1.5261	0.8129	2.6587
C	-0.7763	-0.2553	2.9340	-0.7810	-0.3297	2.9296	-0.5667	3.0619	-0.5587	-0.5685	3.0489	-0.6447	-0.7727	-2.1947	2.0180	-0.7728	-2.1421	2.0524
O	3.5730	-0.6643	2.9214	3.4831	-0.6930	2.9756	3.8443	2.9371	-0.4832	3.7590	2.9739	-0.6411	3.4416	-2.7735	1.4642	3.4433	-2.6305	1.5803
Cl	1.7670	-1.6863	-0.4587	1.7652	-1.6691	-0.5298	1.8628	0.3730	1.8138	1.8635	0.4003	1.8104	1.5627	-1.2393	-1.7186	1.5822	-1.2606	-1.7152
C	0.8620	2.7478	1.1857	0.8598	2.7110	1.2517	0.8634	0.3843	-2.9484	0.8561	0.2946	-2.9481	1.0334	1.1601	2.5228	1.0323	1.2244	2.4721
C	-0.2796	2.0709	1.9776	-0.2814	2.0178	2.0293	-0.2392	1.3688	-2.4897	-0.2433	1.2966	-2.5225	-0.1378	0.1781	2.7548	-0.1357	0.2462	2.7341
C	0.0815	0.5513	1.9277	0.0759	0.4992	1.9405	0.2107	1.8110	-1.0583	0.2058	1.7803	-1.1045	0.1095	-0.9592	1.7116	0.1094	-0.9141	1.7160
C	2.1079	1.8262	1.3367	2.1044	1.7848	1.3801	2.1430	0.7362	-2.1350	2.1371	0.6710	-2.1481	2.1992	0.3251	1.9178	2.2004	0.3737	1.8945
C	1.6061	0.6069	2.1812	1.6005	0.5433	2.1911	1.7358	1.9712	-1.2732	1.7305	1.9310	-1.3220	1.6410	-1.1383	1.8308	1.6405	-1.0888	1.8351
C	2.4251	-0.6975	2.0429	2.4067	-0.7593	2.0139	2.6422	2.2589	-0.0560	2.6261	2.2525	-0.1110	2.3459	-2.0835	0.8272	2.3370	-2.0552	0.8526
C	3.0554	-0.9794	0.6657	3.0493	-0.9972	0.6298	3.1858	1.0316	0.6996	3.1816	1.0435	0.6726	2.9125	-1.4368	-0.4601	2.9212	-1.4364	-0.4404
C	4.5489	1.7808	-1.6239	4.5536	1.8259	-1.5757	4.3240	-2.2394	-1.0646	4.3259	-2.2725	-1.0018	3.8926	2.1938	-0.9834	3.9313	2.1720	-1.0277
C	4.3573	0.2522	-1.3097	4.3695	0.2886	-1.2974	4.2438	-1.3750	0.2474	4.2571	-1.3646	0.2821	4.2830	0.6978	-1.2926	4.3180	0.6677	-1.2982
C	3.7006	0.2692	0.0854	3.7020	0.2665	0.0926	3.7063	-0.0235	-0.2645	3.7080	-0.0318	-0.2653	3.5969	-0.1153	-0.1564	3.6134	-0.1166	-0.1536
C	2.6812	1.4110	-0.0216	2.6838	1.4113	0.0128	2.6233	-0.4355	-1.2719	2.6241	-0.4763	-1.2570	2.6315	0.8496	0.5476	2.6462	0.8686	0.5184
C	3.4276	2.5032	-0.8094	3.4367	2.5254	-0.7359	3.2524	-1.6235	-2.0211	3.2585	-1.6788	-1.9766	3.3781	2.1834	0.4707	3.4010	2.1965	0.4205
C	5.9242	2.3632	-1.2571	5.9293	2.4065	-1.2079	5.6955	-2.2643	-1.7598	5.6946	-2.3371	-1.6996	4.9373	3.2717	-1.2749	4.9833	3.2389	-1.3336
C	5.6203	-0.6012	-1.4856	5.6400	-0.5515	-1.4836	5.5337	-1.3535	1.0785	5.5566	-1.3109	1.0971	5.7909	0.4201	-1.4220	5.8259	0.3803	-1.4030
C	6.0731	-0.6597	-2.9482	6.0914	-0.5879	-2.9474	5.8552	-2.7302	1.6692	5.8785	-2.6614	1.7452	6.1038	-0.9873	-1.9407	6.1375	-1.0414	-1.8823
C	-3.6046	2.1912	0.0349	-3.6051	2.1908	0.0890	-3.5178	-0.5864	-1.9217	-3.5281	-0.6326	-1.9073	-3.4731	1.6890	1.5560	-3.4830	1.7192	1.5218
C	-4.1181	1.3751	-1.0548	-4.1171	1.4016	-1.0210	-3.9535	-1.5327	-0.9048	-3.9644	-1.5510	-0.8649	-4.0487	1.8131	0.2252	-4.0683	1.8147	0.1927
C	-0.7267	-1.7624	2.8594	-0.7359	-1.8343	2.8161	-0.6570	3.1859	0.9390	-0.6656	3.2179	0.8482	-0.8434	-3.2884	0.9795	-0.8477	-3.2586	1.0389
C	-1.4361	-2.5806	2.0649	-1.4497	-2.6305	2.0031	-1.6875	2.8657	1.7360	-1.6947	2.9120	1.6528	-1.6231	-3.3519	-0.1122	-1.6328	-3.3476	-0.0474
C	-2.3661	-2.1329	0.9952	-2.3839	-2.1545	0.9495	-3.0022	2.3066	1.3072	-3.0026	2.3262	1.2403	-2.5299	-2.2691	-0.5756	-2.5435	-2.2770	-0.5306
N	-3.1688	-3.0957	0.4603	-3.1830	-3.1043	0.3864	-3.6443	1.5985	2.2794	-3.6550	1.6708	2.2429	-3.3855	-2.5981	-1.5842	-3.4024	-2.6292	-1.5286
O	-2.4112	-0.9694	0.5876	-2.4358	-0.9792	0.5778	-3.4822	2.4553	0.1794	-3.4699	2.4114	0.1008	-2.5128	-1.1298	-0.1027	-2.5268	-1.1277	-0.0827
O	-4.4303	2.7279	0.8103	-4.4318	2.7097	0.8754	-4.3947	0.0648	-2.5331	-4.4044	0.0056	-2.5330	-4.2548	1.6119	2.5326	-4.2580	1.6605	2.5051
C	-3.4265	0.6815	-2.1673	-3.4234	0.7311	-2.1464	-3.2066	-2.5327	-0.1013	-3.2190	-2.5346	-0.0401	-3.4140	1.9928	-1.1023	-3.4442	1.9709	-1.1426
N	-4.3361	-0.2037	-2.6863	-4.3349	-0.1367	-2.6907	-4.1053	-3.0313	0.8054	-4.1166	-3.0070	0.8817	-4.3830	1.7038	-2.0286	-4.4187	1.6596	-2.0560
C	-5.6513	-0.1496	-2.0543	-5.6519	-0.0939	-2.0615	-5.4081	-2.3769	0.7786	-5.4166	-2.3476	0.8435	-5.6859	1.3854	-1.4514	-5.7162	1.3480	-1.4630
C	-5.4121	0.9320	-1.0346	-5.4125	0.9621	-1.0154	-5.2344	-1.4915	-0.4237	-5.2431	-1.4920	-0.3802	-5.3678	1.5187	0.0140	-5.3879	1.5116	-0.0030
O	-6.3535	1.2999	-0.1929	-6.3554	1.3129	-0.1682	-6.2210	-0.7530	-0.8797	-6.2279	-0.7600	-0.8508	-6.2749	1.2797	0.9359	-6.2874	1.2881	0.9302
O	-2.2867	0.8549	-2.5923	-2.2803	0.9078	-2.5615	-2.0357	-2.8886	-0.2113	-2.0502	-2.8989	-0.1457	-2.2693	2.3538	-1.3660	-2.3030	2.3313	-1.4220
C	-6.1476	-1.4811	-1.4364	-6.1544	-1.4385	-1.4777	-5.7522	-1.6233	2.0923	-5.7528	-1.5626	2.1407	-6.2627	-0.0009	-1.8348	-6.2904	-0.0477	-1.8148
C	-4.0485	-2.7997	-0.6696	-4.0602	-2.7795	-0.7377	-4.8431	0.8029	2.0327	-4.8518	0.8641	2.0265	-4.2429	-1.5887	-2.2042	-4.2669	-1.6358	-2.1644
C	-5.3313	-2.0524	-0.2610	-5.3436	-2.0414	-0.3144	-4.6881	-0.6243	2.5824	-4.6851	-0.5527	2.6002	-5.4812	-1.2403	-1.3577	-5.5015	-1.2746	-1.3179
H	1.8560	1.0427	-0.6431	1.8624	1.0626	-0.6246	1.7707	-0.8084	-0.6910	1.7751	-0.8383	-0.6639	1.7345	0.9441	-0.0776	1.7551	0.9558	-0.1166
H	4.4647	0.5460	0.8265	4.4600	0.5210	0.8480	4.5140	0.4873	-0.8087	4.5097	0.4703	-0.8266	4.3856	-0.3673	0.5657	4.3805	-0.3729	0.5871
H	2.8996	2.3414	1.8904	2.8939	2.2842	1.9514	2.9611	1.0162	-2.8062	2.9521	0.9343	-2.8299	3.0699	0.3488	2.5828	3.0659	0.4019	2.5646
H	1.7093	0.8889	3.2381	1.7195	0.7834	3.2539	1.8367	2.8594	-1.9118	1.8466	2.8030	-1.9761	1.8050	-1.6124	2.8062	1.8140	-1.5444	2.8167
H	4.3940	1.9409	-2.6941	4.3888	2.0099	-2.6405	4.0558	-3.2706	-0.8204	4.0470	-3.2918	-0.7222	3.0229	2.4111	-1.6153	3.0692	2.3783	-1.6736
H	3.6095	-0.1325	-2.0134	3.6294	-0.0844	-2.0153	3.4622	-1.8144	0.8786	3.4845	-1.7859	0.9363	3.8471	0.4256	-2.2581	3.8941	0.3778	-2.2642
H	-0.1321	0.1817	0.9230	-0.1433	0.1557	0.9277	0.0124	0.9786	-0.3774	0.0030	0.9694	-0.3993	-0.1617	-0.5825	0.7236	-0.1655	-0.5587	0.7208
H	-0.1982	2.3772	3.0270	-0.1975	2.2978	3.0857	-0.1927	2.2642	-3.1199	-0.1920	2.1724	-3.1794	-0.0154	-0.2755	3.7450	-0.0059	-0.1844	3.7337
H	-6.4046	0.1892	-2.7759	-6.4020	0.2648	-2.7770	-6.2051	-3.1027	0.5827	-6.2173	-3.0745	0.6672	-6.4220	2.1474	-1.7356	-6.4571	2.1015	-1.7569
H	-1.3203	-3.6533	2.1931	-1.3347	-3.7063	2.1028	-1.5519	2.9724	2.8090	-1.5634	3.0572	2.7218	-1.5870	-4.2514	-0.7204	-1.5990	-4.2609	-0.6348
H	-0.0749	-2.2511	3.5800	-0.0823	-2.3429	3.5212	0.2321	3.5521	1.4460	0.2169	3.6119	1.3460	-0.2223	-4.1611	1.1682	-0.2256	-4.1268	1.2445
H	-2.4029	2.6238	2.2971	-2.4043	2.5642	2.3638	-2.4147	1.5691	-2.8414	-2.4187	1.4995	-2.8703	-2.2198	0.4484	3.4648	-2.2129	0.5313	3.4527
H	-1.5279	1.8770	-0.5400	-1.5285	1.8908	-0.4915	-1.4091	-1.0815	-1.6814	-1.4211	-1.1334	-1.6648	-1.4258	1.7694	0.8195	-1.4415	1.7839	0.7681
H	-7.1719	-1.2986	-1.0994	-7.1791	-1.2608	-1.1393	-6.7216	-1.1334	1.9571	-6.7225	-1.0751	1.9993	-7.2723	-0.0384	-1.4160	-7.2971	-0.0803	-1.3887
H	-6.2208	-2.2139	-2.2470	-6.2278	-2.1509	-2.3063	-5.9038	-2.3929	2.8534	-5.9010	-2.3139	2.9204	-6.3851	-0.0173	-2.9230	-6.4198	-0.0865	-2.9016
H	-5.0611	-1.2512	0.4303	-5.0749	-1.2588	0.3985	-4.7278	-0.5849	3.6752	-4.7111	-0.4936	3.6925	-5.1666	-1.1067	-0.3204	-5.1814	-1.1192	-0.2853
H	-5.9725	-2.7418	0.2970	-5.9880	-2.7442	0.2229	-3.6912	-0.9964	2.3295	-3.6901	-0.9260	2.3416	-6.1617	-2.0974	-1.3686	-6.1790	-2.1341	-1.3076
H	-4.3007	-3.7477	-1.1494	-4.3119	-3.7153	-1.2412	-4.9972	0.8086	0.9551	-5.0185	0.8489	0.9510	-4.5489	-1.9707	-3.1804	-4.5770	-2.0388	-3.1308
H	-3.4821	-2.2032	-1.3842	-3.4920	-2.1660	-1.4364	-5.7174	1.2840	2.4854	-5.7223	1.3501	2.4812	-3.6407	-0.6956	-2.3683	-3.6689	-0.7441	-2.3505
H	1.8041	-1.5546	2.3145	1.7762	-1.6194	2.2505	2.1200	2.8995	0.6583	2.0937	2.9028	0.5864	1.6679	-2.8832	0.5331	1.6478	-2.8538	0.5698
H	3.7679	-1.7904	0.7633	3.7595	-1.8159	0.6918	3.9534	1.3529	1.3941	3.9482	1.3749	1.3658	3.5736	-2.1600	-0.9296	3.5835	-2.1659	-0.9023
H	-1.8118	0.0539	2.7800	-1.8163	-0.0139	2.7873	-1.5771	3.0275	-0.9602	-1.5772	3.0058	-1.0496	-1.7860	-1.8222	2.1807	-1.7854	-1.7657	2.2103
H	-0.4889	0.0455	3.9482	-0.4880	-0.0558	3.9497	-0.0803	3.9590	-0.9609	-0.0758	3.9312	-1.0706	-0.4283	-2.6385	2.9590	-0.4250	-2.5650	3.0018
H	2.7579	3.0749	-1.4562	2.7712	3.1253	-1.3611	3.7396	-1.2674	-2.9354	2.5168	-2.4243	-2.2724	2.7477	3.0495	0.6901	2.7726	3.0706	0.6138
H	3.8818	3.2176	-0.1142	3.8959	3.2103	-0.0147	2.5073	-2.3613	-2.3272	3.7509	-1.3401	-2.8948	4.2191	2.1904	1.1748	4.2340	2.2148	1.1338
H	1.0433	3.7626	1.5465	1.0429	3.7172	1.6348	1.0214	0.4591	-4.0263	1.0134	0.3315	-4.0281	1.3154	1.6585	3.4527	1.3116	1.7536	3.3856
H	0.5867	2.8303	0.1311	0.5831	2.8174	0.1996	0.5645	-0.6464	-2.7453	0.5550	-0.7278	-2.7092	0.7360	1.9435	1.8216	0.7312	1.9838	1.7461
O	5.3333	-1.9257	-1.0016	5.3661	-1.8838	-1.0132	5.3701	-0.3860	2.1311	5.4096	-0.2948	2.1057	6.4034	0.6558	-0.1432	6.4266	0.6452	-0.1246
H	6.4275	-0.1912	-0.8638	6.4441	-0.1415	-0.8579	6.3680	-1.0162	0.4489	6.3853	-1.0065	0.4438	6.2069	1.1449	-2.1347	6.2536	1.0845	-2.1296
H	5.9206	3.4430	-1.4336	5.9184	3.4900	-1.3596	5.6156	-2.8175	-2.7004	5.6049	-2.9212	-2.6204	4.5214	4.2602	-1.0563	4.5694	4.2340	-1.1427
H	6.7396	1.9404	-1.8484	6.7415	2.0019	-1.8158	6.4702	-2.7501	-1.1625	6.4658	-2.8114	-1.0885	5.2383	3.2729	-2.3270	5.2950	3.2141	-2.3823
H	6.1585	2.2077	-0.1991	6.1736	2.2284	-0.1558	6.0409	-1.2549	-2.0051	6.0507	-1.3408	-1.9805	5.8328	3.1420	-0.6633	5.8723	3.1203	-0.7102
H	6.2881	0.3359	-3.3409	6.2855	0.4156	-3.3304	5.9651	-3.4845	0.8878	5.9670	-3.4514	0.9973	5.5858	-1.1670	-2.8864	5.6288	-1.2434	-2.8286
H	6.9789	-1.2593	-3.0614	7.0088	-1.1680	-3.0687	6.7857	-2.7122	2.2407	6.8199	-2.6262	2.2978	7.1733	-1.1124	-2.1237	7.2083	-1.1767	-2.0502
H	5.2850	-1.1058	-3.5617	5.3107	-1.0433	-3.5636	5.0466	-3.0443	2.3355	5.0800	-2.9373	2.4400	5.7948	-1.7516	-1.2237	5.8170	-1.7848	-1.1486
C	6.4805	-2.7010	-0.6857	6.5212	-2.6593	-0.7273	6.5845	0.0620	2.7160	6.6316	0.1603	2.6692	7.8112	0.8390	-0.1815	7.8351	0.8227	-0.1541
H	7.0753	-2.9469	-1.5728	7.0961	-2.8993	-1.6290	7.0974	-0.7312	3.2720	7.1290	-0.6125	3.2663	8.3416	-0.0757	-0.4694	8.3652	-0.1003	-0.4149
H	7.1233	-2.1806	0.0365	7.1795	-2.1419	-0.0169	7.2686	0.4574	1.9537	7.3238	0.5030	1.8889	8.0880	1.6411	-0.8785	8.1216	1.6069	-0.8675
H	6.1239	-3.6297	-0.2380	6.1759	-3.5910	-0.2771	6.3268	0.8627	3.4106	6.3881	1.0002	3.3214	8.1236	1.1210	0.8248	8.1392	1.1279	0.8480
H	3.2596	-0.5164	3.8207	3.9273	-1.5489	2.9993	3.5889	3.7580	-0.9191	4.2717	3.3262	0.0961	3.9890	-2.1363	1.9377	3.8044	-3.3623	1.0660
H	-3.1206	-4.0384	0.8154	-3.1288	-4.0574	0.7112	-3.1939	1.5089	3.1789	-3.2144	1.6303	3.1507	-3.3825	-3.5366	-1.9529	-3.4005	-3.5759	-1.8760
H	-4.1741	-0.6693	-3.5669	-4.1701	-0.5847	-3.5797	-3.8378	-3.7038	1.5083	-3.8495	-3.6631	1.6000	-4.2606	1.9272	-3.0050	-4.3047	1.8654	-3.0373
H	-5.9141	1.9768	0.3981	-5.9152	1.9739	0.4404	-5.8300	-0.2584	-1.6569	-5.8376	-0.2871	-1.6416	-5.7868	1.3805	1.8032	-5.7931	1.4085	1.7917

Table S10. The Cartesian coordinates of the lowest-energy conformers of (5*R*,6*S*,7*R*,8*R*,9*S*,10*S*,11*S*,13*S*,14*S*,16*R*,23*R,*29*R*)-**5B.**

5B	Conf. #1			Conf. #2			Conf. #3			Conf. #4			Conf. #5			Conf. #6		
	Coordinates (Angstroms)			Coordinates (Angstroms)			Coordinates (Angstroms)			Coordinates (Angstroms)			Coordinates (Angstroms)			Coordinates (Angstroms)		
	X	Y	Z	X	Y	Z	X	Y	Z	X	Y	Z	X	Y	Z	X	Y	Z
C	2.15636	2.20288	0.28011	2.28206	1.79993	0.59632	2.08031	-0.33048	-2.08192	2.09016	-0.38746	-2.07812	2.02368	1.48332	1.67656	2.02368	1.48332	1.67656
C	1.68320	2.36322	1.52533	1.35793	2.46665	1.37094	1.65288	0.86425	-2.52049	1.65882	0.79708	-2.53975	1.52661	0.74960	2.68377	1.52661	0.74960	2.68377
C	0.77625	-0.25534	2.93395	0.82585	-0.58909	2.46686	0.56675	3.06192	-0.55871	0.56850	3.04886	-0.64474	0.77272	-2.19483	2.01789	0.77272	-2.19483	2.01789
O	-3.57298	-0.66425	2.92135	-3.38492	-1.26283	2.78903	-3.84428	2.93716	-0.48307	-3.75896	2.97387	-0.64107	-3.44161	-2.77361	1.46410	-3.44161	-2.77361	1.46410
Cl	-1.76695	-1.68628	-0.45865	-1.93364	-1.46108	-0.96088	-1.86275	0.37295	1.81386	-1.86352	0.40030	1.81044	-1.56268	-1.23923	-1.71864	-1.56268	-1.23923	-1.71864
C	-0.86197	2.74783	1.18570	-1.17043	2.58527	1.60958	-0.86343	0.38433	-2.94838	-0.85611	0.29462	-2.94809	-1.03346	1.15997	2.52290	-1.03346	1.15997	2.52290
C	0.27958	2.07093	1.97764	0.14002	1.83515	1.98986	0.23916	1.36882	-2.48972	0.24326	1.29659	-2.52254	0.13781	0.17797	2.75480	0.13781	0.17797	2.75480
C	-0.08148	0.55126	1.92767	-0.15251	0.33682	1.69207	-0.21062	1.81097	-1.05824	-0.20580	1.78030	-1.10450	-0.10950	-0.95931	1.71160	-0.10950	-0.95931	1.71160
C	-2.10793	1.82619	1.33670	-2.31620	1.53892	1.62069	-2.14303	0.73621	-2.13499	-2.13712	0.67095	-2.14814	-2.19920	0.32493	1.91780	-2.19920	0.32493	1.91780
C	-1.60609	0.60692	2.18118	-1.64277	0.21610	2.10486	-1.73581	1.97118	-1.27317	-1.73053	1.93101	-1.32202	-1.64096	-1.13837	1.83069	-1.64096	-1.13837	1.83069
C	-2.42506	-0.69750	2.04290	-2.39691	-1.08154	1.74983	-2.64212	2.25887	-0.05598	-2.62614	2.25251	-0.11097	-2.34592	-2.08359	0.82707	-2.34592	-2.08359	0.82707
C	-3.05543	-0.97940	0.66571	-3.14690	-1.10463	0.40004	-3.18573	1.03157	0.69967	-3.18157	1.04349	0.67255	-2.91250	-1.43682	-0.46013	-2.91250	-1.43682	-0.46013
C	-4.54889	1.78080	-1.62390	-4.96580	1.96998	-1.13300	-4.32405	-2.23935	-1.06465	-4.32594	-2.27254	-1.00180	-3.89246	2.19379	-0.98339	-3.89246	2.19379	-0.98339
C	-4.35730	0.25221	-1.30970	-4.68843	0.41933	-1.15039	-4.24379	-1.37497	0.24734	-4.25706	-1.36463	0.28208	-4.28301	0.69777	-1.29262	-4.28301	0.69777	-1.29262
C	-3.70058	0.26916	0.08541	-3.91427	0.18595	0.16276	-3.70630	-0.02350	-0.26451	-3.70803	-0.03183	-0.26531	-3.59688	-0.11534	-0.15637	-3.59688	-0.11534	-0.15637
C	-2.68115	1.41103	-0.02157	-2.97741	1.39749	0.24595	-2.62333	-0.43548	-1.27194	-2.62406	-0.47631	-1.25702	-2.63144	0.84953	0.54762	-2.63144	0.84953	0.54762
C	-3.42757	2.50324	-0.80938	-3.86035	2.57130	-0.20732	-3.25242	-1.62340	-2.02116	-3.25847	-1.67878	-1.97663	-3.37807	2.18333	0.47069	-3.37807	2.18333	0.47069
C	-5.92423	2.36315	-1.25712	-6.35594	2.39835	-0.63471	-5.69549	-2.26421	-1.75986	-5.69464	-2.33712	-1.69960	-4.93712	3.27180	-1.27490	-4.93712	3.27180	-1.27490
C	-5.62033	-0.60115	-1.48556	-5.93053	-0.44754	-1.39964	-5.53368	-1.35347	1.07846	-5.55659	-1.31091	1.09709	-5.79086	0.42016	-1.42191	-5.79086	0.42016	-1.42191
C	-6.07305	-0.65965	-2.94819	-6.46543	-0.27791	-2.82533	-5.85516	-2.73024	1.66907	-5.87851	-2.66135	1.74523	-6.10391	-0.98717	-1.94065	-6.10391	-0.98717	-1.94065
C	3.60458	2.19122	0.03488	3.42941	2.29762	-0.01946	3.51772	-0.58642	-1.92164	3.52809	-0.63263	-1.90726	3.47307	1.68897	1.55608	3.47307	1.68897	1.55608
C	4.11808	1.37510	-1.05478	4.38970	1.46851	-0.77177	3.95350	-1.53274	-0.90474	3.96439	-1.55104	-0.86489	4.04862	1.81310	0.22529	4.04862	1.81310	0.22529
C	0.72673	-1.76235	2.85943	0.73260	-2.08070	2.25068	0.65705	3.18589	0.93897	0.66562	3.21790	0.84817	0.84336	-3.28845	0.97933	0.84336	-3.28845	0.97933
C	1.43608	-2.58060	2.06494	1.42844	-2.85262	1.40086	1.68752	2.86576	1.73600	1.69474	2.91196	1.65283	1.62307	-3.35186	-0.11239	1.62307	-3.35186	-0.11239
C	2.36611	-2.13291	0.99521	2.39875	-2.35127	0.39140	3.00220	2.30669	1.30717	3.00264	2.32620	1.24031	2.52991	-2.26912	-0.57567	2.52991	-2.26912	-0.57567
N	3.16877	-3.09572	0.46026	3.41462	-3.19888	0.06746	3.64426	1.59843	2.27938	3.65502	1.67082	2.24292	3.38549	-2.59801	-1.58428	3.38549	-2.59801	-1.58428
O	2.41122	-0.96941	0.58757	2.29963	-1.24193	-0.14205	3.48224	2.45542	0.17941	3.46985	2.41142	0.10078	2.51272	-1.12983	-0.10274	2.51272	-1.12983	-0.10274
O	4.43029	2.72787	0.81032	3.91241	3.50586	-0.13488	4.39472	0.06476	-2.53306	4.40436	0.00556	-2.53302	4.25473	1.61185	2.53267	4.25473	1.61185	2.53267
C	3.42653	0.68147	-2.16727	4.27116	1.00464	-2.19435	3.20654	-2.53266	-0.10128	3.21904	-2.53463	-0.04013	3.41400	1.99288	-1.10215	3.41400	1.99288	-1.10215
N	4.33607	-0.20373	-2.68633	5.33091	0.16618	-2.39647	4.10529	-3.03137	0.80534	4.11660	-3.00695	0.88165	4.38294	1.70386	-2.02847	4.38294	1.70386	-2.02847
C	5.65126	-0.14956	-2.05429	6.30397	0.13521	-1.30517	5.40802	-2.37693	0.77858	5.41662	-2.34762	0.84351	5.68590	1.38546	-1.45128	5.68590	1.38546	-1.45128
C	5.41213	0.93199	-1.03461	5.61499	1.02710	-0.30972	5.23440	-1.49158	-0.42373	5.24307	-1.49197	-0.38024	5.36779	1.51869	0.01408	5.36779	1.51869	0.01408
O	6.35351	1.29985	-0.19293	6.18638	1.26519	0.84405	6.22095	-0.75304	-0.87972	6.22792	-0.76003	-0.85080	6.27482	1.27966	0.93595	6.27482	1.27966	0.93595
O	2.28669	0.85489	-2.59233	3.39108	1.28020	-2.99499	2.03562	-2.88856	-0.21131	2.05022	-2.89892	-0.14573	2.26923	2.35383	-1.36585	2.26923	2.35383	-1.36585
C	6.14757	-1.48114	-1.43635	6.67106	-1.26370	-0.75468	5.75219	-1.62333	2.09233	5.75279	-1.56260	2.14066	6.26262	-0.00078	-1.83479	6.26262	-0.00078	-1.83479
C	4.04846	-2.79968	-0.66956	4.52585	-2.75112	-0.76953	4.84303	0.80285	2.03263	4.85182	0.86405	2.02654	4.24291	-1.58858	-2.20422	4.24291	-1.58858	-2.20422
C	5.33134	-2.05239	-0.26097	5.59355	-2.00709	0.05419	4.68801	-0.62431	2.58237	4.68506	-0.55267	2.60020	5.48114	-1.24019	-1.35778	5.48114	-1.24019	-1.35778
H	-1.85603	1.04265	-0.64308	-2.18946	1.24797	-0.50267	-1.77068	-0.80837	-0.69103	-1.77509	-0.83830	-0.66392	-1.73448	0.94401	-0.07756	-1.73448	0.94401	-0.07756
H	-4.46466	0.54602	0.82646	-4.62500	0.23532	1.00104	-4.51399	0.48731	-0.80872	-4.50967	0.47031	-0.82656	-4.38561	-0.36734	0.56569	-4.38561	-0.36734	0.56569
H	-2.89959	2.34138	1.89043	-3.09576	1.82512	2.33505	-2.96109	1.01632	-2.80616	-2.95213	0.93430	-2.82993	-3.06994	0.34863	2.58279	-3.06994	0.34863	2.58279
H	-1.70929	0.88888	3.23806	-1.64871	0.23606	3.20122	-1.83667	2.85946	-1.91175	-1.84655	2.80301	-1.97613	-1.80499	-1.61259	2.80616	-1.80499	-1.61259	2.80616
H	-4.39402	1.94085	-2.69406	-4.84608	2.35344	-2.14975	-4.05583	-3.27052	-0.82051	-4.04701	-3.29181	-0.72216	-3.02277	2.41105	-1.61522	-3.02277	2.41105	-1.61522
H	-3.60945	-0.13245	-2.01344	-3.98983	0.22482	-1.97256	-3.46217	-1.81438	0.87856	-3.48453	-1.78590	0.93627	-3.84714	0.42562	-2.25813	-3.84714	0.42562	-2.25813
H	0.13214	0.18170	0.92300	-0.04031	0.14734	0.62146	-0.01237	0.97864	-0.37740	-0.00295	0.96944	-0.39929	0.16172	-0.58251	0.72359	0.16172	-0.58251	0.72359
H	0.19817	2.37715	3.02700	0.23925	1.91403	3.08518	0.19266	2.26419	-3.11990	0.19203	2.17240	-3.17937	0.01538	-0.27574	3.74495	0.01538	-0.27574	3.74495
H	6.40464	0.18918	-2.77594	7.23391	0.62269	-1.62393	6.20509	-3.10273	0.58266	6.21734	-3.07445	0.66722	6.42192	2.14747	-1.73548	6.42192	2.14747	-1.73548
H	1.32033	-3.65327	2.19308	1.30253	-3.93070	1.45407	1.55195	2.97250	2.80899	1.56340	3.05722	2.72177	1.58703	-4.25136	-0.72059	1.58703	-4.25136	-0.72059
H	0.07486	-2.25107	3.57995	0.06849	-2.61169	2.92882	-0.23208	3.55218	1.44597	-0.21687	3.61191	1.34602	0.22233	-4.16119	1.16804	0.22233	-4.16119	1.16804
H	2.40290	2.62381	2.29709	1.49189	3.52580	1.57563	2.41469	1.56910	-2.84150	2.41871	1.49951	-2.87033	2.21982	0.44828	3.46484	2.21982	0.44828	3.46484
H	1.52794	1.87699	-0.54004	2.15016	0.74070	0.41705	1.40906	-1.08142	-1.68134	1.42113	-1.13337	-1.66480	1.42572	1.76930	0.81954	1.42572	1.76930	0.81954
H	7.17194	-1.29859	-1.09943	7.54530	-1.11432	-0.11571	6.72153	-1.13346	1.95714	6.72247	-1.07513	1.99926	7.27225	-0.03831	-1.41596	7.27225	-0.03831	-1.41596
H	6.22080	-2.21388	-2.24701	7.01509	-1.86943	-1.59878	5.90377	-2.39290	2.85336	5.90099	-2.31393	2.92036	6.38503	-0.01712	-2.92296	6.38503	-0.01712	-2.92296
H	5.06105	-1.25117	0.43034	5.08659	-1.31203	0.72923	4.72775	-0.58491	3.67513	4.71107	-0.49362	3.69249	5.16653	-1.10667	-0.32041	5.16653	-1.10667	-0.32041
H	5.97254	-2.74184	0.29704	6.09808	-2.73498	0.69666	3.69112	-0.99647	2.32943	3.69011	-0.92603	2.34163	6.16168	-2.09728	-1.36864	6.16168	-2.09728	-1.36864
H	4.30070	-3.74773	-1.14934	4.96145	-3.62550	-1.25787	4.99719	0.80858	0.95504	5.01851	0.84891	0.95101	4.54886	-1.97053	-3.18050	4.54886	-1.97053	-3.18050
H	3.48208	-2.20317	-1.38416	4.11115	-2.10549	-1.54198	5.71739	1.28394	2.48538	5.72231	1.35007	2.48124	3.64063	-0.69546	-2.36837	3.64063	-0.69546	-2.36837
H	-1.80413	-1.55456	2.31454	-1.70948	-1.92896	1.76720	-2.11991	2.89947	0.65836	-2.09370	2.90284	0.58642	-1.66793	-2.88324	0.53295	-1.66793	-2.88324	0.53295
H	-3.76792	-1.79037	0.76329	-3.80147	-1.96978	0.35987	-3.95338	1.35292	1.39408	-3.94824	1.37487	1.36580	-3.57354	-2.16001	-0.92974	-3.57354	-2.16001	-0.92974
H	0.48885	0.04546	3.94818	0.66897	-0.40542	3.53681	0.08036	3.95897	-0.96090	0.07581	3.93115	-1.07061	0.42832	-2.63866	2.95887	0.42832	-2.63866	2.95887
H	1.81183	0.05388	2.77999	1.84056	-0.26556	2.23602	1.57716	3.02747	-0.96021	1.57717	3.00575	-1.04957	1.78595	-1.82229	2.18061	1.78595	-1.82229	2.18061
H	-2.75790	3.07488	-1.45615	-3.28811	3.35427	-0.71017	-2.50732	-2.36123	-2.32727	-2.51683	-2.42431	-2.27238	-2.74755	3.04946	0.69012	-2.74755	3.04946	0.69012
H	-3.88177	3.21756	-0.11417	-4.33030	3.03464	0.66751	-3.73964	-1.26729	-2.93540	-3.75089	-1.34014	-2.89483	-4.21900	2.19041	1.17483	-4.21900	2.19041	1.17483
H	-1.04328	3.76259	1.54646	-1.35713	3.41657	2.29374	-1.02140	0.45915	-4.02628	-1.01344	0.33151	-4.02806	-1.31544	1.65822	3.45279	-1.31544	1.65822	3.45279
H	-0.58665	2.83032	0.13109	-1.06620	3.01668	0.61001	-0.56451	-0.64636	-2.74534	-0.55495	-0.72775	-2.70918	-0.73601	1.94344	1.82176	-0.73601	1.94344	1.82176
O	-5.33326	-1.92574	-1.00162	-5.57467	-1.82087	-1.15841	-5.37004	-0.38611	2.13115	-5.40964	-0.29480	2.10573	-6.40335	0.65591	-0.14314	-6.40335	0.65591	-0.14314
H	-6.42750	-0.19117	-0.86378	-6.71276	-0.18119	-0.67636	-6.36796	-1.01610	0.44895	-6.38525	-1.00654	0.44384	-6.20684	1.14497	-2.13469	-6.20684	1.14497	-2.13469
H	-6.15853	2.20771	-0.19914	-6.55738	2.02004	0.37259	-6.47020	-2.75003	-1.16257	-6.05074	-1.34083	-1.98054	-5.83270	3.14210	-0.66327	-5.83270	3.14210	-0.66327
H	-5.92063	3.44295	-1.43357	-6.40222	3.49052	-0.58612	-6.04093	-1.25484	-2.00510	-5.60491	-2.92122	-2.62045	-4.52120	4.26026	-1.05630	-4.52120	4.26026	-1.05630
H	-6.73961	1.94044	-1.84835	-7.16687	2.06830	-1.28774	-5.61564	-2.81736	-2.70050	-6.46578	-2.81137	-1.08852	-5.23809	3.27294	-2.32697	-5.23809	3.27294	-2.32697
H	-6.28811	0.33589	-3.34086	-6.70492	0.76545	-3.03887	-5.96510	-3.48451	0.88773	-5.96697	-3.45144	0.99727	-5.58590	-1.16694	-2.88634	-5.58590	-1.16694	-2.88634
H	-6.97894	-1.25927	-3.06141	-7.37395	-0.86253	-2.98574	-6.78567	-2.71227	2.24066	-6.81989	-2.62615	2.29781	-7.17340	-1.11231	-2.12357	-7.17340	-1.11231	-2.12357
H	-5.28498	-1.10582	-3.56169	-5.71014	-0.60813	-3.54437	-5.04655	-3.04440	2.33539	-5.07995	-2.93734	2.43996	-5.79485	-1.75148	-1.22363	-5.79485	-1.75148	-1.22363
C	-6.48053	-2.70102	-0.68564	-6.67898	-2.69141	-0.95800	-6.58438	0.06197	2.71605	-6.63160	0.16030	2.66922	-7.81115	0.83922	-0.18143	-7.81115	0.83922	-0.18143
H	-6.12393	-3.62966	-0.23795	-6.27168	-3.66023	-0.66552	-6.32666	0.86257	3.41073	-6.38806	1.00018	3.32137	-8.12348	1.12129	0.82485	-8.12348	1.12129	0.82485
H	-7.12325	-2.18056	0.03653	-7.33340	-2.32263	-0.15716	-7.26854	0.45739	1.95381	-7.32381	0.50302	1.88892	-8.08793	1.64133	-0.87839	-8.08793	1.64133	-0.87839
H	-7.07534	-2.94694	-1.57283	-7.27597	-2.82383	-1.86748	-7.09735	-0.73120	3.27204	-7.12896	-0.61252	3.26630	-8.34158	-0.07545	-0.46935	-8.34158	-0.07545	-0.46935
H	-3.25959	-0.51638	3.82072	-3.78936	-2.13181	2.68054	-3.58886	3.75807	-0.91895	-4.27173	3.32624	0.09606	-3.98900	-2.13645	1.93758	-3.98900	-2.13645	1.93758
H	3.12062	-4.03837	0.81535	3.52164	-4.05200	0.59690	3.19390	1.50885	3.17885	3.21440	1.63026	3.15072	3.38248	-3.53655	-1.95308	3.38248	-3.53655	-1.95308
H	4.17406	-0.66926	-3.56688	5.51410	-0.25186	-3.29762	3.83770	-3.70384	1.50826	3.84945	-3.66313	1.59996	4.26059	1.92735	-3.00493	4.26059	1.92735	-3.00493
H	5.91413	1.97676	0.39814	5.63628	1.85645	1.38700	5.83000	-0.25846	-1.65691	5.83756	-0.28705	-1.64157	5.78674	1.38041	1.80331	5.78674	1.38041	1.80331

Table S11. The Cartesian coordinates of the lowest-energy conformers of (5*S*,6*R*,7*S*,8*S*,9*R*,10*R*,11*R*,13*R*,14*R*,16*S*,23*R*,29*S*)-**5C.**

5C	Conf. #1			Conf. #2			Conf. #3			Conf. #4		
	Coordinates (Angstroms)			Coordinates (Angstroms)			Coordinates (Angstroms)			Coordinates (Angstroms)		
	X	Y	Z	X	Y	Z	X	Y	Z	X	Y	Z
C	2.5587	-1.9223	0.4885	2.5634	-1.9102	0.5062	2.4758	1.7874	-0.4425	2.5152	-1.8187	0.6784
C	1.2606	-1.7778	0.1801	1.2672	-1.7633	0.1906	1.2012	1.6843	-0.0344	1.2153	-1.7041	0.3621
C	0.5853	0.9486	1.7245	0.5843	0.9817	1.6983	0.5213	-1.3771	-0.2516	0.5379	1.2701	1.3829
O	-3.6440	0.8726	2.8964	-3.5418	0.8644	2.9327	-3.2843	-1.8557	-2.2404	-3.6305	1.4251	2.6375
Cl	-2.7459	1.8132	-0.8556	-2.7600	1.8004	-0.9066	-3.1544	-1.3626	1.7521	-2.8381	1.6227	-1.2462
C	-1.0859	-2.4406	0.9301	-1.0813	-2.4175	0.9374	-1.1334	2.0329	-1.0585	-1.1148	-2.2210	1.2564
C	0.1593	-1.5396	1.1578	0.1621	-1.5135	1.1609	0.1151	1.1312	-0.8916	0.1301	-1.2911	1.2971
C	-0.3918	-0.0777	1.0839	-0.3906	-0.0537	1.0684	-0.4749	-0.2005	-0.3440	-0.4295	0.1291	0.9685
C	-2.3409	-1.5701	1.2271	-2.3374	-1.5473	1.2308	-2.3377	1.0699	-1.2906	-2.3679	-1.3127	1.4243
C	-1.7637	-0.2385	1.7848	-1.7611	-0.2078	1.7717	-1.7095	-0.3604	-1.2633	-1.7853	0.0986	1.7160
C	-2.7309	0.9637	1.7812	-2.7221	0.9936	1.7521	-2.6755	-1.5228	-0.9748	-2.7478	1.2858	1.5033
C	-3.6732	1.0757	0.5671	-3.6765	1.0831	0.5408	-3.8179	-1.2342	0.0223	-3.7233	1.1683	0.3156
C	-5.3112	-1.9923	-1.1718	-5.3170	-2.0095	-1.1528	-5.6989	2.1617	0.2109	-5.4055	-2.1672	-0.7594
C	-5.2119	-0.4308	-1.0072	-5.2300	-0.4455	-0.9952	-5.5969	0.6544	0.6472	-5.3040	-0.6043	-0.9003
C	-4.2894	-0.2713	0.2181	-4.2954	-0.2692	0.2186	-4.4676	0.1132	-0.2528	-4.3450	-0.2186	0.2442
C	-3.2079	-1.3372	-0.0152	-3.2136	-1.3356	-0.0085	-3.4286	1.2432	-0.2281	-3.2667	-1.3110	0.1829
C	-3.9969	-2.5508	-0.5369	-4.0054	-2.5574	-0.5038	-4.2751	2.5254	-0.3212	-4.0623	-2.6007	-0.0884
C	-6.5338	-2.6534	-0.5139	-6.5408	-2.6791	-0.5061	-6.7562	2.4692	-0.8628	-6.5959	-2.6818	0.0671
C	-6.5636	0.2953	-0.9738	-6.5885	0.2676	-0.9494	-6.9302	-0.1049	0.6418	-6.6550	0.1206	-0.9657
C	-7.2922	0.2066	-2.3188	-7.3195	0.1858	-2.2935	-7.8990	0.4342	1.6992	-7.4322	-0.2331	-2.2380
C	3.5965	-1.9150	-0.5515	3.6057	-1.9180	-0.5292	3.5861	2.0698	0.4714	3.5378	-2.0467	-0.3520
C	4.9438	-1.4933	-0.2031	4.9533	-1.4981	-0.1796	4.9145	1.6220	0.0792	4.9033	-1.6183	-0.0918
C	0.6371	2.2855	1.0357	0.6437	2.3061	0.9865	1.3686	-1.6063	-1.4714	0.7162	2.3329	0.3344
C	1.6661	2.8464	0.3795	1.6789	2.8555	0.3302	2.6598	-1.9651	-1.5220	1.8546	2.8837	-0.1140
C	2.9979	2.2226	0.1457	3.0123	2.2274	0.1183	3.5297	-2.2842	-0.3525	3.2301	2.5369	0.3404
N	3.7369	2.7964	-0.8507	3.7599	2.7850	-0.8812	4.8627	-2.1233	-0.5759	4.2084	2.8291	-0.5647
O	3.4227	1.2459	0.7666	3.4310	1.2604	0.7582	3.0938	-2.6954	0.7296	3.4794	2.0224	1.4352
O	3.3419	-2.1387	-1.7590	3.3554	-2.1534	-1.7355	3.4324	2.5517	1.6165	3.2624	-2.4849	-1.4929
C	5.5784	-1.2095	1.1081	5.5833	-1.2023	1.1313	5.4275	1.0816	-1.2035	5.5574	-1.1168	1.1421
N	6.7658	-0.5877	0.8247	6.7752	-0.5901	0.8462	6.6539	0.5349	-0.9237	6.7377	-0.5453	0.7383
C	7.0221	-0.3981	-0.5975	7.0374	-0.4151	-0.5767	7.0849	0.7247	0.4576	7.0185	-0.7011	-0.6876
C	5.8087	-1.0729	-1.1766	5.8243	-1.0921	-1.1538	5.8996	1.4622	1.0156	5.7851	-1.4440	-1.1250
O	5.6288	-1.1615	-2.4768	5.6498	-1.1946	-2.4538	5.8417	1.8129	2.2797	5.5925	-1.7740	-2.3811
O	5.1777	-1.4920	2.2350	5.1759	-1.4697	2.2594	4.9042	1.1033	-2.3153	5.1693	-1.1974	2.3041
C	7.2190	1.0856	-1.0166	7.2392	1.0642	-1.0091	7.4144	-0.5737	1.2256	7.2345	0.6198	-1.4700
C	4.8804	2.0925	-1.4385	4.9043	2.0693	-1.4527	5.8857	-2.5617	0.3709	5.5821	2.3492	-0.4352
C	6.1391	2.0818	-0.5557	6.1585	2.0665	-0.5636	6.2756	-1.5781	1.4955	5.9956	1.5140	-1.6604
H	-2.5642	-0.9712	-0.8248	-2.5774	-0.9807	-0.8290	-2.9539	1.2170	0.7604	-2.6446	-1.0996	-0.6957
H	-4.8585	-0.5449	1.1187	-4.8544	-0.5310	1.1291	-4.8562	0.0174	-1.2773	-4.8859	-0.3161	1.1969
H	-2.9669	-2.0470	1.9881	-2.9566	-2.0172	2.0019	-2.7888	1.2515	-2.2712	-2.9708	-1.6456	2.2751
H	-1.5220	-0.4057	2.8436	-1.5307	-0.3523	2.8338	-1.3308	-0.5716	-2.2695	-1.5224	0.1310	2.7825
H	-5.3385	-2.2296	-2.2385	-5.3316	-2.2503	-2.2190	-5.9329	2.7658	1.0914	-5.4805	-2.6043	-1.7585
H	-4.6627	-0.0472	-1.8753	-4.6942	-0.0608	-1.8710	-5.2230	0.6389	1.6780	-4.7821	-0.3962	-1.8420
H	-0.5427	0.1755	0.0303	-0.5401	0.1864	0.0115	-0.8244	-0.0155	0.6770	-0.6106	0.1793	-0.1095
H	0.5398	-1.6920	2.1737	0.5379	-1.6544	2.1801	0.5326	0.9270	-1.8832	0.5266	-1.2579	2.3175
H	7.9174	-0.9518	-0.9054	7.9325	-0.9739	-0.8759	7.9680	1.3740	0.4938	7.9032	-1.3324	-0.8312
H	1.5082	3.8173	-0.0823	1.5253	3.8184	-0.1496	3.1348	-2.0368	-2.4965	1.7881	3.6141	-0.9158
H	-0.2825	2.8662	1.0561	-0.2757	2.8875	0.9881	0.8818	-1.4429	-2.4308	-0.2009	2.6794	-0.1377
H	0.9815	-1.7531	-0.8723	0.9931	-1.7475	-0.8633	0.9497	1.9333	0.9954	0.9223	-1.8643	-0.6745
H	2.9028	-1.8986	1.5157	2.9031	-1.8780	1.5347	2.7551	1.5047	-1.4506	2.8739	-1.6265	1.6832
H	8.1817	1.3927	-0.5988	8.2003	1.3736	-0.5893	8.2342	-1.0710	0.6970	8.0423	1.1707	-0.9784
H	7.3391	1.1097	-2.1050	7.3650	1.0775	-2.0970	7.8246	-0.2570	2.1892	7.6135	0.3361	-2.4556
H	5.8518	1.8547	0.4713	5.8659	1.8508	0.4644	6.6157	-2.1981	2.3311	6.1942	2.1912	-2.4973
H	6.5741	3.0857	-0.5523	6.5950	3.0698	-0.5687	5.3779	-1.0684	1.8560	5.1430	0.9019	-1.9683
H	5.0979	2.5748	-2.3933	5.1285	2.5375	-2.4129	6.7699	-2.8377	-0.2088	5.6179	1.7610	0.4783
H	4.5645	1.0715	-1.6583	4.5863	1.0464	-1.6598	5.5053	-3.4717	0.8386	6.2668	3.1949	-0.3100
H	-2.1675	1.8967	1.8709	-2.1545	1.9256	1.8209	-2.1138	-2.3902	-0.6160	-2.1758	2.2123	1.3924
H	-4.4331	1.8200	0.7747	-4.4396	1.8321	0.7273	-4.5451	-2.0395	-0.0083	-4.4788	1.9408	0.4000
H	1.5872	0.5281	1.7443	1.5852	0.5599	1.7335	-0.0416	-2.2954	-0.0399	1.5116	0.8667	1.6548
H	0.2816	1.1127	2.7669	0.2721	1.1650	2.7349	1.1665	-1.2370	0.6160	0.1511	1.7498	2.2926
H	-3.4205	-3.1444	-1.2502	-3.4307	-3.1707	-1.2016	-3.8314	3.3541	0.2353	-3.5059	-3.3054	-0.7105
H	-4.2473	-3.2146	0.2978	-4.2598	-3.1994	0.3467	-4.3455	2.8475	-1.3657	-4.2731	-3.1119	0.8570
H	-1.0362	-3.3266	1.5663	-1.0303	-3.3012	1.5766	-0.9967	2.7417	-1.8775	-1.0464	-2.9826	2.0356
H	-1.1102	-2.7981	-0.1033	-1.1051	-2.7785	-0.0949	-1.2923	2.6222	-0.1502	-1.1595	-2.7526	0.3014
O	-6.3233	1.6713	-0.6282	-6.3613	1.6418	-0.5871	-6.6498	-1.4952	0.8846	-6.4029	1.5363	-0.9137
H	-7.1908	-0.1375	-0.1831	-7.2094	-0.1799	-0.1620	-7.3889	-0.0308	-0.3533	-7.2525	-0.1401	-0.0819
H	-6.4558	-3.7402	-0.6130	-6.4523	-3.7654	-0.6020	-6.6828	3.5212	-1.1542	-6.5210	-3.7681	0.1741
H	-7.4802	-2.3533	-0.9693	-7.4850	-2.3884	-0.9718	-7.7785	2.2984	-0.5182	-7.5623	-2.4672	-0.3943
H	-6.5868	-2.4278	0.5560	-6.6064	-2.4518	0.5627	-6.5996	1.8704	-1.7657	-6.6012	-2.2559	1.0755
H	-7.4719	-0.8303	-2.6088	-7.4781	-0.8502	-2.5987	-8.1107	1.4937	1.5437	-7.6207	-1.3061	-2.3061
H	-8.2612	0.7092	-2.2817	-8.2986	0.6674	-2.2468	-8.8528	-0.0974	1.6752	-8.4001	0.2721	-2.2678
H	-6.6878	0.6793	-3.0984	-6.7267	0.6823	-3.0671	-7.4628	0.3157	2.6953	-6.8577	0.0693	-3.1183
C	-7.4616	2.3631	-0.1348	-7.5103	2.3232	-0.1041	-7.6930	-2.3835	0.5094	-7.5248	2.3234	-0.5403
H	-8.2361	2.4910	-0.8997	-7.9649	1.7848	0.7378	-8.5855	-2.2664	1.1347	-8.3184	2.3036	-1.2960
H	-7.8993	1.8412	0.7263	-7.1780	3.3037	0.2395	-7.9771	-2.2389	-0.5412	-7.9420	1.9881	0.4183
H	-7.1215	3.3494	0.1837	-8.2690	2.4647	-0.8823	-7.3096	-3.3967	0.6373	-7.1718	3.3501	-0.4337
H	-3.1260	0.8481	3.7090	-4.0457	1.6787	3.0483	-3.7752	-2.6797	-2.1387	-3.0915	1.5446	3.4277
H	3.3125	3.5333	-1.3935	3.3395	3.5132	-1.4388	5.1587	-1.7553	-1.4669	3.9285	3.2231	-1.4516
H	7.4381	-0.3664	1.5434	7.4433	-0.3595	1.5658	7.2998	0.2982	-1.6625	7.4571	-0.3078	1.4055
H	4.7340	-1.5997	-2.5777	4.7536	-1.6303	-2.5536	4.9390	2.2283	2.3905	4.6765	-2.1778	-2.4008

Table S12. Conformers and Boltzmann distributions of the optimized **6**.

Comp. **6**	Conf. No.	BOLTZMANN POPULATION,%	TOTAL GIBBS FREE ENERGY, KCAL/MOL
7R,8R,23S-**6A**	#1	61.2079	446.0684
	#2	30.1109	445.8091
7S,8S,23R-**6B**	#1	62.8202	446.0684
	#2	30.9041	445.8091
7R,8R,23R-**6C**	#1	52.9389	445.1932
	#2	28.1516	446.0343
	#3	10.1296	446.6914

Table S13. The Cartesian coordinates of the lowest-energy conformers of (5*S*,6*R*,7*R*,8*R*,9*R*,10*R*,11*R*,13*R*,14*R*,16*S*,23*S,*29*S*)-**6A.**

6A	Conf. #1			Conf. #2		
	Coordinates (Angstroms)			Coordinates (Angstroms)		
	X	Y	Z	X	Y	Z
C	-2.3241	-2.1282	-0.3019	-2.2839	-1.9386	-0.7871
C	-1.6084	-2.1294	-1.4370	-1.6170	-1.5686	-1.8925
C	-0.6314	0.6749	-2.5392	-0.5172	1.3656	-2.4230
H	1.5399	2.0797	-0.3513	1.5742	2.1517	0.1726
H	3.5110	1.3963	1.3077	3.4840	1.0160	1.6565
C	0.8600	-2.2633	-0.5972	0.8458	-1.9725	-1.1324
C	-0.1789	-1.6919	-1.5799	-0.1722	-1.1732	-1.9625
C	-0.0190	-0.1464	-1.3832	0.0252	0.2944	-1.4444
C	2.0876	-1.3757	-0.8584	2.0931	-1.0759	-1.1713
C	1.4995	0.0535	-1.1244	1.5365	0.3867	-1.0910
C	1.8727	1.0870	-0.0582	1.8844	1.1106	0.2133
C	3.3917	1.1625	0.2448	3.3949	1.0803	0.5671
C	5.5765	-2.0401	0.5223	5.5598	-2.0981	0.0182
C	5.3867	-0.4970	0.7612	5.3761	-0.6728	0.6554
C	4.1280	-0.1535	-0.0668	4.1300	-0.1138	-0.0678
C	3.2027	-1.3618	0.1857	3.1927	-1.3366	-0.1430
C	4.1450	-2.5786	0.2103	4.1235	-2.5344	-0.4093
C	6.5440	-2.4208	-0.6111	6.5149	-2.1634	-1.1858
C	6.6651	0.3229	0.5278	6.6618	0.1683	0.6610
C	7.7351	-0.0011	1.5734	7.7244	-0.4400	1.5795
C	-3.7891	-2.2213	-0.3571	-3.7532	-1.9616	-0.7877
C	-4.5689	-1.5371	0.6639	-4.4528	-1.6779	0.4579
C	-0.7174	2.1611	-2.2903	-0.7317	2.6994	-1.7584
C	-1.6551	2.8365	-1.6048	-1.8704	3.1770	-1.2340
C	-2.7904	2.2155	-0.8723	-3.1932	2.4833	-1.2346
N	-3.6926	3.0798	-0.3262	-4.0074	2.8353	-0.2004
O	-2.9151	0.9960	-0.7322	-3.5354	1.6576	-2.0867
O	-4.4023	-2.7305	-1.3242	-4.4318	-2.0667	-1.8336
C	-4.1661	-0.9074	1.9434	-3.9813	-1.6292	1.8649
N	-5.2279	-0.1348	2.3374	-5.0329	-1.1418	2.5953
C	-6.3822	-0.2042	1.4459	-6.1872	-0.7491	1.7961
C	-5.8617	-1.1668	0.4122	-5.7425	-1.2203	0.4392
O	-6.5854	-1.5079	-0.6316	-6.5297	-1.1291	-0.6095
O	-3.1172	-1.0402	2.5706	-2.8941	-1.9716	2.3236
C	-6.8535	1.1513	0.8597	-6.5126	0.7666	1.8816
C	-4.7221	2.6056	0.6007	-5.2692	2.1611	0.0914
C	-5.8900	1.8833	-0.0944	-5.3421	1.7158	1.5602
H	2.7607	-1.2427	1.1811	2.7343	-1.4651	0.8440
H	4.3904	-0.1374	-1.1341	4.4059	0.1764	-1.0913
H	2.5367	-1.7118	-1.8015	2.5459	-1.1862	-2.1647
H	1.9677	0.4545	-2.0321	2.0432	0.9919	-1.8527
H	5.9511	-2.4928	1.4440	5.9432	-2.7780	0.7833
H	5.1132	-0.3647	1.8170	5.0897	-0.8224	1.7055
H	-0.5725	0.1064	-0.4796	-0.5518	0.3663	-0.5203
H	0.1505	-1.9401	-2.5961	0.1414	-1.2056	-3.0128
H	-7.2344	-0.6576	1.9669	-7.0795	-1.3080	2.0996
H	-1.5755	3.9187	-1.5518	-1.8388	4.1330	-0.7184
H	0.0698	2.7629	-2.7392	0.1510	3.3253	-1.6433
H	-2.1435	-2.3517	-2.3567	-2.2032	-1.4250	-2.7964
H	-1.8952	-1.8476	0.6525	-1.8003	-2.0477	0.1765
H	-7.7852	0.9454	0.3251	-7.3710	0.9648	1.2322
H	-7.1241	1.7956	1.7030	-6.8574	0.9452	2.9030
H	-5.4819	1.1803	-0.8230	-5.4315	2.6051	2.1914
H	-6.4684	2.6204	-0.6603	-4.3952	1.2434	1.8367
H	-5.0914	3.4744	1.1494	-5.3296	1.3144	-0.5904
H	-4.2461	1.9376	1.3187	-6.1118	2.8228	-0.1394
Cl	0.9557	0.8176	1.5423	0.9105	0.4630	1.6640
O	3.8904	2.2556	-0.5327	3.9221	2.3350	0.1268
H	-1.6376	0.2899	-2.7143	-1.4639	1.0182	-2.8320
H	-0.0430	0.5064	-3.4477	0.1888	1.4814	-3.2530
H	3.8245	-3.3256	0.9405	3.7954	-3.4276	0.1273
H	4.1434	-3.0726	-0.7669	4.1167	-2.7869	-1.4746
H	1.0583	-3.3239	-0.7708	1.0252	-2.9688	-1.5439
H	0.5138	-2.1495	0.4350	0.4966	-2.0918	-0.1022
O	6.3272	1.7311	0.5710	6.3328	1.5125	1.0906
H	7.0550	0.1233	-0.4781	7.0553	0.2508	-0.3598
H	6.5303	-3.5058	-0.7499	6.4986	-3.1728	-1.6072
H	7.5780	-2.1337	-0.4063	7.5512	-1.9395	-0.9226
H	6.2490	-1.9672	-1.5627	6.2112	-1.4742	-1.9801
H	7.9441	-1.0716	1.5957	7.9306	-1.4748	1.3022
H	8.6768	0.5099	1.3649	8.6686	0.1049	1.5267
H	7.3883	0.2989	2.5665	7.3731	-0.4285	2.6154
C	7.3908	2.6142	0.2092	7.4047	2.4529	0.9946
H	8.1587	2.6627	0.9856	8.1658	2.2785	1.7594
H	7.8515	2.3061	-0.7369	7.8718	2.4150	0.0034
H	6.9514	3.6049	0.0882	6.9720	3.4418	1.1496
H	-3.5439	4.0727	-0.4220	-3.6651	3.5114	0.4674
H	-5.2750	0.2668	3.2618	-4.9691	-0.9890	3.5904
H	-5.9872	-2.1015	-1.1709	-5.9885	-1.4900	-1.3683
H	4.8423	2.2739	-0.3206	4.8686	2.2824	0.3577

Table S14. The Cartesian coordinates of the lowest-energy conformers of (5*R*,6*S*,7*S*,8*S*,9*S*,10*S*,11*S*,13*S*,14*S*,16*R*,23*R*,29*R*)-**6B.**

6B	Conf. #1			Conf. #2		
	Coordinates (Angstroms)			Coordinates (Angstroms)		
	X	Y	Z	X	Y	Z
C	2.3241	-2.1282	-0.3019	2.2839	-1.9386	-0.7871
C	1.6084	-2.1294	-1.4370	1.6170	-1.5686	-1.8925
C	0.6314	0.6749	-2.5392	0.5172	1.3656	-2.4230
H	-1.5399	2.0797	-0.3513	-1.5742	2.1517	0.1726
H	-3.5110	1.3963	1.3077	-3.4840	1.0160	1.6565
C	-0.8600	-2.2633	-0.5972	-0.8458	-1.9725	-1.1324
C	0.1789	-1.6919	-1.5799	0.1722	-1.1732	-1.9625
C	0.0190	-0.1464	-1.3832	-0.0252	0.2944	-1.4444
C	-2.0876	-1.3757	-0.8584	-2.0931	-1.0759	-1.1713
C	-1.4995	0.0535	-1.1244	-1.5365	0.3867	-1.0910
C	-1.8727	1.0870	-0.0582	-1.8844	1.1106	0.2133
C	-3.3917	1.1625	0.2448	-3.3949	1.0803	0.5671
C	-5.5765	-2.0401	0.5223	-5.5598	-2.0981	0.0182
C	-5.3867	-0.4970	0.7612	-5.3761	-0.6728	0.6554
C	-4.1280	-0.1535	-0.0668	-4.1300	-0.1138	-0.0678
C	-3.2027	-1.3618	0.1857	-3.1927	-1.3366	-0.1430
C	-4.1450	-2.5786	0.2103	-4.1235	-2.5344	-0.4093
C	-6.5440	-2.4208	-0.6111	-6.5149	-2.1634	-1.1858
C	-6.6651	0.3229	0.5278	-6.6618	0.1683	0.6610
C	-7.7351	-0.0011	1.5734	-7.7244	-0.4400	1.5795
C	3.7891	-2.2213	-0.3571	3.7532	-1.9616	-0.7877
C	4.5689	-1.5371	0.6639	4.4528	-1.6779	0.4579
C	0.7174	2.1611	-2.2903	0.7317	2.6994	-1.7584
C	1.6551	2.8365	-1.6048	1.8704	3.1770	-1.2340
C	2.7904	2.2155	-0.8723	3.1932	2.4833	-1.2346
N	3.6926	3.0798	-0.3262	4.0074	2.8353	-0.2004
O	2.9151	0.9960	-0.7322	3.5354	1.6576	-2.0867
O	4.4023	-2.7305	-1.3242	4.4318	-2.0667	-1.8336
C	4.1661	-0.9074	1.9434	3.9813	-1.6292	1.8649
N	5.2279	-0.1348	2.3374	5.0329	-1.1418	2.5953
C	6.3822	-0.2042	1.4459	6.1872	-0.7491	1.7961
C	5.8617	-1.1668	0.4122	5.7425	-1.2203	0.4392
O	6.5854	-1.5079	-0.6316	6.5297	-1.1291	-0.6095
O	3.1172	-1.0402	2.5706	2.8941	-1.9716	2.3236
C	6.8535	1.1513	0.8597	6.5126	0.7666	1.8816
C	4.7221	2.6056	0.6007	5.2692	2.1611	0.0914
C	5.8900	1.8833	-0.0944	5.3421	1.7158	1.5602
H	-2.7607	-1.2427	1.1811	-2.7343	-1.4651	0.8440
H	-4.3904	-0.1374	-1.1341	-4.4059	0.1764	-1.0913
H	-2.5367	-1.7118	-1.8015	-2.5459	-1.1862	-2.1647
H	-1.9677	0.4545	-2.0321	-2.0432	0.9919	-1.8527
H	-5.9511	-2.4928	1.4440	-5.9432	-2.7780	0.7833
H	-5.1132	-0.3647	1.8170	-5.0897	-0.8224	1.7055
H	0.5725	0.1064	-0.4796	0.5518	0.3663	-0.5203
H	-0.1505	-1.9401	-2.5961	-0.1414	-1.2056	-3.0128
H	7.2344	-0.6576	1.9669	7.0795	-1.3080	2.0996
H	1.5755	3.9187	-1.5518	1.8388	4.1330	-0.7184
H	-0.0698	2.7629	-2.7392	-0.1510	3.3253	-1.6433
H	2.1435	-2.3517	-2.3567	2.2032	-1.4250	-2.7964
H	1.8952	-1.8476	0.6525	1.8003	-2.0477	0.1765
H	7.7852	0.9454	0.3251	7.3710	0.9648	1.2322
H	7.1241	1.7956	1.7030	6.8574	0.9452	2.9030
H	5.4819	1.1803	-0.8230	5.4315	2.6051	2.1914
H	6.4684	2.6204	-0.6603	4.3952	1.2434	1.8367
H	5.0914	3.4744	1.1494	5.3296	1.3144	-0.5904
H	4.2461	1.9376	1.3187	6.1118	2.8228	-0.1394
Cl	-0.9557	0.8176	1.5423	-0.9105	0.4630	1.6640
O	-3.8904	2.2556	-0.5327	-3.9221	2.3350	0.1268
H	0.0430	0.5064	-3.4477	-0.1888	1.4814	-3.2530
H	1.6376	0.2899	-2.7143	1.4639	1.0182	-2.8320
H	-3.8245	-3.3256	0.9405	-3.7954	-3.4276	0.1273
H	-4.1434	-3.0726	-0.7669	-4.1167	-2.7869	-1.4746
H	-1.0583	-3.3239	-0.7708	-1.0252	-2.9688	-1.5439
H	-0.5138	-2.1495	0.4350	-0.4966	-2.0918	-0.1022
O	-6.3272	1.7311	0.5710	-6.3328	1.5125	1.0906
H	-7.0550	0.1233	-0.4781	-7.0553	0.2508	-0.3598
H	-6.5303	-3.5058	-0.7499	-6.4986	-3.1728	-1.6072
H	-7.5780	-2.1337	-0.4063	-7.5512	-1.9395	-0.9226
H	-6.2490	-1.9672	-1.5627	-6.2112	-1.4742	-1.9801
H	-7.9441	-1.0716	1.5957	-7.9306	-1.4748	1.3022
H	-8.6768	0.5099	1.3649	-8.6686	0.1049	1.5267
H	-7.3883	0.2989	2.5665	-7.3731	-0.4285	2.6154
C	-7.3908	2.6142	0.2092	-7.4047	2.4529	0.9946
H	-6.9514	3.6049	0.0882	-6.9720	3.4418	1.1496
H	-7.8515	2.3061	-0.7369	-7.8718	2.4150	0.0034
H	-8.1587	2.6627	0.9856	-8.1658	2.2785	1.7594
H	3.5439	4.0727	-0.4220	3.6651	3.5114	0.4674
H	5.2750	0.2668	3.2618	4.9691	-0.9890	3.5904
H	5.9872	-2.1015	-1.1709	5.9885	-1.4900	-1.3683
H	-4.8423	2.2739	-0.3206	-4.8686	2.2824	0.3577

Table S15. The Cartesian coordinates of the lowest-energy conformers of (5*S*,6*R*,7*R*,8*R*,9*R*,10*R*,11*R*,13*R*,14*R*,16*S*,23*R*,29*S*)-**6C.**

6C	Conf. #1			Conf. #2			Conf. #3		
	Coordinates (Angstroms)			Coordinates (Angstroms)			Coordinates (Angstroms)		
	X	Y	Z	X	Y	Z	X	Y	Z
C	-2.6322	-2.0541	-0.0109	-2.5977	-1.9752	-0.3055	-2.5513	-1.7543	0.0356
C	-1.4481	-1.7637	0.5512	-1.3819	-1.8037	0.2383	-1.4139	-1.5978	0.7311
C	-0.7413	0.7705	-1.2640	-0.6348	1.0580	-1.1457	-0.6516	1.4696	0.3600
H	1.9994	2.1713	0.3267	2.0463	1.9907	0.9845	2.4019	2.1630	1.3243
H	4.3347	1.3755	1.3646	4.3522	0.8808	1.8004	4.7967	1.0627	1.6735
C	1.0961	-2.1097	0.4173	1.1350	-2.1231	-0.0820	1.1172	-1.9582	0.3221
C	-0.1825	-1.5066	-0.1922	-0.1516	-1.3835	-0.4932	-0.1522	-1.1088	0.1074
C	0.1117	0.0356	-0.2072	0.1588	0.1295	-0.1982	0.2832	0.2781	0.6694
C	2.2195	-1.3054	-0.2554	2.2579	-1.1828	-0.5449	2.2228	-1.0246	-0.2016
C	1.6537	0.1486	-0.3757	1.7096	0.2556	-0.2752	1.7603	0.4160	0.2089
C	2.3582	1.1645	0.5267	2.3934	0.9641	0.8968	2.6992	1.1255	1.1888
C	3.9029	1.1712	0.3792	3.9421	0.9974	0.7916	4.1903	1.1088	0.7634
C	5.9315	-2.1173	-0.0254	5.9690	-2.0150	-0.5840	5.8489	-2.0823	-0.7143
C	5.8921	-0.5712	0.2577	5.9337	-0.6291	0.1557	5.9787	-0.6548	-0.0648
C	4.4546	-0.1682	-0.1422	4.4966	-0.1232	-0.1055	4.5430	-0.0884	-0.1391
C	3.6027	-1.3373	0.3943	3.6442	-1.3978	0.0643	3.6664	-1.3102	0.2056
C	4.4567	-2.5957	0.1524	4.4991	-2.5351	-0.5275	4.3600	-2.4944	-0.4898
C	6.4561	-2.5196	-1.4142	6.4616	-1.9671	-2.0406	6.2009	-2.1670	-2.2090
C	7.0705	0.1953	-0.3627	7.1142	0.2866	-0.2020	7.1274	0.1839	-0.6460
C	8.3986	-0.1975	0.2892	8.4441	-0.3001	0.2772	8.4906	-0.4111	-0.2841
C	-3.8775	-1.9036	0.7507	-3.7922	-2.1076	0.5417	-3.8587	-1.9535	0.6667
C	-5.1010	-1.5815	0.0270	-5.0715	-1.6587	0.0134	-5.0377	-1.5716	-0.0970
C	-0.8136	2.2664	-1.1019	-0.8282	2.4478	-0.6058	-1.1354	1.5514	-1.0600
C	-1.8091	2.9681	-0.5405	-1.9787	3.1282	-0.4991	-2.3620	1.8763	-1.4937
C	-3.0085	2.3498	0.0987	-3.3090	2.6292	-0.9586	-3.5086	2.3094	-0.6413
N	-4.1699	3.0533	-0.0261	-4.3656	3.0446	-0.2044	-4.7371	2.0793	-1.1798
O	-2.9552	1.2802	0.7098	-3.4479	1.9004	-1.9453	-3.3706	2.8665	0.4543
O	-3.9156	-1.8965	2.0016	-3.7355	-2.4503	1.7445	-4.0058	-2.3012	1.8600
C	-5.4386	-1.6451	-1.4163	-5.4856	-1.2715	-1.3577	-5.2008	-1.1936	-1.5222
N	-6.6749	-1.0672	-1.5385	-6.6715	-0.5958	-1.2185	-6.4471	-0.6315	-1.6272
C	-7.2069	-0.5187	-0.2969	-7.1803	-0.5615	0.1508	-7.2145	-0.6572	-0.3862
C	-6.1432	-0.9686	0.6670	-6.0884	-1.3143	0.8628	-6.2230	-1.3116	0.5354
O	-6.2436	-0.7346	1.9571	-6.1190	-1.4981	2.1629	-6.4919	-1.5089	1.8056
O	-4.7867	-2.1290	-2.3395	-4.9253	-1.5118	-2.4235	-4.4149	-1.3426	-2.4553
C	-7.4440	1.0141	-0.3475	-7.4082	0.8546	0.7363	-7.6948	0.7224	0.1134
C	-5.4564	2.5027	0.3921	-5.7121	2.4966	-0.3529	-5.9697	2.6036	-0.5947
C	-6.2266	1.8592	-0.7760	-6.1509	1.7292	0.9070	-6.6342	1.7656	0.5201
H	3.4903	-1.1964	1.4752	3.5309	-1.5746	1.1401	3.7124	-1.4642	1.2893
H	4.3749	-0.1532	-1.2383	4.4181	0.2173	-1.1472	4.3242	0.2085	-1.1747
H	2.3314	-1.6870	-1.2782	2.3539	-1.2865	-1.6330	2.1877	-1.0677	-1.2974
H	1.8733	0.5222	-1.3834	1.9697	0.8920	-1.1295	1.8013	1.0585	-0.6795
H	6.5704	-2.5947	0.7220	6.6258	-2.6927	-0.0327	6.5090	-2.7696	-0.1789
H	5.9646	-0.4394	1.3460	6.0058	-0.8347	1.2327	6.2017	-0.8035	1.0005
H	-0.1681	0.4249	0.7735	-0.1593	0.3326	0.8282	0.2910	0.1769	1.7579
H	-0.2848	-1.8520	-1.2278	-0.3041	-1.4995	-1.5720	-0.3164	-1.0196	-0.9723
H	-8.1549	-1.0020	-0.0339	-8.1219	-1.1187	0.2210	-8.0929	-1.3047	-0.4956
H	-1.7692	4.0538	-0.5580	-1.9701	4.1009	-0.0147	-2.5594	1.8293	-2.5610
H	0.0097	2.8379	-1.5254	0.0632	2.9399	-0.2211	-0.4097	1.2899	-1.8282
H	-1.4249	-1.5626	1.6205	-1.2918	-1.8752	1.3212	-1.4220	-1.7414	1.8104
H	-2.7315	-2.2078	-1.0795	-2.7658	-1.8667	-1.3709	-2.5692	-1.5748	-1.0329
H	-8.2685	1.1704	-1.0476	-8.1509	1.3602	0.1112	-8.3547	1.1401	-0.6540
H	-7.8170	1.3280	0.6326	-7.8784	0.7111	1.7128	-8.3298	0.5241	0.9821
H	-5.5336	1.2453	-1.3576	-6.3437	2.4514	1.7066	-7.1514	2.4810	1.1672
H	-6.5621	2.6527	-1.4497	-5.3113	1.1183	1.2513	-5.8574	1.3154	1.1443
H	-6.0515	3.3027	0.8404	-5.6854	1.8432	-1.2217	-6.6750	2.7667	-1.4130
H	-5.2456	1.7694	1.1693	-6.4192	3.3057	-0.5632	-5.7227	3.5816	-0.1774
Cl	1.9204	0.9372	2.3244	1.9044	0.2399	2.5429	2.5688	0.4459	2.9210
O	4.2039	2.2587	-0.4997	4.2684	2.2989	0.2981	4.4236	2.3603	0.1105
H	-1.7499	0.3590	-1.2182	-1.6083	0.6196	-1.3596	-0.1130	2.3959	0.6025
H	-0.3358	0.5479	-2.2582	-0.1056	1.1137	-2.1066	-1.5083	1.4501	1.0334
H	4.3598	-3.3126	0.9710	4.4169	-3.4491	0.0652	4.2764	-3.4149	0.0927
H	4.1233	-3.1082	-0.7558	4.1547	-2.7826	-1.5366	3.8841	-2.6890	-1.4567
H	1.1758	-3.1846	0.2368	1.2003	-3.1172	-0.5314	1.0781	-2.9110	-0.2110
H	1.1062	-1.9493	1.5009	1.1671	-2.2449	1.0056	1.2547	-2.1714	1.3880
O	6.8333	1.6162	-0.2070	6.8898	1.5853	0.4004	7.0151	1.5369	-0.1402
H	7.1130	-0.0009	-1.4412	7.1462	0.4431	-1.2875	7.0261	0.2458	-1.7366
H	6.3540	-3.6013	-1.5409	6.3616	-2.9592	-2.4904	5.9881	-3.1760	-2.5743
H	7.5106	-2.2748	-1.5609	7.5111	-1.6765	-2.1275	7.2547	-1.9644	-2.4135
H	5.8835	-2.0424	-2.2158	5.8682	-1.2741	-2.6453	5.6006	-1.4720	-2.8046
H	8.5550	-1.2757	0.2322	8.5965	-1.3006	-0.1299	8.5522	-1.4551	-0.5946
H	9.2491	0.2806	-0.1998	9.2934	0.3101	-0.0352	9.3103	0.1218	-0.7693
H	8.3959	0.0912	1.3442	8.4494	-0.3714	1.3688	8.6398	-0.3688	0.7988
C	7.7672	2.4578	-0.8868	7.8272	2.5948	0.0191	7.9301	2.4700	-0.7181
H	8.7449	2.4553	-0.3980	7.9403	2.6328	-1.0708	8.9525	2.3041	-0.3687
H	7.8842	2.1486	-1.9322	7.4274	3.5460	0.3718	7.9082	2.4139	-1.8129
H	7.3619	3.4696	-0.8552	8.8056	2.4326	0.4785	7.6091	3.4635	-0.4034
H	-4.1686	3.8801	-0.6062	-4.1703	3.6050	0.6133	-4.7910	1.5964	-2.0635
H	-7.1371	-0.9735	-2.4302	-7.2579	-0.4018	-2.0169	-6.8819	-0.4901	-2.5274
H	-5.4148	-1.1311	2.3519	-5.2551	-1.9535	2.3780	-5.6566	-1.9016	2.1885
H	5.1757	2.2293	-0.5814	5.2419	2.2848	0.2273	5.3661	2.3189	-0.1359

Table S16. Cytotoxicity of **3**–**6** against solid cancer cell lines.

ACHN

	3		4		5		6			Adriamycin	
Conc.(uM)	Mean	SD	Mean	SD	Mean	SD	Mean	SD		Mean	SD
30	−19.23	5.28	−13.44	4.42	−17.93	4.23	20.38	6.94	3	7.64	6.65
10	−3.40	7.00	21.63	7.53	23.94	9.91	75.34	2.47	1	23.78	3.17
3	79.33	7.98	75.11	7.38	81.27	6.73	85.13	7.18	0.3	32.55	6.50
1	86.71	8.40	90.61	3.32	94.76	4.66	95.96	2.48	0.1	87.70	3.05
0.3	98.46	3.55	99.41	2.06	99.34	3.07	97.96	3.35	0.03	98.23	2.71
GI50	3.549		5.000		5.701		15.950		GI50	0.255	

HCT-15

	3		4		5		6			Adriamycin	
Conc.(uM)	Mean	SD	Mean	SD	Mean	SD	Mean	SD		Mean	SD
30	−10.79	4.83	−12.71	5.58	−16.89	3.24	24.22	9.94	3	6.86	5.28
10	−2.73	7.72	22.07	7.27	28.71	1.02	72.24	6.55	1	22.96	6.93
3	74.17	5.85	73.47	4.49	79.23	4.92	84.10	8.19	0.3	38.29	5.73
1	88.27	5.92	92.90	5.88	90.21	3.49	89.18	4.28	0.1	84.95	3.25
0.3	96.38	5.86	97.58	4.78	94.22	3.59	95.94	2.67	0.03	97.66	4.44
GI50	3.469		4.930		5.790		15.540		GI_{50}	0.289	

MDA-MB-231

	3		4		5		6			Adriamycin	
Conc.(uM)	Mean	SD	Mean	SD	Mean	SD	Mean	SD		Mean	SD
30	−18.10	6.34	−12.05	5.63	−15.88	2.03	24.56	8.68	3	6.72	6.90
10	−9.16	5.02	39.96	6.55	24.42	4.14	77.89	4.01	1	24.41	6.09
3	66.33	5.03	74.94	5.82	73.15	5.07	79.47	5.73	0.3	39.62	3.38
1	89.73	4.10	88.89	4.29	89.77	3.57	91.13	6.18	0.1	90.10	9.87
0.3	97.96	4.33	95.44	7.14	96.35	3.47	98.42	4.02	0.03	94.48	2.92
GI_{50}	3.280		6.242		4.953		16.690		GI_{50}	0.298	

NUGC-3

	3		4		5		6			Adriamycin	
Conc.(uM)	Mean	SD	Mean	SD	Mean	SD	Mean	SD		Mean	SD
30	−23.69	4.30	−17.51	6.08	−27.20	1.60	13.72	2.49	3	4.71	6.87
10	−20.47	4.08	12.42	2.84	11.34	4.83	63.27	3.86	1	17.83	1.93
3	46.02	2.94	65.03	4.46	76.35	6.07	80.50	3.51	0.3	36.91	5.34
1	87.23	7.66	84.29	3.50	86.84	1.02	89.75	3.67	0.1	79.78	6.39
0.3	98.09	2.09	97.18	4.26	99.76	3.80	98.77	4.93	0.03	91.47	3.35
GI_{50}	2.815		3.779		4.493		11.450		GI_{50}	0.236	

PC-3

	3		4		5		6			Adriamycin	
Conc.(uM)	Mean	SD	Mean	SD	Mean	SD	Mean	SD		Mean	SD
30	−16.11	3.78	−14.04	4.54	−18.22	6.83	20.86	6.46	3	7.82	4.29
10	−11.65	7.53	21.57	8.05	17.94	3.64	72.72	3.62	1	22.11	3.85
3	59.14	6.69	70.55	8.74	82.82	7.52	79.54	3.31	0.3	31.71	1.73
1	81.76	2.81	89.01	5.09	91.52	5.56	89.70	6.58	0.1	80.61	2.02
0.3	93.37	2.72	95.64	1.88	98.54	1.55	97.20	3.81	0.03	98.54	4.10
GI_{50}	3.251		4.595		5.390		14.460		GI_{50}	0.232	

NCI-H23

	3		4		5		6			Adriamycin	
Conc.(uM)	Mean	SD	Mean	SD	Mean	SD	Mean	SD		Mean	SD
30	−15.35	4.21	−16.80	2.97	−12.05	7.43	21.96	3.39	3	8.51	6.21
10	−6.83	1.38	23.67	5.41	31.21	5.12	59.17	5.57	1	19.97	1.60
3	68.21	3.80	77.08	8.24	79.44	2.80	75.44	1.77	0.3	40.04	4.28
1	89.92	1.99	85.49	7.76	88.22	5.48	89.55	4.19	0.1	89.17	2.72
0.3	96.53	4.83	94.67	6.05	95.54	4.25	95.90	2.05	0.03	94.97	1.79
GI_{50}	3.330		5.163		5.987		11.010		GI_{50}	0.284	

Table S17. Cytotoxicity of **3**–**6** against blood cancer cell lines.

**RPMI-1788**	**Doxorubicin**		**RPMI-1788**	**3**		**4**		**5**		**6**	
(µM)	MEAN	SD	(µM)	MEAN	SD	MEAN	SD	MEAN	SD	MEAN	SD
**0.100**	10.197	1.018	**30.00**	0.046	0.024	0.036	0.045	0.058	0.034	0.096	0.010
**0.030**	26.217	1.471	**10.00**	1.078	0.113	1.343	0.142	1.168	0.256	69.307	5.340
**0.010**	68.447	5.941	**3.00**	51.688	1.605	82.578	2.635	105.898	4.382	104.633	4.738
**0.003**	93.597	5.020	**1.00**	98.108	3.234	93.903	2.417	100.281	5.383	100.320	5.621
**0.001**	94.818	2.477	**0.30**	93.548	4.219	93.729	2.535	98.430	5.274	93.868	4.068
**GI$_{50}$**	**0.016**		**GI$_{50}$**	**3.056**		**4.193**		**8.015**		**11.060**	

**HL-60**	**Doxorubicin**		**HL-60**	**3**		**4**		**5**		**6**	
**(µM)**	MEAN	SD	(µM)	MEAN	SD	MEAN	SD	MEAN	SD	MEAN	SD
**0.100**	13.360	0.421	**30.00**	0.033	0.023	0.034	0.039	0.050	0.045	0.136	0.034
**0.030**	55.370	4.198	**10.00**	0.107	0.036	0.478	0.055	6.205	1.064	110.080	0.579
**0.010**	92.490	8.003	**3.00**	102.358	4.385	104.465	6.701	118.370	3.079	114.859	4.832
**0.003**	107.626	5.288	**1.00**	113.010	5.381	104.431	5.237	117.381	7.332	116.854	4.857
**0.001**	90.124	4.670	**0.30**	88.888	5.158	82.795	5.467	101.198	7.844	95.307	6.582
**GI$_{50}$**	**0.035**		**GI$_{50}$**	**6.835**		**7.997**		**8.144**		**24.920**	

**K562**	**Doxorubicin**		**K562**	**3**		**4**		**5**		**6**	
**(µM)**	MEAN	SD	**(µM)**	MEAN	SD	MEAN	SD	MEAN	SD	MEAN	SD
**1.000**	4.634	0.262	**30.00**	14.429	0.800	29.791	0.461	0.636	0.089	4.584	0.517
**0.300**	31.075	2.595	**10.00**	77.214	1.640	79.302	1.391	57.169	4.767	103.392	2.679
**0.100**	53.933	3.664	**3.00**	101.037	1.394	98.386	1.519	104.955	1.034	103.340	1.558
**0.030**	75.709	4.137	**1.00**	100.919	2.067	100.791	3.750	104.711	1.739	98.308	7.020
**0.010**	81.440	4.826	**0.30**	97.377	3.240	97.340	4.917	96.288	5.593	85.649	3.497
**GI$_{50}$**	**0.104**		**GI$_{50}$**	**15.650**		**19.580**		**10.380**		**22.760**	

**NALM6**	**Doxorubicin**		**NALM6**	**3**		**4**		**5**		**6**	
**(µM)**	MEAN	SD	**(µM)**	MEAN	SD	MEAN	SD	MEAN	SD	MEAN	SD
**0.100**	-0.006	0.047	**30.00**	0.020	0.025	0.023	0.019	0.050	0.033	0.064	0.005
**0.030**	0.115	0.281	**10.00**	0.007	0.035	0.079	0.030	0.142	0.061	55.434	2.059
**0.010**	59.679	5.890	**3.00**	26.725	0.936	87.406	5.082	101.468	2.532	99.714	2.453
**0.003**	93.637	2.553	**1.00**	100.761	2.073	98.103	0.321	101.480	1.806	102.083	1.740
**0.001**	87.190	6.668	**0.30**	92.282	4.775	92.299	4.144	100.581	2.023	97.794	2.882
**GI$_{50}$**	**0.011**		**GI$_{50}$**	**2.680**		**3.959**		**7.574**		**10.360**	

**Raji**	**Doxorubicin**		**Raji**	**3**		**4**		**5**		**6**	
**(µM)**	MEAN	SD	**(µM)**	MEAN	SD	MEAN	SD	MEAN	SD	MEAN	SD
**0.100**	11.461	0.947	**30.00**	0.051	0.025	3.744	0.164	0.080	0.043	0.144	0.013
**0.030**	39.143	1.886	**10.00**	14.219	2.429	26.051	0.788	30.303	6.189	105.754	1.953
**0.010**	74.960	1.895	**3.00**	84.660	1.233	93.623	1.354	108.681	2.732	108.684	0.965
**0.003**	101.719	2.572	**1.00**	99.653	3.629	98.601	1.755	107.292	2.413	109.339	0.770
**0.001**	99.286	3.943	**0.30**	94.148	4.094	92.298	4.880	98.905	3.957	97.063	2.959
**GI$_{50}$**	**0.022**		**GI$_{50}$**	**5.392**		**7.110**		**9.221**		**24.500**	

**RPMI-8402**	**Doxorubicin**		**RPMI-8402**	**3**		**4**		**5**		**6**	
**(µM)**	MEAN	SD	**(µM)**	MEAN	SD	MEAN	SD	MEAN	SD	MEAN	SD
**0.300**	33.759	1.929	**30.00**	27.452	1.455	18.178	1.665	7.637	0.709	23.369	0.983
**0.100**	58.850	3.023	**10.00**	74.132	2.211	73.668	2.092	74.490	5.620	97.731	1.458
**0.030**	75.610	8.068	**3.00**	99.631	3.743	98.392	1.787	101.308	1.227	97.718	1.771
**0.010**	90.209	3.086	**1.00**	99.732	2.584	97.690	2.952	99.398	2.453	95.968	0.913
**0.003**	89.791	3.170	**0.30**	96.661	2.648	94.690	3.840	98.338	3.393	94.820	2.602
**GI$_{50}$**	**0.139**		**GI$_{50}$**	**17.820**		**15.630**		**13.900**		**23.040**	

**U266**	**Doxorubicin**		**U266**	**3**		**4**		**5**		**6**	
**(µM)**	MEAN	SD	**(µM)**	MEAN	SD	MEAN	SD	MEAN	SD	MEAN	SD
**0.300**	9.923	0.414	**30.00**	0.042	0.033	0.058	0.033	0.063	0.045	0.182	0.025
**0.100**	51.987	4.110	**10.00**	0.310	0.139	29.980	1.570	26.742	1.772	102.539	5.370
**0.030**	84.719	4.527	**3.00**	93.687	6.975	97.423	2.352	113.707	4.762	111.067	3.658
**0.010**	102.195	8.005	**1.00**	97.602	3.489	97.133	2.327	111.706	5.649	96.530	9.324
**0.003**	90.774	2.948	**0.30**	87.654	3.935	85.145	2.821	107.188	8.264	97.702	4.145
**GI$_{50}$**	**0.098**		**GI$_{50}$**	**4.415**		**7.969**		**9.050**		**24.670**	

**WSU-DLCL2**	**Doxorubicin**		**WSU-DLCL2**	**3**		**4**		**5**		**6**	
**(µM)**	MEAN	SD	**(µM)**	MEAN	SD	MEAN	SD	MEAN	SD	MEAN	SD
**0.100**	5.777	0.415	**30.00**	0.072	0.029	3.048	0.440	0.094	0.044	0.211	0.020
**0.030**	16.687	1.341	**10.00**	26.850	2.030	63.067	2.373	47.912	1.776	104.970	1.435
**0.010**	77.048	1.091	**3.00**	100.271	0.833	100.551	1.645	103.240	0.757	102.838	0.171
**0.003**	99.016	1.246	**1.00**	101.470	1.936	98.579	1.126	102.294	0.502	101.852	2.173
**0.001**	92.317	2.759	**0.30**	92.887	2.748	91.771	1.994	97.801	3.257	97.349	1.977
**GI$_{50}$**	**0.016**		**GI$_{50}$**	**8.966**		**11.550**		**9.936**		**24.130**	
